# Supplementary material for: AI-engineered AAV capsid enables intravitreal delivery for the treatment of diverse retinal degenerations
Source: Mol Ther Adv. 2026 Jul 9;34(3):201806. doi: 10.1016/j.omta.2026.201806 (PMC13392946; doi:10.1016/j.omta.2026.201806)
Supplement: Document S1. Figures S1–S10 and Tables S1–S4 [file mmc1.pdf]

OMTA, Volume *34*

Supplemental information

AI-engineered AAV capsid enables intravitreal delivery for the treatment of diverse retinal degenerations

Mochen Cui, Huaqing Liu, Lei Cai, Qian Zhang, Li Yuan, Cui Gao, Chunlian Li, Jianfei Xi, Yongkui Li, Chenguang Wu, Qin Zheng, Lei Liu, Peiyi Chen, Shuxian Zhou, Xing Mou, Joseph Wekselblatt, Yu Zhang, Lance Han, and Sheng Ren

Table S1. Key primer table.

Name	Sequence	Targeted Element
AAV2 Cap Insertion F	GACAACCAATCCCGTGGCTA	AAV Library
AAV2 Cap Insertion R	AGACCATGCCTGGAAGAACG	
AAV2 Barcoded eGFP F	AATGATACGGCGACCACCGAGATCTACACTCTTTCCCTACACGACGCTCT	Barcoded eGFP
AAV2 Barcoded eGFP R	CAAGCAGAAGACGGCATACGAGATATCACGTTGTGACTGGAGTTCAGACGT GTGCTCTTCCGATCTCACATGGTCCTGCTGGAGTT	
AAV2 eGFP F (Titer)	GCTACCCCGACCACATGAAG	eGFP
AAV2 eGFP R (Titer)	CGGGTCTTGTAGTTGCCGT	
AAV2 LUC F	TGGGCGCGTTATTTATCGGA	Luciferase
AAV2 LUC R	CACTACGGTAGGCTGCGAAA	
hGUCY2D-129 F	CCAAACAACATCTGCGGTGG	*hGUCY2D*
hGUCY2D-129 R	TTGCATGTGAAGTAGCCGGT	
hGUCY2D-129 VIC	CCCATCCCAACCACCAGGAGGAAGCCA	
Anti-VEGFA 180 F	GGTGACGGTGTCATGGAACT	Anti-VEGFA
Anti-VEGFA 180 FAM	CCTCAGCAGCGTGGTGACCGTGCC	
Anti-VEGFA 180 R	TTGTCCACCTTGGTGTTGCT	
hVEGFA-123 F	CTTGCCTTGCTGCTCTACCT	*hVEGFA*
hVEGFA-123 R	GCAGTAGCTGCGCTGATAGA	
mVegfa-195 F	CACGACAGAAGGAGAGCAGA	m*Vegfa*
mVegfa-195 R	CACTCCAGGGCTTCATCGTT	
mGapdh F	AGGTCGGTGTGAACGGATTTG	m*Gapdh*
mGapdh R	TGTAGACCATGTAGTTGAGGTCA	

Table S2. Insertion sequences for monkey-targeting.

Capsid ID	Insertion Sequence
PN168	DEEEIRTTNPVATEQYGSVSTNLQRGNAAVRFDGTERAAR
PN169	DEEEIRTTNPVATEQYGSVSTNLQRGNRQYSDAVRAER
PN170	SEEEIRTTNPSATEQYGVAATNLQKGNR
PN171	DEEEIRTTNPVATEQYGSVSTNLQRGNAAILGTVKHAR
PN172	DEEEIRTTNPVATEQYGSVSTNLQRGNAADRETGRPAR
PN173	DEEEIRTTNPVATEQYGSVSTNLQRGNAALNSMAKPAR
PN174	DEEEIRTTNPVATEQYGSVSTNLQRGNTARDGVEGSRAAR
PN175	DEEEIRTTNPVATEMYGEVSRNLQRGER
PN176	DEEEIRTTNPVAYEPYGSVSTNLQRGNQAEREYNRPAR
PN177	DEEEIRTTNPVATEQYGSVSTNLQRGNYADSSSGRPAR
PN178	DEEEIRTTNPVATEQYGSVSTNLQRGNLANYETPRPAR
PN179	DEEEIRTTNPVATEQYGEVSRNLQRGER
PN180	DEEEIRTTNPVATEQYGSVSTNLQRGNAANAMLGRPAR
PN181	DEEEIRTTNPVATEQYGSVSTNLQRGNAAHPEVGRPAR
PN182	DEEEIRTTNPVATEQYGSVSTNLQRGNAAHNEQMRPGR
PN183	DEEEIRTTNPVATEQYGSVSTNLQRGNTATEDRSRPAR
PN184	DEEEIRTTNPVATEQYGSVSTNLQRGNSALNATSRPAR
PN185	DEEEIRTTNPVATEQYGYVSTNVQRTGR
PN186	DEEEIRTTNPVATEQYGSVSTNLQRGNAAPGVNTKPAR
PN187	DEEEIRTTNPVATEQYGSVSTNLQRGNRNAGEDHSLRAAR
PN188	DEQEIATTNPVATEQYGVVSNNLQRTNR
PN189	DEEEIRTTNPVATEQYGSVSTNLQRGNATAPNSVARPAR
PN190	DEEEIRTTNPVATEQYGSVSTNLQRGNQTINSAPRPAR
PN191	DEEEIRTTNPVATEQYGSVSTNLQRQTR
PN192	DEEEIRTTNPVATEQYGSVSTNLQRGNAAVRFNGTERAAR
PN193	DEEEIRTTNPVATEQYGSVSTNLQRGNAIHPEVGRPAR
PN194	DEEEIRTTNPVATEQYGSVSTNLQRGNAASLSPTRAAR
PN195	DEEEIRTTNPVATEQYGYVSTNVQRGGR
PN196	DEEEIRTTNPVATEQYGSVSTNLQRGNTAQDGQGRPAR
PN197	SEEEIRTTNPSATEQYGSVSTNLQRGNR
PN198	DEEEIRTTNPVATEQYGSVSTNLQRGNAATLSSTVPAR
PN199	DEEEIRTTNPVATEQYGSVSTNLQRGNLADTDKFRQAR
PN200	DEEEIRTTNPVATEQYGSVSTNLQRGNTGSTPSRSGPGLSR
PN201	DEEEIRTTNPVATEQYGSVSTNLQRGNTAQDGTSRRER
PN202	DEEEIRTTNPVATEQYGSVSTNLQRGNAAPNSVARPAR
PN203	DEEEIRTTNPVATEQYGSVSTNLQRGNAAHNDTPRPAR
PN204	DEEEIRTTNPVATEQYGSVSTNLQRGNAAANSPTTQRAAR
PN205	DEEEIRTTNPVATEQYGSVSTNLQRGNLAHNEFSRPAR
PN206	DEEKIRTTNPVATEQYGSVSTNLQRGNR
PN207	DEEEIRTTNPVATEQYGSVSSNLQRGNAAHNDTPRPAR

Table S3. Insertion sequences for mouse-targeting.

Capsid ID	Insertion Sequence
PM241	TGRDRLGLSGLS
PM242	TGTGLTIGRGLS
PM243	TGTSTFGASRGLS
PM244	TGTGAFAQRGLS
PM245	TGGSYLPSPSRGLS
PM246	TGFSLPGSSRGLS
PM247	TGGASYGLPQSRGLS
PM248	TGSQFAPARGLS
PM249	TGSRQAYSTGLS
PM250	TGGLLVSSRGLS
PM251	TGTVSGIGRGLS
PM252	TGSSLLGSSSRGLS
PM253	TGGMVSGLSRGLS
PM254	TGSQLLGSRGLS
PM255	TGLSTLGNRGLS
PM256	TGYSSLPSGSRGLS
PM257	TGRPQAYSSGLS
PM258	TGTTFTAQRGLS
PM259	TGQTLGMSRGLS
PM260	TGGAAGLSRGLS
PM261	TGSLLQLSGSRGLS
PM262	TGQGPLSSRGLS
PM263	TGFSPLTPGRGLS
PM264	TGGTLSISRGLS
PM265	TGQLGSLSRGLS
PM266	TGGSFTGVGRGLS
PM267	TGANILGSRGLS
PM268	TGSRTGFSGGLS
PM269	TGSSSLLGTSRGLS
PM270	TGNPLLQTSRGLS
PM271	TGPSSSPFTGRGLS
PM272	TGYLPTGQSTRGLS
PM273	TGGTMLGTRGLS
PM274	TGSGAVPSLHRGLS
PM275	TGSLLGVGSRGLS
PM276	TGRGAGYPSGLS
PM277	TGLTTGLSRGLS
PM278	TGSTIGLGSRGLS
PM279	TGSSSFTPSRGLS
PM280	TGSMLLSSGRGLS

PM281	TGSQFGQPMSRGLS
PM282	TGVSALGGMRGLS
PM283	TGASQAFPRGLS
PM284	TGGGMATGLSRGLS
PM285	TGGLLSATRGLS
PM286	TGVTPTGSRGLS
PM287	TGTAIGGSRGLS
PM288	TGHTIGPIPGRGLS
PM289	TGSIAGGSLRGLS
PM290	TGGALSLSSRGLS
PM054	TGTSTFGASRGLS

Table S4. Key resource table.

Reagent or Resource	Source/Vendor	Identifier
**Plasmid Construction and AAV Packaging**		
NheI-HF	New England Biolabs	Cat# R3131
NotI-HF	New England Biolabs	Cat# R3189
pAAV2/2	Addgene	Cat# Plasmid #104963
pAAV CAGG eGFP	Addgene	Cat# Plasmid #107707
BsrGI-HF	New England Biolabs	Cat# R3575
XhoI	New England Biolabs	Cat# R0146
Double-stranded DNA Fragments Containing Barcodes	Integrated DNA Technologies	N/A
Gibson Assembly Master Mix	New England Biolabs	Cat# E2611
EcoRI-HF	New England Biolabs	Cat# R3101
BglII	New England Biolabs	Cat# R0144
Double-stranded DNA Fragments Containing Luciferase cDNA	Integrated DNA Technologies	N/A
Double-stranded DNA fragments containing anti-VEGFA cDNA	Integrated DNA Technologies	N/A
PEI MAX - Transfection Grade Linear Polyethylenimine Hydrochloride	Polysciences	Cat# 24765
pAdDeltaF6	Addgene	Cat# Plasmid #112867
Benzonase Nuclease	Merck Millipore	Cat# 70746
Amicon Ultra Centrifugal Filter, 100 kDa MWCO	Merck Millipore	Cat# UFC8100
**Nucleic Acid Isolation, Amplification and Assessment**		
Q5 High-Fidelity DNA Polymerase	New England Biolabs	Cat# M0491
QIAquick PCR Purification Kit for PCR Cleanup	Qiagen	Cat# 28104
TRIzol Reagent	Invitrogen	Cat# 15596018CN
RNeasy Mini Kit	Qiagen	Cat# 74104
DNA-free DNA Removal Kit	Invitrogen	Cat# AM1906
RevertAid First Strand cDNA Synthesis Kit	Thermo Scientific	Cat# K1621
10x Chromium Single Cell 3' kits (multiple components)	10x Genomics	Cat# 1000075<br>Cat# 1000092<br>Cat# 1000073<br>Cat# 1000074<br>Cat# 120262
SPRIselect Bead-Based Reagent	Beckman Coulter Diagnostics	Cat# B23318
TaqMan Fast Advanced Master Mix	Applied Biosystems	Cat# 4444556
Digital PCR Buffer	RainSure Scientific	Cat# 3005
Transduction Inhibition Rate		
Dulbecco's Modified Eagle's Medium	Corning	Cat# 10-013-CVR
Fetal Bovine Serum, Premium	Gibco	Cat# A5670701
Trypsin (2.5%), No Phenol Red	Gibco	Cat# 15090046
**Heparin Binding Assay**		
Heparin sodium salt from porcine intestinal mucosa	Sigma-Aldrich	Cat# H3149
TrypLE™ Express Enzyme (1X)	Gibco	Cat# 12604039
***in vivo* Procedures**		
Small Hub Removable Needle, 33-gauge	Hamilton Company	Cat# 7803-05
2.5 μl Microliter Syringe	Hamilton Company	Cat# 7632-01
BD Ultra-Fine U-100 Syringe	Becton Dickinson	Cat# BD 328291
AK-FLUOR (Fluorescein Injection)	Akorn	Cat# 17478-253-10
D-Luciferin, Sodium Salt	Yeasen	Cat# 40901ES03
Ranibizumab (Lucentis)	Novartis Pharma Schweiz AG	Cas ID 347396-82-1
**Immunostaining**		
Tissue-Tek O.C.T. Compound	Sakura Finetek	Cat# 4583

SuperFrost Ultra Plus GOLD Adhesion Slides	Epredia	Cat# K5800AMNZ72
Bovine Serum Albumin, Lyophilized Powder	Sigma-Aldrich	Cat# A9418
GFP Polyclonal Antibody, Alexa Fluor 488	Invitrogen	Cat# A-21311
DAPI	Thermo Scientific	Cat# 62248
ProLong Gold Antifade Mountant	Invitrogen	Cat# P36930
**Experimental Models: Cells/Animals**		
Cell: HEK293T	ATCC	Cat# CRL-3216
Mouse: *C57BL/6J*	Charles River	RRID: IMSR_JAX:000664
Mouse: *BALB/cJ*	Charles River	RRID: IMSR_JAX:000651
Mouse: *C57BL/6JCya-Gucy2e*^{em1}*Gucy2f*^{em1}*/Cya*	Cyagen	Cat# C001928
Mouse: *C57BL/6JCya-Tg(bRho-VEGFA)/Cya*	Cyagen	Cat# C001395
Monkey: Cynomolgus Monkey (*Macaca fascicularis*)	Landao-Bio	N/A
**Software and Algorithms**		
Python 3.0	Public Domain	https://www.python.org/
Scanpy	Public Domain	https://github.com/scverse/scanpy
RDKit	Public Domain	https://www.rdkit.org/docs/index.html
NovoExpress 1.5.0	Agilent Technologies	https://www.agilent.com.cn/en/product/research-flow-cytometry/flow-cytometry-software/novocyte-novoexpress-software-1320805
cellSens 3.2	Olympus	N/A
LAS X 3.10.0	Leica	https://www.leica-microsystems.com/products/microscope-software/p/leica-las-x-ls/
LAS X 4.7.0	Leica	https://www.leica-microsystems.com/products/microscope-software/p/leica-las-x-ls/
AniView 1.00.00621	BLT-Imaging	N/A
Imaris 10	Bitplane	https://imaris.oxinst.com
ImageJ 1.54f	Public Domain	http://imagej.org
GeneCount Analysis System software v1.63.0222	RainSure Scientific	N/A
Image-Pro Plus 6.0	Media Cybernetics	https://mediacy.com/image-pro/
Prism 10	GraphPad Software	https://www.graphpad.com/scientific-software/prism/

Figure S1

A

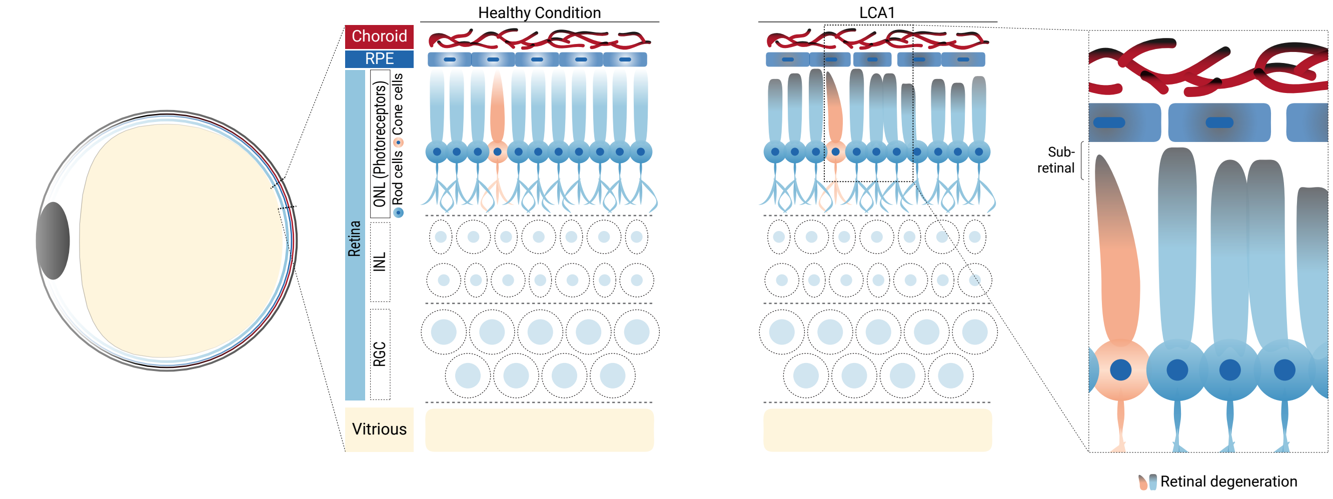
Healthy Condition
LCA1
Choroid
RPE
Retina
ONL (Photoreceptors)
Rod cells
Cone cells
INL
RGC
Vitrious
Sub-retinal
Retinal degeneration

B

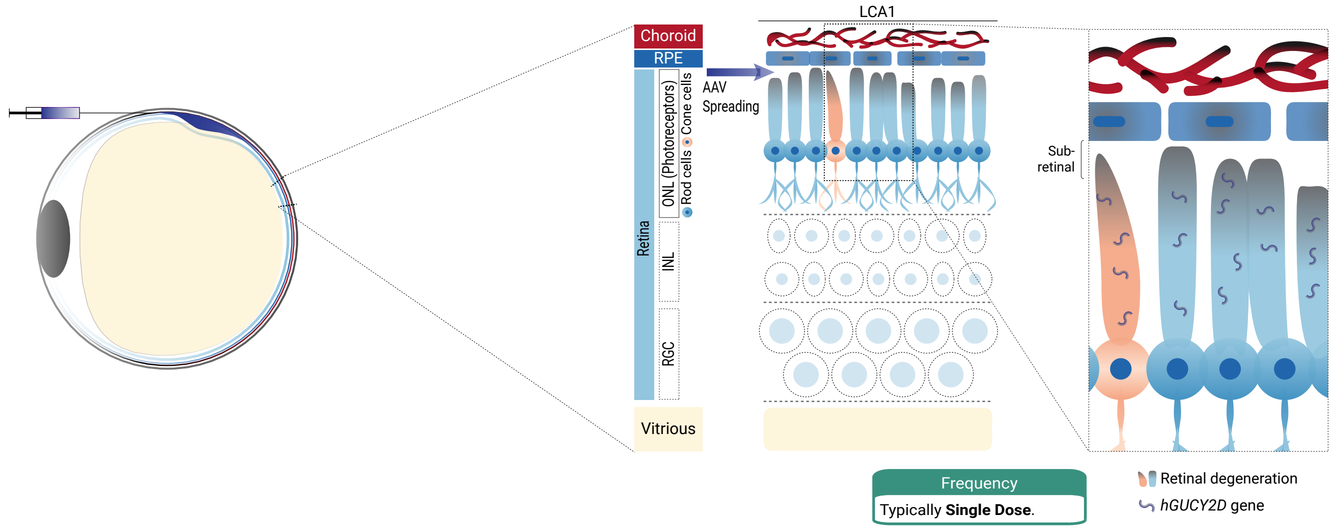
LCA1
Choroid
RPE
AAV Spreading
Retina
ONL (Photoreceptors)
Rod cells
Cone cells
INL
RGC
Vitrious
Sub-retinal
Frequency
Typically **Single Dose**.
Retinal degeneration
hGUCY2D gene

Figure S1. Schematic representation of LCA1 pathology and AAV-mediated hGUCY2D therapy for LCA1. A, Schematic representation of LCA1. **B**, Schematic representation of AAV-mediated hGUCY2D therapy for LCA1. RGC, retinal ganglion cells; INL, inner nuclear layer; ONL, outer nuclear layer; RPE, retinal pigment epithelium; LCA1, Leber's congenital amaurosis type 1.

Figure S2

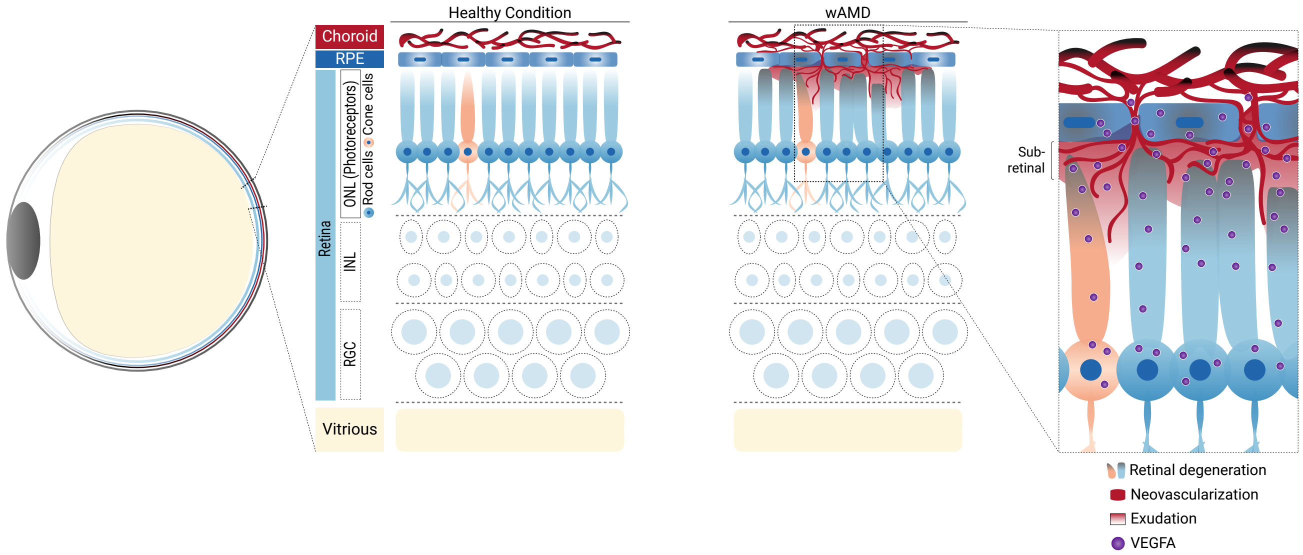
A
Healthy Condition
wAMD
Choroid
RPE
Retina
ONL (Photoreceptors)
Rod cells
Cone cells
INL
RGC
Vitrious
Sub-
retinal
Retinal degeneration
Neovascularization
Exudation
VEGFA

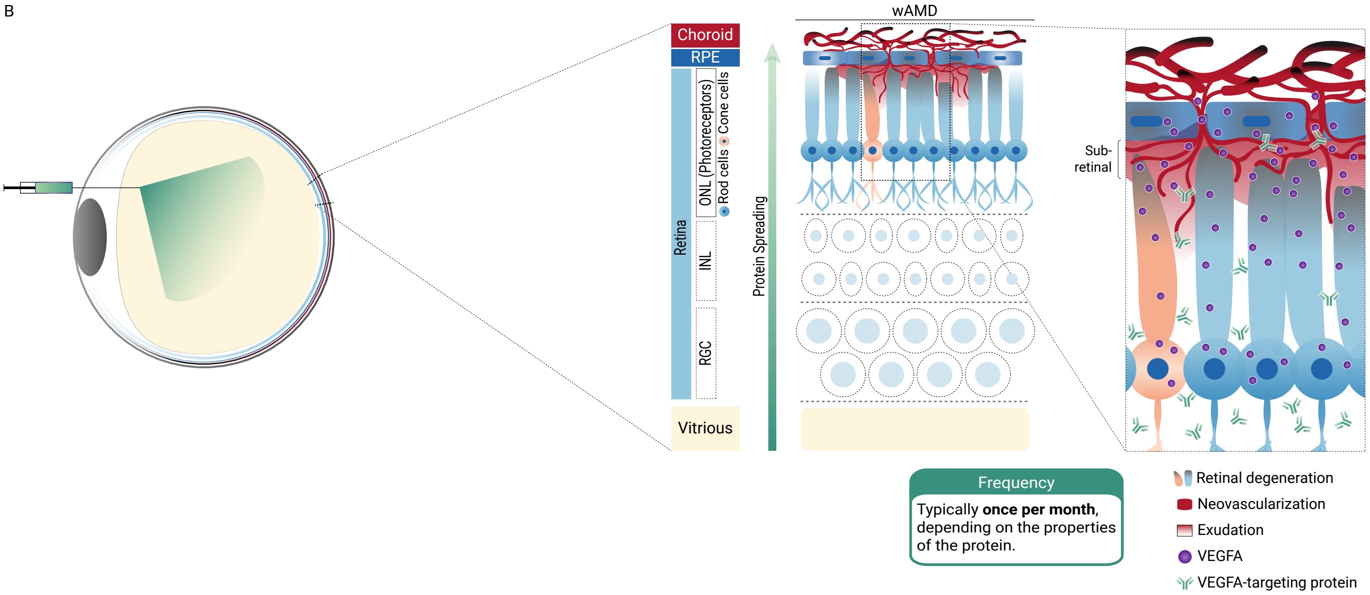
B
wAMD
Choroid
RPE
Retina
ONL (Photoreceptors)
Rod cells
Cone cells
INL
RGC
Vitrious
Protein Spreading
Sub-
retinal
Frequency
Typically once per month, depending on the properties of the protein.
Retinal degeneration
Neovascularization
Exudation
VEGFA
VEGFA-targeting protein

Figure S2. Schematic representation of wAMD pathology and anti-VEGFA protein drug therapy for wAMD. A, Schematic representation of wAMD. **B**, Schematic representation of anti-VEGFA therapy for wAMD with protein drugs. RGC, retinal ganglion cells; INL, inner nuclear layer; ONL, outer nuclear layer; RPE, retinal pigment epithelium; wAMD, wet age-related macular degeneration.

Figure S3

A

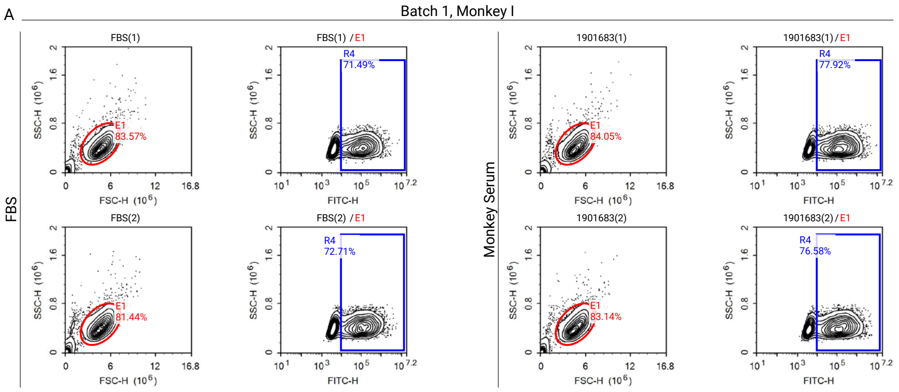

B

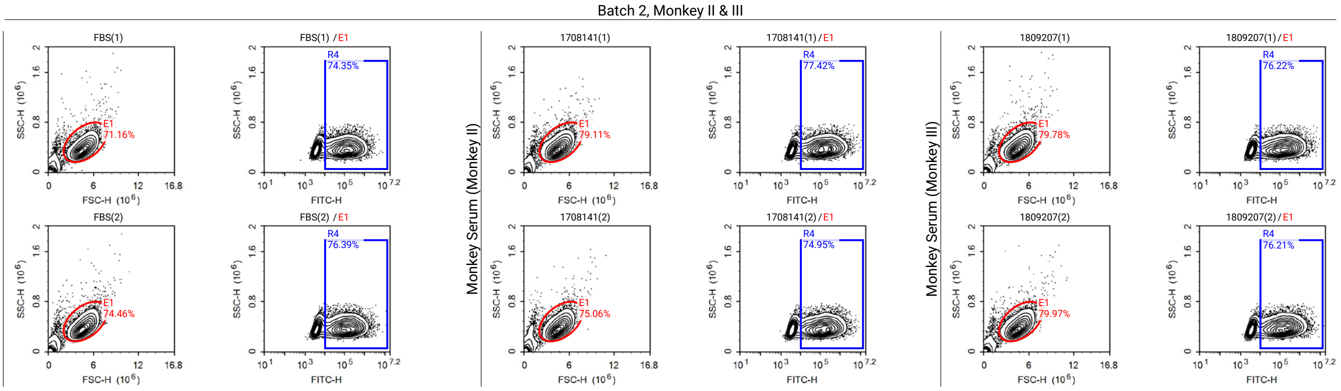

C

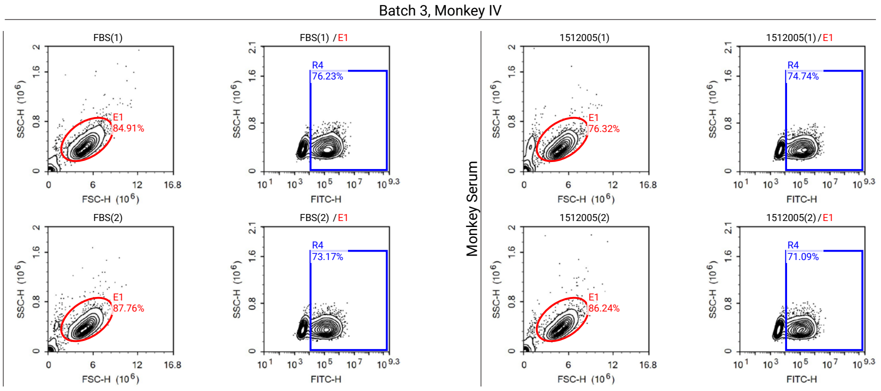

Figure S3. FACS analysis of transduction inhibition rate on AAV2.WT. A The population of GFP^+ cells (R4) after incubation with eGFP-encoding AAV2 pretreated with FBS and monkey serum from monkey I. **B**, The population of GFP^+ cells (R4) was examined after incubation with eGFP-encoding AAV2 pretreated with FBS and monkey serum from monkeys II and III. Notably, the FBS group was utilized for both monkeys since their samples were detected within the same batch. **C**, The population of GFP^+ cells (R4) after incubation with eGFP-encoding AAV2 pretreated with FBS and monkey serum from monkey IV. Two serum samples from each monkey were included, along with corresponding controls (FBS). The order is indicated in parentheses next to the panel title.

Figure S4

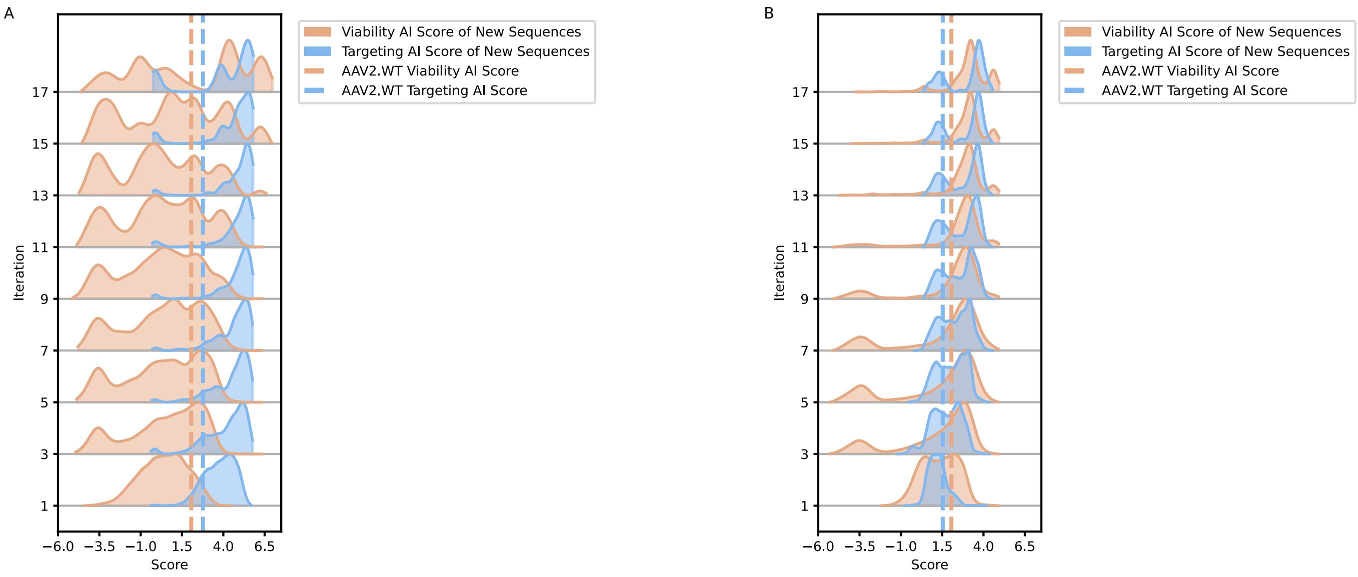

Mouse Viability and Targeting Models

Monkey Viability and Targeting Models

Figure S4. Iterative process for new sequence generation. A, Iteration of the mouse framework. **B**, Iteration of the monkey framework.

Figure S5

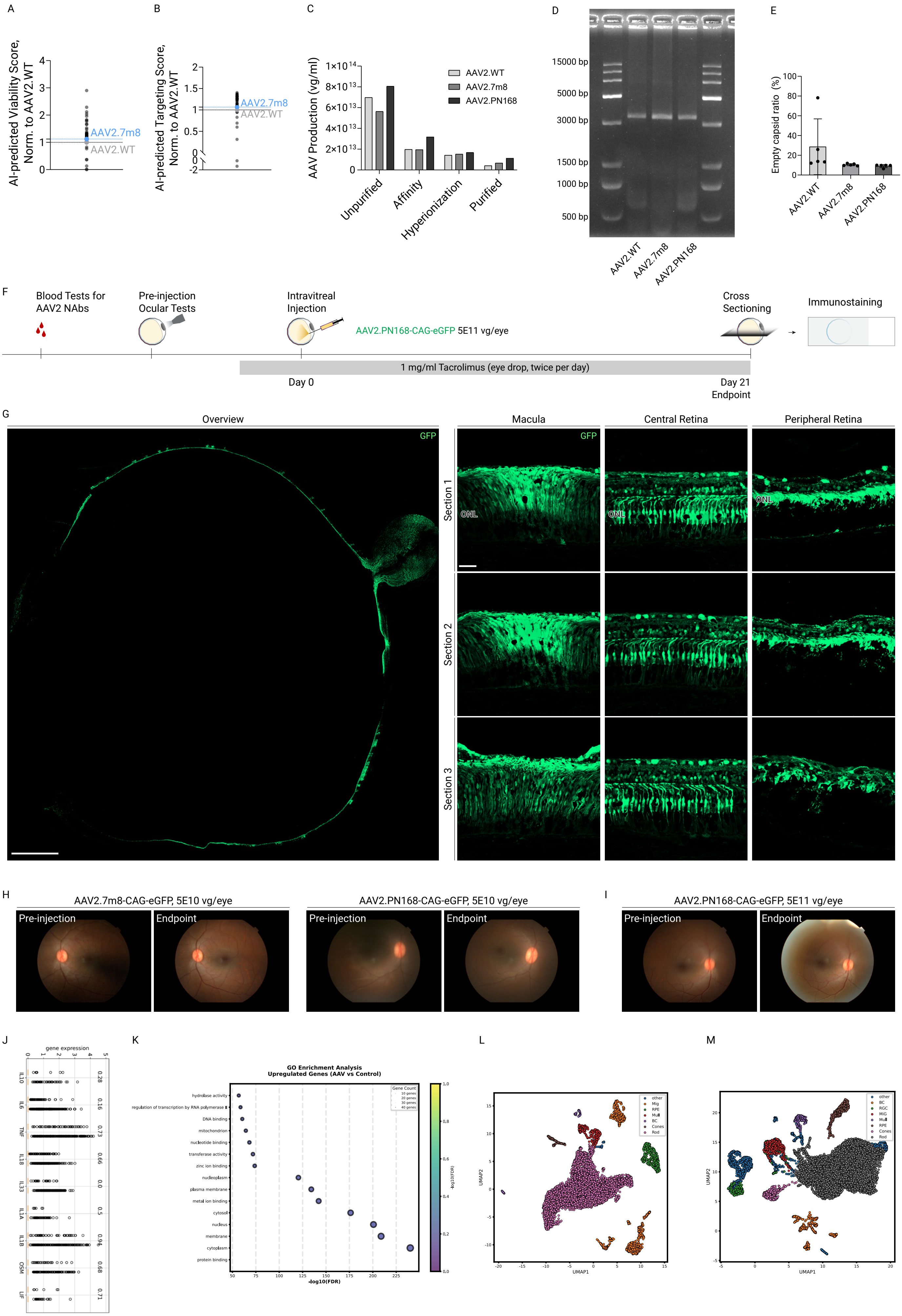

A
AI-predicted Viability Score, Norm. to AAV2.WT
AAV2.7m8
AAV2.WT
B
AI-predicted Targeting Score, Norm. to AAV2.WT
AAV2.7m8
AAV2.WT
C
AAV Production (vg/ml)
AAV2.WT
AAV2.7m8
AAV2.PN168
Unpurified
Affinity
Hyperionization
Purified
D
15000 bp
5000 bp
3000 bp
1500 bp
1000 bp
500 bp
AAV2.WT
AAV2.7m8
AAV2.PN168
E
Empty capsid ratio (%)
AAV2.WT
AAV2.7m8
AAV2.PN168
F
Blood Tests for AAV2 NAbs
Pre-injection Ocular Tests
Intravitreal Injection
AAV2.PN168-CAG-eGFP 5E11 vg/eye
Cross Sectioning
Immunostaining
1 mg/ml Tacrolimus (eye drop, twice per day)
Day 0
Day 21 Endpoint
G
Overview
Macula
Central Retina
Peripheral Retina
GFP
Section 1
Section 2
Section 3
ONL
H
AAV2.7m8-CAG-eGFP, 5E10 vg/eye
AAV2.PN168-CAG-eGFP, 5E10 vg/eye
I
AAV2.PN168-CAG-eGFP, 5E11 vg/eye
Pre-injection
Endpoint
J
gene expression
IL10
IL6
TNF
IL18
IL33
IL1A
IL1B
OSM
LIF
K
GO Enrichment Analysis
Upregulated Genes (AAV vs Control)
hydrolase activity
regulation of transcription by RNA polymerase II
DNA binding
mitochondrion
nucleotide binding
transferase activity
zinc ion binding
nucleoplasm
plasma membrane
metal ion binding
cytosol
nucleus
membrane
cytoplasm
protein binding
-log10(FDR)
Gene Count
L
UMAP1
UMAP2
M
UMAP1
UMAP2

Figure S5. Performance of AAV2.PN168 in AI-prediction, production, and intravitreal injection at various doses. A, AI-predicted viability score of the 43 AI-generated AAV2-derived variants, normalized to AAV2.WT, semi-transparent grey dots indicate AI-generated capsids, solid grey dot indicates AAV2.WT, solid blue dot indicates AAV2.7m8. **B**, AAV production at different stages. **C**, Gel electrophoresis of lysed AAVs, showing bands for AAV-packaged barcoded eGFP. **D**, Empty capsid ratio of purified AAVs. **F**, Experimental design. **G**, Representative confocal micrographs of total retinal cross sections and different retinal regions from monkey eyes receiving different high-dose AAV2.PN168. ONL is indicated in the graphs. **H**&**I**, Fundoscopy of the monkey eyes, before and after the injection of corresponding AAVs and dosages. **J**, Comparison of gene expression across immune-associated factors between AAV-transduced and non-transduced retinal cells, showing no significant differences. **K**, Gene Ontology enrichment analysis for both AAV-transduced and non-transduced retinal cells, showing no upregulation of immune-associated pathways. **L**, UMAP plot showing monkey retinal cells. **M**. UMAP plot depicting mouse retinal cells. n = 5 regions from EM (**E**), n = 1 eye (**F**-**H**), 7,742 retinal cells were analyzed (**J**-**L)**, 18,577 retinal cells were analyzed (**M**). Dot plots show mean (SD). Scale bars: **i** = 2000 µm (overview), 50 µm (individual retinal regions). For fundus images, all images were obtained using a fundus camera at a fixed magnification and focal length, ensuring comparable coverage and locations across different groups, no scale was indicated by the imaging system. ONL, outer nuclear layer.

Figure S6

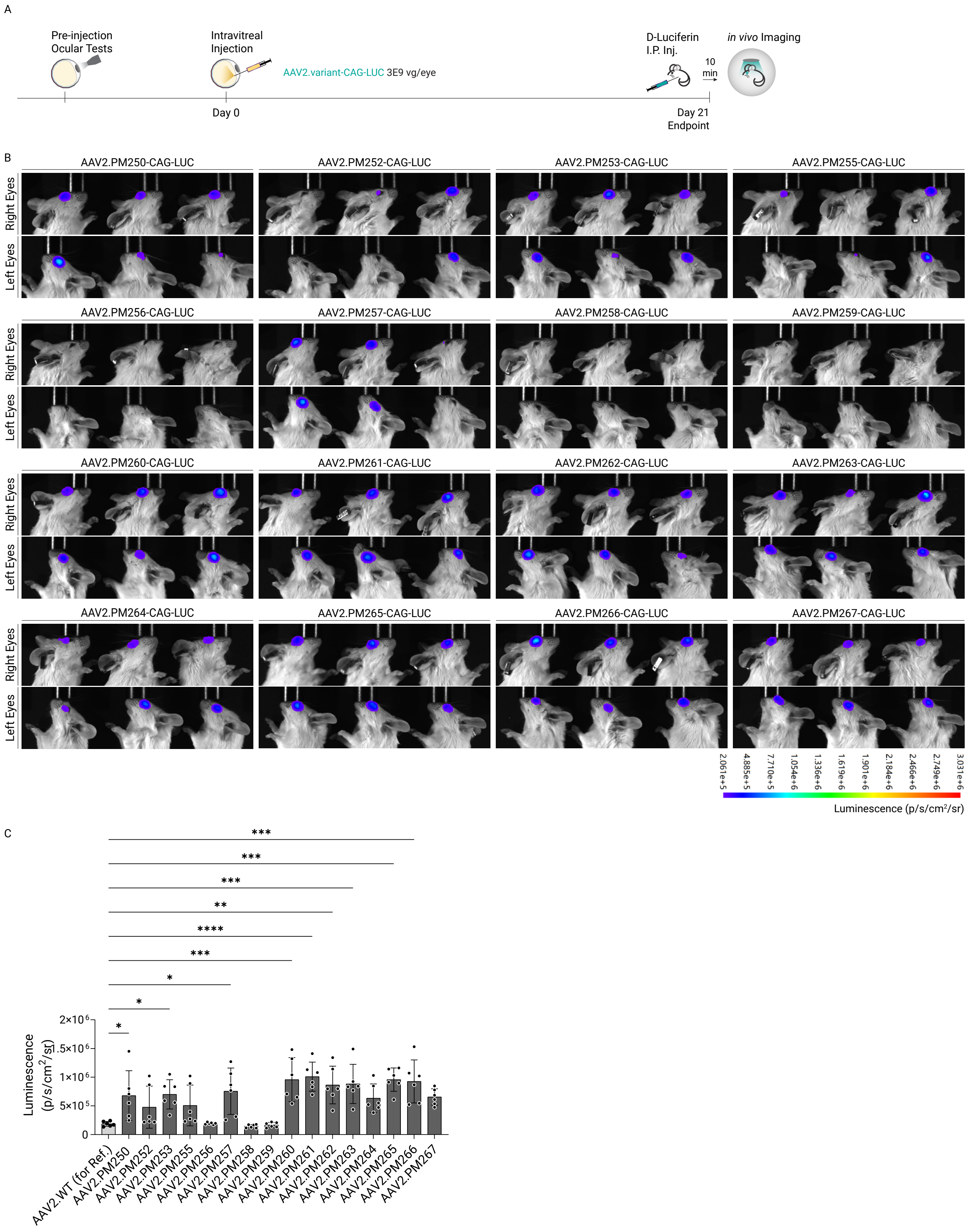
A
Pre-injection
Ocular Tests
Intravitreal
Injection
AAV2.variant-CAG-LUC 3E9 vg/eye
Day 0
D-Luciferin
I.P. Inj.
10
min
in vivo Imaging
Day 21
Endpoint
B
AAV2.PM250-CAG-LUC
AAV2.PM252-CAG-LUC
AAV2.PM253-CAG-LUC
AAV2.PM255-CAG-LUC
AAV2.PM256-CAG-LUC
AAV2.PM257-CAG-LUC
AAV2.PM258-CAG-LUC
AAV2.PM259-CAG-LUC
AAV2.PM260-CAG-LUC
AAV2.PM261-CAG-LUC
AAV2.PM262-CAG-LUC
AAV2.PM263-CAG-LUC
AAV2.PM264-CAG-LUC
AAV2.PM265-CAG-LUC
AAV2.PM266-CAG-LUC
AAV2.PM267-CAG-LUC
Right Eyes
Left Eyes
2.061e+5
4.885e+5
7.710e+5
1.054e+6
1.336e+6
1.619e+6
1.901e+6
2.184e+6
2.466e+6
2.749e+6
3.031e+6
Luminescence (p/s/cm²/sr)
C
***
***
***
**
****
***
*
*
*
Luminescence (p/s/cm²/sr)
2×10⁶
1.5×10⁶
1×10⁶
5×10⁵
0
AAV2.WT (for Ref.)
AAV2.PM250
AAV2.PM252
AAV2.PM253
AAV2.PM255
AAV2.PM256
AAV2.PM257
AAV2.PM258
AAV2.PM259
AAV2.PM260
AAV2.PM261
AAV2.PM262
AAV2.PM263
AAV2.PM264
AAV2.PM265
AAV2.PM266
AAV2.PM267

Figure S6. Luciferase-based *in vivo* imaging of mice treated with AAV2.variant-CAG-LUC. A, Experimental design. **B**, *in vivo* imaging following D-luciferin injection into mice administered with different luciferase-expressing AAV2-derived variants. **C**, Quantification of luminescence from the ocular region upon D-luciferin administration. n = 6 eyes (**A**-**C**). Dot plots show mean (SD). One-way ANOVA followed by Dunnett's multiple comparisons test (**C**: $F_{(16, 85)} = 6.739$, $p < 0.0001$). *p<0.05, **p<0.01, ***p<0.001, ****p<0.0001. n.s. - no significant difference.

Figure S7

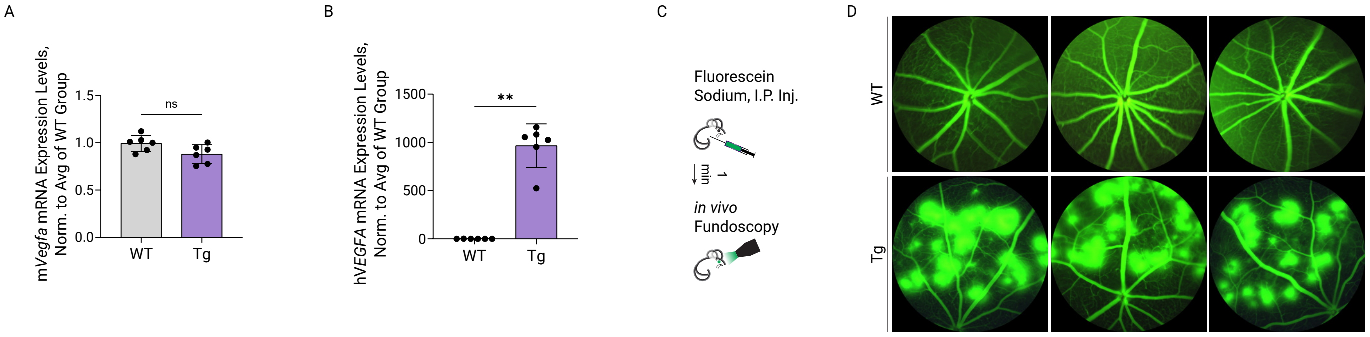
A
mVegfa mRNA Expression Levels, Norm. to Avg of WT Group
1.5
1.0
0.5
0.0
ns
WT
Tg
B
hVEGFA mRNA Expression Levels, Norm. to Avg of WT Group
1500
1000
500
0
**
WT
Tg
C
Fluorescein Sodium, I.P. Inj.
1 min
in vivo Fundoscopy
D
WT
Tg

Figure S7. Validation of *hVEGF-TG* mice. A, m*Vegfa* mRNA expression, detected using retinal tissue extracted from WT and *hVEGFA-TG* mice. **B**, h*VEGFA* mRNA expression, detected using retinal tissue extracted from WT and *hVEGFA-TG* mice. **C**, Experimental design. **D**, Representative micrographs obtained via *in vivo* fundoscopy upon fluorescein sodium injection. n = 6 eyes (**A**&**B**), n = 3 eyes (**c**). Dot plots show mean (SD). Two-tailed unpaired Student's *t*-test (**A**: $t_{(6)} = 6.025$, $p = 0.0038$). Two-sided Mann-Whitney test (**B**: $U = 0$, $p = 0.0022$). $^{**}p<0.01$. n.s. - no significant difference.

Figure S8

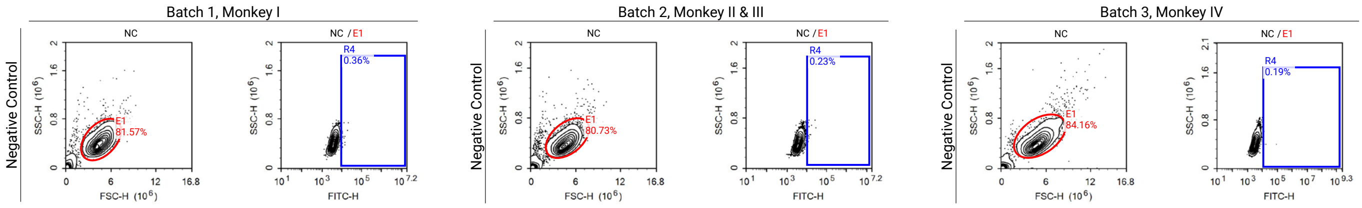

Batch 1, Monkey I
Batch 2, Monkey II & III
Batch 3, Monkey IV
Negative Control
NC
NC / E1
E1
81.57%
R4
0.36%
80.73%
0.23%
84.16%
0.19%
SSC-H (10^6)
FSC-H (10^6)
FITC-H

Figure S8. Gating strategy for FACS analysis. The gating strategy for quantifying GFP^{+} AAV2.WT-CMV-eGFP/FBS transduced HEK293T cells. The negative control HEK293T cells were incubated with only DMEM. For proper gating, FSC/SSC were used to exclude cell debris and cell aggregates and select target cell population E1 for analysis. In the FITC channel, GFP^{+} cells (R4) were identified by setting the boundary near the population of HEK293T cells not infected by AAV2.WT-CMV-eGFP, establishing a clear threshold to separate GFP^{+} cells from GFP^{-} cells.

Figure S9

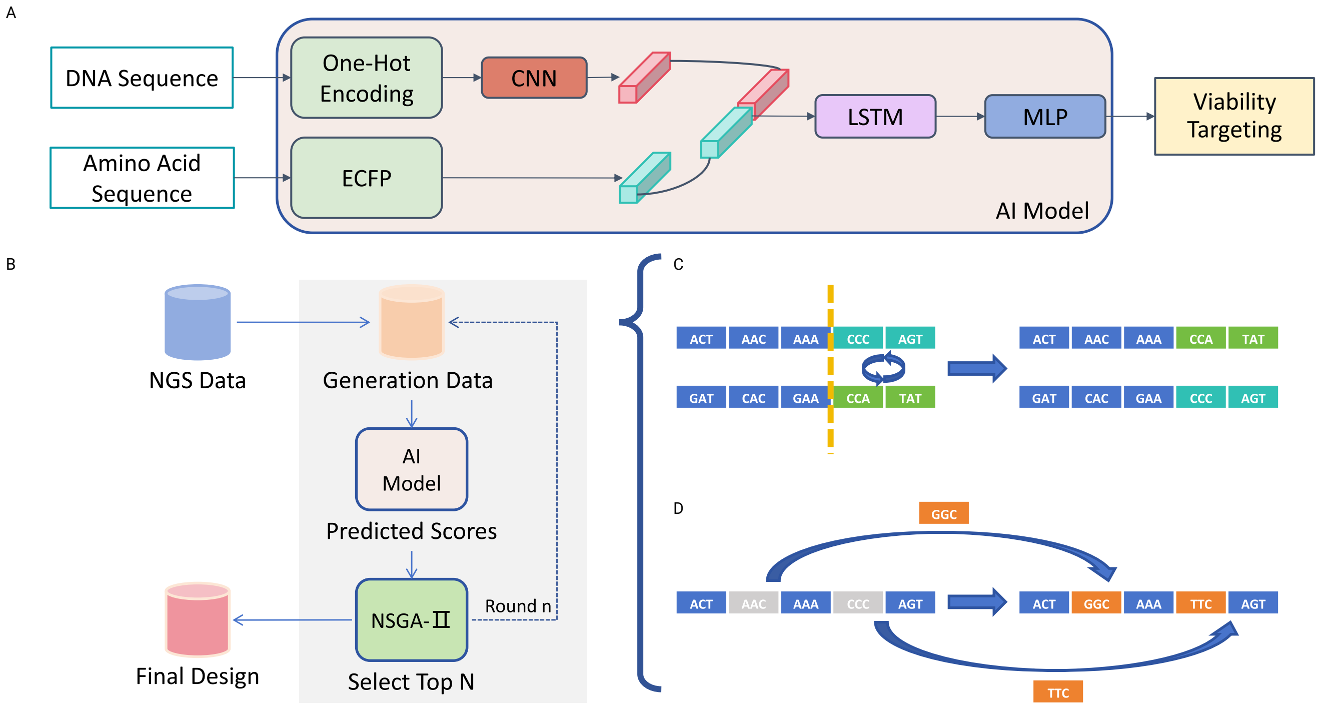
A
DNA Sequence
Amino Acid Sequence
One-Hot Encoding
CNN
ECFP
LSTM
MLP
AI Model
Viability Targeting
B
NGS Data
Generation Data
AI Model
Predicted Scores
NSGA-Ⅱ
Round n
Select Top N
Final Design
C
ACT AAC AAA CCC AGT
GAT CAC GAA CCA TAT
ACT AAC AAA CCA TAT
GAT CAC GAA CCC AGT
D
GGC
ACT AAC AAA CCC AGT
ACT GGC AAA TTC AGT
TTC

Figure S9. Visualized AI methodology. A, AI models for predicting AAV viability and targeting. **B**, Sequence generator. **C**, Fragment recombination. **D**, Base mutation.

Figure S10

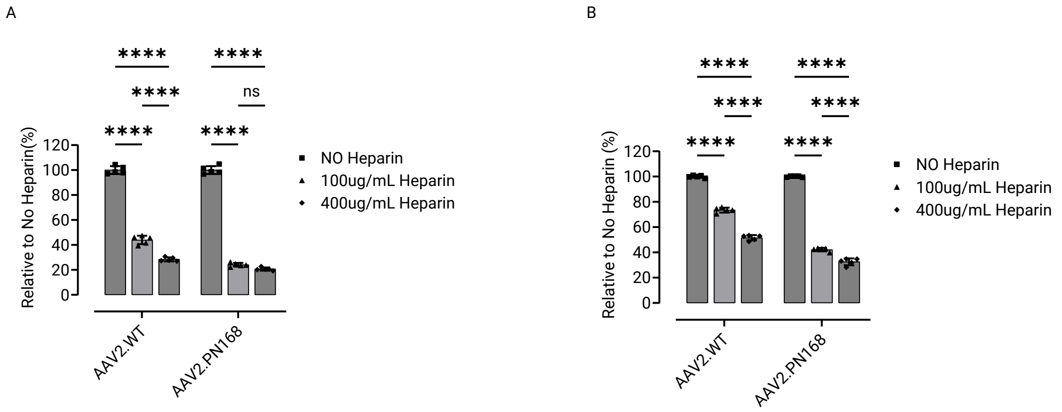
A
****
****
****
****
ns
****
Relative to No Heparin(%)
120
100
80
60
40
20
0
AAV2.WT
AAV2.PN168
NO Heparin
100ug/mL Heparin
400ug/mL Heparin
B
****
****
****
****
****
****
Relative to No Heparin (%)
120
100
80
60
40
20
0
AAV2.WT
AAV2.PN168
NO Heparin
100ug/mL Heparin
400ug/mL Heparin

Figure S10. Heparin competition assay. A, AAV-mediated mean fluorescent intensity upon administration of heparin sodium salt. **B**, AAV-transduced GFP positive cells upon administration of heparin sodium salt.
