# Supplementary material for: AI-engineered AAV capsid enables intravitreal delivery for the treatment of diverse retinal degenerations
Source: Mol Ther Adv. 2026 Jul 9;34(3):201806. doi: 10.1016/j.omta.2026.201806 (PMC13392946; doi:10.1016/j.omta.2026.201806)
Supplement: Document S2. Article plus supplemental information [file mmc2.pdf]

Molecular Therapy
Advances
Original Article

AI-engineered AAV capsid enables intravitreal delivery for the treatment of diverse retinal degenerations

Mochen Cui,[1,5] Huaqing Liu,[2,5] Lei Cai,[3,5] Qian Zhang,[1] Li Yuan,[1] Cui Gao,[1] Chunlian Li,[2] Jianfei Xi,[1] Yongkui Li,[4] Chenguang Wu,[1] Qin Zheng,[1] Lei Liu,[1] Peiyi Chen,[2] Shuxian Zhou,[2] Xing Mou,[2] Joseph Wekselblatt,[2] Yu Zhang,[3] Lance Han,[2] and Sheng Ren[1]

[1]YIMA Gene, Guangzhou, China; [2]Cyagen Biosciences, Guangzhou, China; [3]Guangdong Provincial Biotechnology Research Institute (Guangdong Provincial Laboratory Animals Monitoring Center), Guangzhou, China; [4]Institute of Medical Microbiology, Department of Immunology and Microbiology, College of Life Science and Technology, Jinan University, Guangzhou, China

Retinal degenerations, including Leber's congenital amaurosis type 1 (LCA1) and wet age-related macular degeneration (wAMD), represent leading causes of vision impairment and blindness, driven by a range of factors, including genetic mutations and pathological neovascularization. While adeno-associated viruses (AAVs) have emerged as a promising platform for sustained gene therapy, most AAVs typically require subretinal injections to reach target cells, which carry risks to retinal integrity. To overcome this limitation, we employed an AI-guided approach to AAV capsid engineering and developed AAV2.PN168, a novel AAV2-derived variant. AAV2.PN168 exhibits extensive retinal transduction via intravitreal injection in both non-human primates and mice. Furthermore, AAV2.PN168 demonstrates high therapeutic efficacy in mouse models both LCA1 and wAMD, respectively. These findings suggest that AAV2.PN168 has translational potential in treating various retinal degenerations, and other ocular diseases requiring efficient, wide-range retinal delivery of therapeutic agents.

INTRODUCTION

Retinal degeneration encompasses a group of diseases marked by the gradual deterioration of retinal structure and function, eventually resulting in vision impairment and, in severe instances, blindness. The onset of retinal degeneration can occur at various stages of life, depending on contributing factors such as genetic mutations or neovascularization.[1,2]

Despite the diverse causes of retinal degeneration, effective treatment typically requires sustained delivery of therapeutic agents to retinal photoreceptors and proximal structures. To this end, adeno-associated viruses (AAV), which enable prolonged gene expression in targeted cells[3] with low immunogenicity,[4] have therefore emerged as a promising platform (Figure 1). For example, in guanylate cyclase 2D gene (*GUCY2D*)-associated Leber's congenital amaurosis type 1 (LCA1), a hereditary retinal degeneration,[5,6] AAV is widely used in preclinical and clinical trials as vehicle to deliver a wild-type (WT) copy of the mutant gene directly to photoreceptors[7] (Figures S1A and S1B). Moreover, for retinopathies such as wet age-related macular degeneration (wAMD)—one of the most diagnosed eye conditions among individuals over 50[2]—traditional management relies on repeated intravitreal injections of antibodies against vascular endothelial growth factor A (VEGFA)[8,9] to suppress aberrant subretinal neovascularization and subsequent exudation[10,11] (Figures S2A and S2B). More recently, clinical trials have begun to explore sustained therapeutic strategies by using AAV to express VEGFA antibodies.[12–15]

Nevertheless, photoreceptors, as the outermost neuronal layer of the retina (Figures 1, S1, and S2), remain challenging to access from within the eye. Consequently, most AAV-mediated therapies for retinal degenerations are delivered via subretinal injections.[7,12–17] However, direct AAV delivery to photoreceptors and the adjacent retinal pigment epithelium (RPE) through subretinal injection disrupts these tightly connected cell layers, potentially leading to complications, such as maculopathy, retinal tears, subretinal deposits, worsening neovascularization, and even vision loss.[18] Additionally, subretinal injection only transduces cells within "bleb region" created by the procedure. Since degenerative processes usually affect multiple regions, there is a critical need to develop novel AAV capsids capable of pan-retinal or broad photoreceptor transduction

Received 14 August 2025; accepted 7 July 2026;
https://doi.org/10.1016/j.omta.2026.201806.

[5]These authors contributed equally

Correspondence: Yu Zhang, Guangdong Provincial Biotechnology Research Institute (Guangdong Provincial Laboratory Animals Monitoring Center), Guangzhou, China.
E-mail: zhangyu@gdlami.com
Correspondence: Lance Han, Cyagen Biosciences, Guangzhou, China.
E-mail: hanlance@cyagen.com
Correspondence: Dr. Sheng Ren, YIMA Gene, No. 288 Shenzhou Road, 510663 Guangzhou, China.
E-mail: shengren@cyagen.com

© 2026 The Author(s).
Published by Elsevier Inc. on behalf of The American Society of Gene and Cell Therapy.
This is an open access article under the CC BY license (http://creativecommons.org/licenses/by/4.0/).

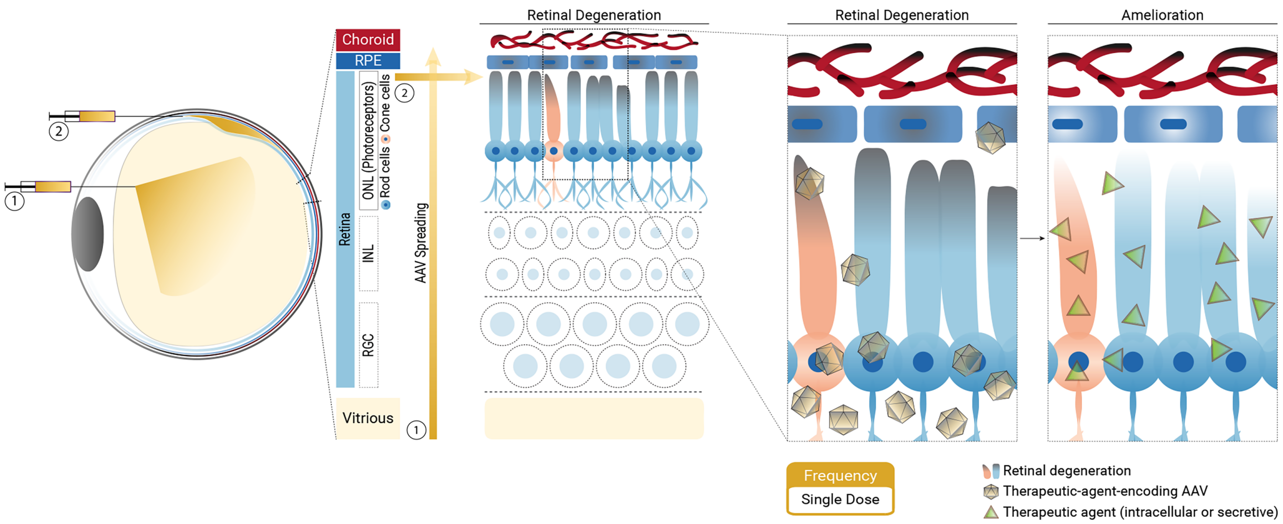

Figure 1. Schematic representation of AAV-mediated therapy for retinal degeneration
RGC, retinal ganglion cells; INL, inner nuclear layer; ONL, outer nuclear layer; RPE, retinal pigment epithelium.

using safer administration methods, such as intravitreal injection (Figure 1).

In recent years, various capsid engineering techniques have been developed. Besides traditional methods such as natural discovery, directed evolution or rational design of AAV capsids,[19–22] an increasing body of studies has adopted computational methods, such as artificial intelligence (AI) to more efficiently discover novel AAV capsids that are more advantageous in viability and organ-/tissue targeting.[23–30]

In response to this need, we have developed several AI models that focus on engineering novel variants of AAV2, the predominant AAV serotype for ocular-tissue targeting, to achieve efficient retinal penetration via intravitreal injection across multiple species. The resulting novel capsid, AAV2.PN168, exhibited robust retinal penetration and photoreceptor-targeting in both non-human primates (NHPs) and mice following intravitreal injections. Furthermore, AAV2.PN168-mediated expression of human *GUCY2D* and VEGFA antibody demonstrated sustained and high therapeutic efficacy in mouse models of LCA1 and wAMD, respectively. Collectively, our results highlight the significant clinical relevance of AI-engineered AAV2.PN168 for AAV-mediated therapies in various forms of retinal degeneration, as well as in other ocular diseases requiring rapid and efficient retinal expression of therapeutic genes.

RESULTS

Development of AI-guided approach for AAV capsid engineering

Recent advancements in next-generation sequencing (NGS)-based assays have enabled large-scale data acquisition suitable for training AI models, which can predict novel sequences with specific properties.[28] Based on this, we developed an AI-guided approach to obtain AAV2-derived variants with improved viability and retina targeting. We first generated an AAV2-derived capsid library. Previous studies have indicated that components of the extracellular matrix, such as heparan sulfate proteoglycan (HSPG), serve as primary mediators during the initial cell attachment of AAV2.[31,32] Additionally, recent research has demonstrated enhanced cell tropism of AAV2-derived variants with modified heparin-binding domains.[21] Building upon this, we focused on mutagenesis of the AAV2 capsid protein (Cap) with random sequences of 7–12 amino acids (7-mer–12-mer) inserted into its heparin-binding domain, specifically between the viral protein 3 (VP3) residues $R_{587/588}$[33] (Figure 2A). To this end, we created an oligo pool with a diversity of 10^8 based on the NNK principle (where N represents any base and K represents G or T), integrated them into inverted-terminal-repeat (ITR)-containing *Cap* plasmid, and subsequently vectorized the plasmids into a library of AAV variants respectively packaging their *Cap* genes. We assessed the viability of each AAV variant by performing *Cap*-specific DNA amplicon sequencing on both the plasmid pool and the AAV library (Figures 2A and S9). The viability of each AAV variant was scored by normalizing its reads per million (RPM) in the AAV library to the RPM of the corresponding plasmid in the plasmid pool.

To capture and predict variations in the AAV viability score, we developed a regression AI model (viability model) as demonstrated in the prior study.[28] The model was trained using the *Cap* sequences and their corresponding viability scores from approximately 60% of the initial AAV library (Figure 2B), while the remaining portion was designated as the test dataset. The viability model consistently demonstrated high prediction accuracy across test datasets (Spearman R = 0.852, Figure 2B), indicating robust training.

To create new AAV variants with high viability, we designed a genetic algorithm-based sequence generator incorporating base-mutation and fragment-recombination techniques, integrated with the viability

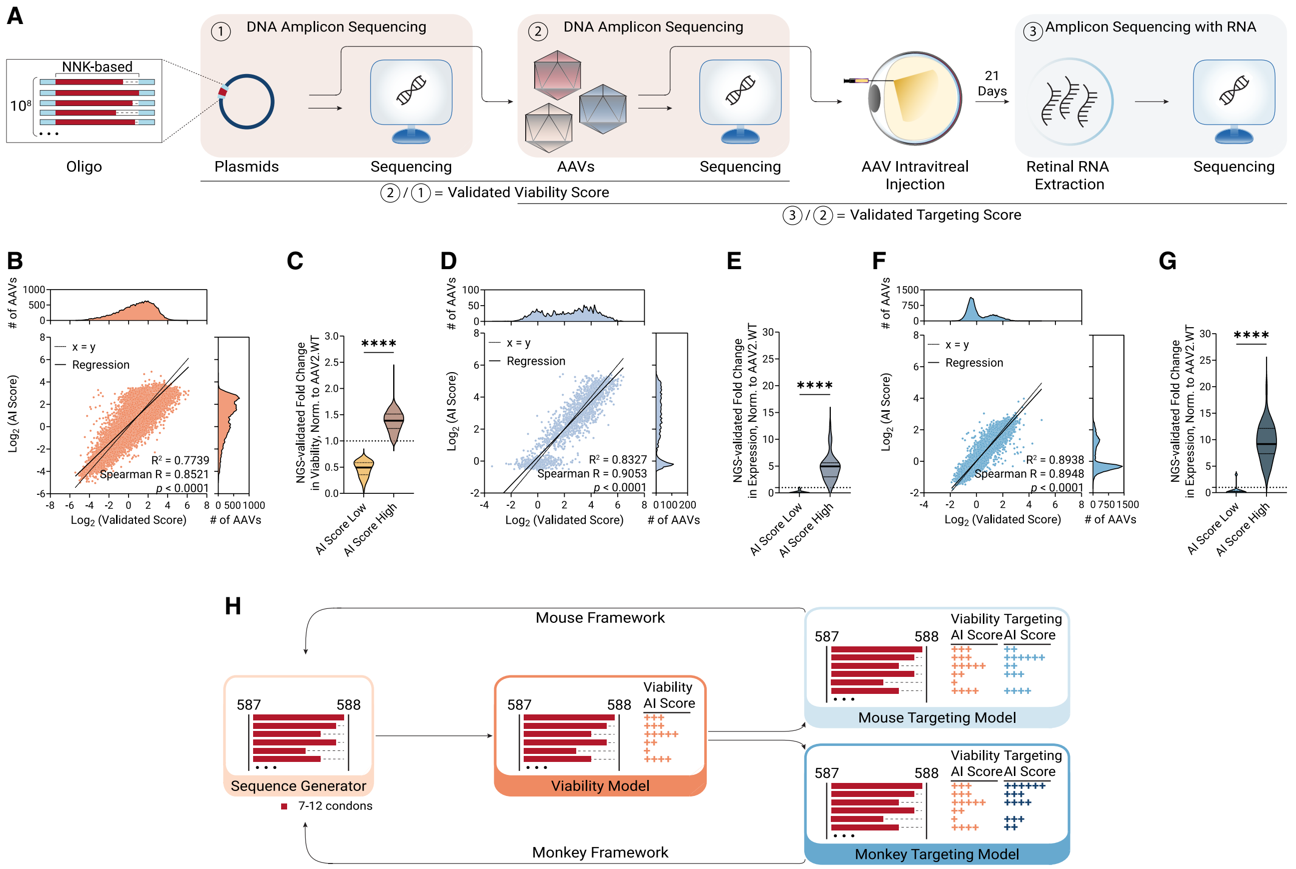

Figure 2. AI-guided approach of AAV engineering

(A) The validation process for AI-predicted viability and targeting scores. (B) The correlation between the AI-predicted viability score and validated viability score of AAVs from the initial AAV library. The vertical and horizontal marginal charts correspond to the distribution of the sequences of different scores. (C) The validated viability of representative AAVs designed by AI, using DNA amplicon sequencing. (D) The correlation between the AI-predicted targeting score and validated targeting score of AAVs from the initial AAV library in mouse. The vertical and horizontal marginal charts correspond to the distribution of the sequences of different scores. (E) The validated targeting performance in mouse of representative AAVs designed by AI, using DNA amplicon sequencing. (F) The correlation between the AI-predicted targeting score and validated targeting score of AAVs from the initial AAV library in monkey. The vertical and horizontal marginal charts correspond to the distribution of the sequences of different scores. (G) The validated targeting performance in monkey of representative AAVs designed by AI, using DNA amplicon sequencing. (H) Schematic representations of mouse and monkey frameworks. $n = 22{,}636$ AAVs (B); $n = 227$ AAVs (C, low), $n = 4.867$ AAVs (C, high); $n = 1{,}904$ AAVs (D); $n = 11$ AAVs (E, low), $n = 39$ AAVs (E, high); $n = 19{,}285$ AAVs (F); $n = 15$ AAVs (G, low), $n = 37$ AAVs (G, high). For violin plots, median values and interquartile ranges are plotted. Two-sided Mann-Whitney test (C: $U = 0$, $p < 0.0001$; E: $U = 0.5$, $p < 0.0001$; G: $U = 0$, $p < 0.0001$). ****$p < 0.0001$.

model. Together, they form a cyclic iterative design framework capable of generating sequences of predetermined lengths, followed by automatic AI-guided scoring. Sequences with an AI-predicted viability score surpassing the threshold are fed back into the generator for continuous iterative design until enough high-viable sequences are generated. The representative AI-generated sequences are subsequently validated using DNA amplicon sequencing (Figure 2C).

Subsequently, we developed another NGS-data-based regression AI model aimed at capturing and predicting the retina-targeting capabilities of AAV2-derived variants, specifically for mice (mouse targeting model). To achieve this, the initial AAV library, analyzed through *Cap*-specific DNA amplicon sequencing, was intravitreally injected into mouse eyes. Twenty-one days post-injection, the eyes were extracted, the retinas isolated, and subjected to *Cap*-specific amplicon sequencing using extracted mRNA (Figure 2A). With all AAV variants being qualitatively and quantitatively tracked by NGS, both before and after the injection (AAVs with RPM >0.25 are included), their normalized RPM (pre-to post-injection) was calculated as their individual-targeting scores and used for training and testing the-targeting model (Figures 2A and 2H). The-targeting model consistently exhibited high prediction accuracy across test datasets (Spearman R = 0.905, Figure 2D), indicating robust training. The resulting sequences were also vectorized and injected into mice, with their targeting scores validated by NGS (Figure 2E).

To further enhance the translational potential of the AI-guided approach for AAV engineering, we adopted the targeting model

Table 1. Transduction inhibition rate of monkey serum on AAV2.WT

		Transduction inhibition rate (%)				
Monkey	Eye	sample 1	sample 2	average	Procedure	Related Figure
I	1	−8.994	−5.323	−7.158	AAV library for AI model training	Figures 2F and 2G
	2					
II	1	−4.129	1.885	−1.122	45× AAV2.variant-CAG-barcoded eGFP, 3E11 vg/eye	Figures 3A–3C
III	1	−2.515	0.236	−1.140	AAV2.7m8-CAG-eGFP, 5E10 vg/eye	Figure 3E
	2				AAV2.PN168-CAG-eGFP, 5E10 vg/eye	Figure 3E
IV	1	1.955	2.843	2.399	AAV2.PN168-CAG-eGFP, 5E11 vg/eye	Figure S5G

To evaluate the transduction inhibition rate of AAV2 neutralizing antibodies derived from monkey serum. Two serum samples from each monkey were tested. The table provides the AAV2 transduction inhibition rate across two individual trials, and the calculated average transduction inhibition rate.

establishment from mice to NHPs. In this study, cynomolgus monkeys were used, and baseline blood tests were conducted on all monkeys before AAV injection. To evaluate the presence of AAV2-neutralizing antibodies, monkey serum was incubated with enhanced green fluorescent protein (eGFP)-expressing AAV2, and the mixture was subsequently applied to HEK293T cells. The strong detection of GFP-positive cells (indicating successful transduction) (Figures S3A–S3C and S8) demonstrated that AAV2-neutralizing antibodies were either absent or insignificant. Therefore, only monkeys that tested negative for these antibodies were selected for further experiments. The NGS-analyzed initial AAV library was intravitreally injected into the eyes of AAV2-neutralizing-antibody-free cynomolgus monkeys that had been screened for suitable eye conditions (Table 1; Figures S3A and S8), and waited for 21 days. After the waiting period, the monkey retinas were harvested and subjected to amplicon sequencing using extracted mRNA, with the readouts (AAVs with RPM >0.25 are included, normalization was performed as in the mouse targeting model) utilized to train and test the NHP-targeting model (Figures 2A and 2H). As illustrated in Figure 2F, the high prediction accuracy (Spearman R = 0.895, Figure 2F) of the model reflected sufficient training robustness. The new sequences resulting from this process were validated using the same methods applied to the mouse targeting model (Figure 2G).

The two targeting models were then integrated into the aforementioned iterative design framework. These new tripartite frameworks (i.e., mouse or monkey framework), allow the assessment of AAV variants from the sequence generator for their viability and species-specific retina-targeting capability (Figures 2H, S4A, and S4B). Eligible variants are continuously reintroduced into the sequence generator to produce additional sequences with similar or improved AI-predicted scores for these properties.

In summary, we developed two AI-guided tripartite iterative design frameworks for AAV engineering. For either mice or monkeys, these design frameworks generate AAV2-derived variants that are potentially viable and capable of effectively transducing retinal tissue following intravitreal injections.

AI-predicted AAV2.PN168 drives a broad protein expression across the monkey retina

Using the established monkey framework, we developed a small-scale library comprising 43 AI-designed AAV2-derived variants, with highest cumulative predicted scores from viability (30% weight) and retina targeting (70% weight) (Figures S5A and S5B). For comparison purposes, the library also included AAV2.WT and AAV2.7m8,[21] a capsid recognized for its retina-targeting capability and used in multiple clinical trials, typically administered intravitreally.[21] All 45 variants were designed to carry genes encoding barcoded eGFP, which serve as identifiers for the subsequent NGS detection (Figure 3A).

Subsequently, the library was administered intravitreally into the eyes of eligible monkeys (Table 1; Table S2; Figures 3A, S3B, and S8). After a 21-day incubation period, the retinas were harvested and subjected to single-cell mRNA sequencing (scRNA-seq). Consistent with the predictions of the AI model, most of the AI-designed variants exhibited higher transduction efficacy in major cell types, with photoreceptors (rod cells and cone cells) being the primary transduced cells in monkey retinal tissue compared to AAV2.WT (Figures 3B, 3C, and S5L). On top of this, from a small subset of the AI-designed variants performed similarly or better than AAV2.7m8, AAV2.PN168 (production after purification: 2.74× higher relative to AAV2.WT, as shown in Figure S5C; insertion sequence: ^{588}AAVRFDGTERAA) emerged as the optimal variant, demonstrating exceptionally high retinal transduction efficacy (Figures 3B and 3C).

To evaluate the performance of AAV2.PN168 individually, we synthesized (Figures S5C–S5E) and intravitreally administered AAV2.PN168-CAG-eGFP and AAV2.7m8-CAG-eGFP into respective monkey eyes at a concentration of 5E10/eye (Figure 3D; Table 1). After the 21-day incubation period, both retinas were harvested and cross-sectioned. Within the macula-containing sectional planes of the retina, the expression of the GFP driven by AAV2.PN168 covers a significantly broader retinal range compared to AAV2.7m8 (Figures 3E and 3F). Furthermore, we quantified the percentage of GF*P*-expressing cells within the retinal outer nuclear

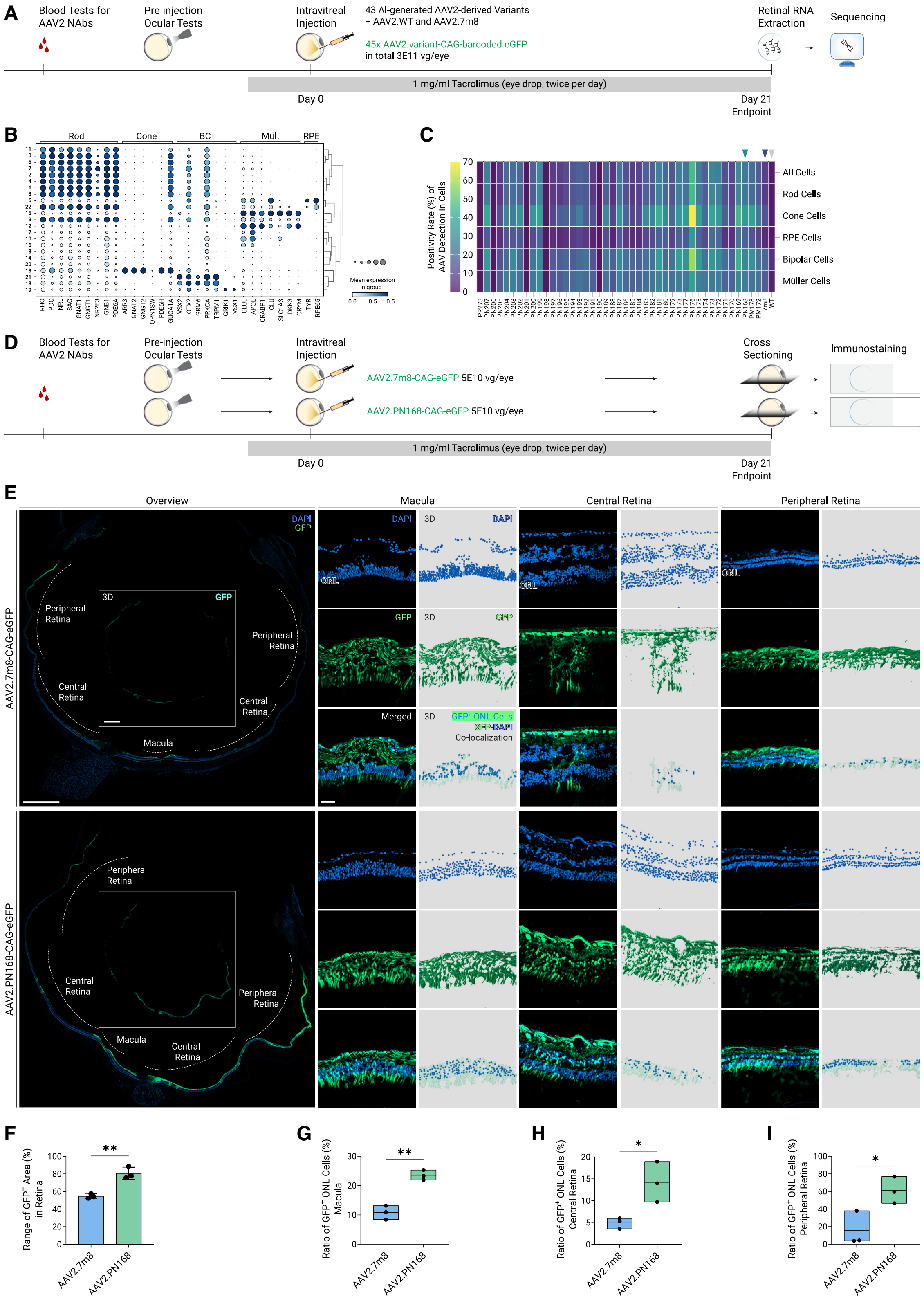

(legend on next page)

layer (ONL), which mainly contains photoreceptors, at different regions (macula, central retina, and peripheral retina) using Imaris-based 3D reconstruction and analysis. Our data revealed that AAV2.PN168-driven GFP expression was detected in a higher proportion of ONL cells in all regions compared to AAV2.7m8 (Figures 3G–3I), consistent with scRNA-seq data showing AAV2.PN168's photoreceptor-dominant expression. Moreover, higher doses of AAV2.PN168-CAG-eGFP resulted in even broader GFP expression and increased ONL cell transduction (Table 1; Figures S3C, S5F, S5G, and S8). Importantly, fundoscopic examination showed no visible inflammation following AAV2.PN168 administration (Figures S5H and S5I). Additionally, analysis of immune-associated factors and pathways in scRNA-seq data from the library-injected monkey eye revealed no significant differences between cells with and without AAV transduction (Figures S5J and S5K), aligning with previous studies.[34]

Our findings suggest that AAV2.PN168, an AI-designed AAV2 variant, demonstrates exceptional efficacy in transducing multiple cell types of the monkey retina. Remarkably, this AAV drives significant protein expression within the ONL, primarily composed of photoreceptors that are essential for visual functionality.

AAV2.PN168 drives a broad protein expression across the mouse retina

Having identified AAV2.PN168 as a novel AAV2 derivative with high viability and transduction efficacy in the monkey retina, we next sought to evaluate its therapeutic potential for treating retinal degenerations such as LCA1 and wAMD. However, technical limitations restrained the development of transgenic simian models for these conditions, particularly those involving human *GUCY2D* deficiency and *VEGFA* transgenesis. Consequently, we decided to validate the therapeutic capability of AAV2.PN168 for LCA1 and wAMD in mice, where the generation of genetically modified strains is more feasible.

To determine if the high retina transduction of AAV2.PN168 seen in monkey could be translated to mice, we examined its transduction efficacy in the retinal tissue of WT mice. Similar to the monkey experiments, we assembled a small-scale library, containing AAV2.WT, AAV2.7m8, AAV2.PN168, and 47 AAV2.variants designed by the mouse framework (Table S3). All these viruses carried genes encoding barcoded eGFP. Following a 21-day incubation period after the intravitreal injection into the mouse eyes, the retinal tissues were harvested for scRNA-seq (Figure 4A). Although not the top performer, AAV2.PN168 demonstrated greater transduction efficacy in the mouse retina compared to AAV2.WT and AAV2.7m8 (Figures 4B, 4C, and S5M).

To further validate the NGS results, we employed AAV2.WT, AAV2.7m8, and AAV2.PN168, along with some AAV2-derived variants from the NGS, to encapsulate the sequence encoding luciferase (AAV2.variant-CAG-LUC) (Figures 4D and S6). Luciferase is activatable via intraperitoneal (I.P.) injection of D-luciferin, resulting in luminescence suitable for *in vivo* detection. Following intravitreal injections and a 21-day incubation period, upon D-luciferin administration, mice received AAV2.PN168-CAG-LUC exhibited increased luminescence in their eyes compared to those receiving AAV2.WT (Figures 4E and 4F), indicating enhanced transduction efficacy of AAV2.PN168 in facilitating protein expression in mouse eyes. In contrast, the performance of AAV2.7m8 was closer to AAV2.WT (Figures 4E and 4F), consistent with the data from previous studies.[35]

In alignment with the monkey experiments, the protein distribution in the mouse retina induced by AAV2.PN168 was assessed using AAV2.PN168-CAG-eGFP, in comparison to AAV2.7m8-CAG-eGFP and AAV2.WT-CAG-eGFP at the same dose (Figure 4G). Following intravitreal injection and a 21-day incubation period, fluorescence expression was quantified through both *in vivo* fundoscopy in living mice and microscopy using retinal cross sections. The fundoscopic examination indicated a higher GFP signal in mice injected with AAV2.PN168 compared to other groups (Figures 4H and 4I). Consistently, assessment results of the mouse retinal sections showed that AAV2.PN168 produced a more intense pan-retinal GFP expression than AAV2.WT and AAV2.7m8 (Figures 4H and 4I). In addition, droplet digital PCR (ddPCR) analysis of retinal tissue from AAV-injected mice confirmed these findings, further supporting the superior transduction achieved by AAV2.PN168 over other AAV2 derivatives (Figure 4K).

Overall, our results demonstrate that AAV2.PN168—though not the best-performing AI-generated AAV2 variant for mice—achieves substantially greater retinal transduction efficacy than AAV2.WT following intravitreal administration. These results support its utility as a gene therapy vector for investigating capsid-associated therapeutic efficacy in LCA1 and wAMD mouse models.

Figure 3. AAV2.PN168 exhibits a high transduction rate and facilitates extensive protein expression throughout the monkey retina

(A) Experimental setup. (B) Bubble plot displaying the expression of marker genes used to identify the major retinal cell types in monkeys (Rod, rod cells; Cone, cone cells; BC, bipolar cells; Mül., Müller cells; RPE, retinal pigment epithelium cells). (C) Positivity rate of detection for different AAV variants in various retinal cell types, with results of AAV2.WT, AAV2.7m8, and AAV2.PN168 indicated by the pointers. (D) Experimental setup. (E) Representative confocal micrographs and 3D reconstruction of total retinal cross sections and different retinal regions from monkey eyes receiving different GFP-expressing AAVs. ONL is indicated in the graphs. (F) Quantification of GFP positive range relative to the entire range of retina. (G) Quantification of the portion of GFP-positive ONL cells in macula. (H) Quantification of the portion of GFP-positive ONL cells in central retina. (I) Quantification of the portion of GFP-positive ONL cells in peripheral retina. 7,742 retinal cells were analyzed (B and C). $n = 1$ eye (for either AAV2.PN168 or AAV2.7m8), 3 non-consecutive sections (interval >10 μm) within the macula-containing sectional planes were imaged and analyzed (E–I); Dot plots show mean (SD). Floating bars: line represents mean value. Two-tailed unpaired Student's *t* test (F: $t_{(6)} = 6.025$, $p = 0.0038$; G: $t_{(6)} = 6.108$, $p = 0.017$; H: $t_{(6)} = 3.171$, $p = 0.0248$; I: $t_{(6)} = 2.933$, $p = 0.0325$). $^*p < 0.05$, $^{**}p < 0.01$. n.s. - no significant difference. Scale bars: (E), 2,000 μm (overview), 50 μm (individual retinal regions).

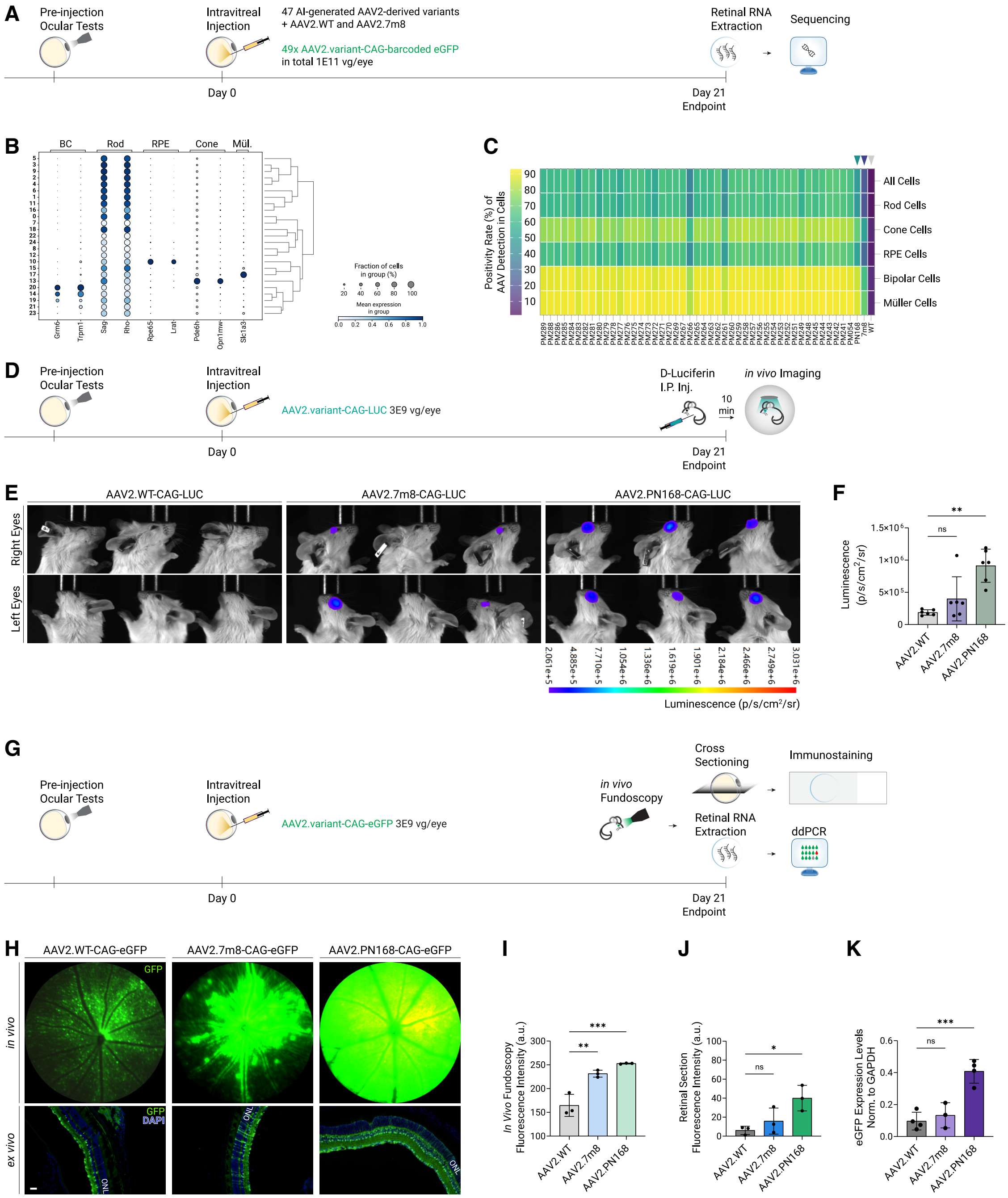

Figure 4. AAV2.PN168 exhibits a high transduction rate and facilitates extensive protein expression throughout the mouse retina
(A) Experimental setup. (B) Bubble plot displaying the expression of marker genes used to identify the major retinal cell types in mice (Rod, rod cells; Cone, cone cells; BC, bipolar cells; Mül., Müller cells; RPE, retinal pigment epithelium cells). (C) Positivity rate of detection for different AAV variants in various retinal cell types, with results of AAV2.WT, AAV2.7m8, AAV2.PN168 indicated by the pointers. (D) Experimental setup. (E) *In vivo* imaging following D-luciferin injection into mice administered with different luciferase-expressing AAVs. (F) Quantification of luminescence from the ocular region upon D-luciferin administration. (G) Experimental setup. (H) Micrographs of *in vivo* fundoscopy (square) and retinal cross sections (rectangle) from mouse eyes treated with different GFP-expressing AAVs. ONL is indicated in the graphs. (I and J) Quantification of fluorescence intensity from micrographs acquired via *in vivo* fundoscopy (I) and retina cross sections (J). (K) Quantification of *eGFP* mRNA expression, normalized to *Gapdh* mRNA. 18,577 retinal cells were analyzed (B and C). $n = 6$ eyes (2 eyes/mouse, 3 mice/group) (E and F); $n = 3$ eyes (1 eye (R)/mouse, 3 mice/group) (H–J); n = 3–4

(legend continued on next page)

AAV2.PN168 mediates significant therapeutic efficacy in a mouse model of LCA1

To model LCA1 in mice, we genetically depleted both *Gucy2e*, the murine ortholog of human *GUCY2D*, and its paralog *Gucy2f*. This approach ensures a complete absence of guanylate cyclase 1 and 2 in mouse retinal photoreceptors, leading to a strong LCA1 phenotype.[36]

In consistent with human LCA1 pathophysiology, we observed significant reduction in ONL thickness at 1 month of age in Gucy2e/Gucy2f double-knockout (*GucyDKO*) mice, as determined by optical coherence tomography (OCT) (Figures 5A and 5B). This finding indicates severe photoreceptor degeneration in this model.

As demonstrated previously, introduction of *hGUCY2D* can ameliorate the LCA1 pathology upon absence of Gucy2e and Gucy2f genes.[7] Accordingly, we constructed AAV2.PN168- (or AAV2.7m8-) hGRK1-hGUCY2D and administered them intravitreally into the eyes of *GucyDKO*, utilizing the hGRK1 promoter for photoreceptor-specific expression.[36–39] As shown in Figure 5A, 28 days after AAV (or PBS) injection, mice treated with AAV2.PN168-hGRK1-hGUCY2D showed the greatest increase in electroretinography (ERG) amplitude under both dark (scotopic) and light (photopic) conditions, surpassing those treated with vehicle or AAV2.7m8-hGRK1-hGUCY2D, indicating the superior recovery of visual functionality (Figure 5C). Furthermore, ERG reevaluation at 56 days post-AAV-injection confirmed the sustained therapeutic effect of AAV2.PN168-mediated *hGUCY2D* expression (Figure 5D). In line with these findings, we evaluated behavioral outcomes using the optomotor response (OMR) test, which similarly demonstrated that AAV2.PN168-hGRK1-hGUCY2D treatment led to the highest level of recovered response time across all groups (Figure 5E).

To corroborate our functional assessments, we measured the AAV-mediated *hGUCY2D* expression using ddPCR. As shown in Figure 5F, retinal tissue from mice treated with AAV2.PN168-hGRK1-hGUCY2D exhibited the highest levels of *hGUCY2D* mRNA expression.

Collectively, in *GucyDKO* mice modeling human LCA1, AAV2.PN168 efficiently mediates robust expression of *hGUCY2D*, resulting in significant and sustained functional recovery of the retinal visual function.

AAV2.PN168 mediates significant therapeutic efficacy in a mouse model of wAMD

To assess the therapeutic potential of AAV2.PN168, we established a mouse model of wAMD—the *hVEGFA* transgenic strain (*hVEGFA-TG*)—in which *hVEGFA*, a major contributor to wAMD, is overexpressed in mouse retina under the bovine rhodopsin promoter[40,41] (Figures S7A and S7B).

We first examined disease-related phenotypes in *hVEGFA-TG* mice, focusing on the subretinal exudation caused by abnormal neovascularization. As illustrated in Figures S7C and S7D, following the I.P. injection of fluorescein sodium (1% AK-FLUOR solution), multiple fluorescent spots were observed during angiography via *in vivo* fundoscopy in the eyes of the transgenic mice, but not in the WT mice, indicating the occurrence of subretinal exudation[10,11]—a pathological hallmark and a principal cause of retinal disruption in wAMD—within the *hVEGFA-TG* mice.

To address dysregulated VEGFA in wAMD, one of the most widely accepted treatments involves the use of anti-VEGFA antibodies such as ranibizumab. To evaluate the therapeutic potential of AAV2.PN168 for wAMD, this vector was employed alongside AAV2.7m8 and AAV2.WT to package the sequence encoding the fragment of ranibizumab (AAV2.variant-CAG-anti-VEGFA) as described previously.[42] As depicted in Figure 6A, ranibizumab and the AAVs were administered via intravitreal injection into mouse eyes following their respective protocols over a 21-day treatment period. Compared to *hVEGFA-TG* mice treated with AAV2.WT and AAV2.7m8, AAV2.PN168 demonstrated a higher expression of mRNA for the anti-VEGFA antibody in retinal tissue by the end of the treatment (Figures 6B and 6C). Corroborating this observation, while both ranibizumab and AAV-mediated VEGFA antibodies showed promising efficacy in attenuating subretinal exudation in *hVEGFA-TG* mice, based on the *in vivo* fundoscopy over the 21-days period, the therapeutic effect mediated by AAV2.PN168 was swifter and more pronounced compared to both ranibizumab and other AAV2 variants (Figures 5D–5F). Additionally, it should be noted that AAVs require only a single injection, unlike the weekly administration required for ranibizumab, thereby significantly reducing the risks associated with repeated intravitreal injections.

Collectively, these findings underscore the superior efficacy of AAV2.PN168 in treating wAMD pathology by facilitating the expression of anti-VEGFA antibody. Furthermore, due to the stability and prolonged duration of AAV-driven gene expression, AAV2.PN168 provides substantial advantages in terms of safety and patient compliance when transitioned into clinical practice.

DISCUSSION

In this study, we established an AI-guided strategy for AAV engineering that led to the development of the novel capsid AAV2.PN168. We subsequently demonstrated that AAV2.PN168 achieves high production yield and broad retinal transduction following intravitreal injection in both monkeys and mice.

eyes (1 eye (R)/mouse, 3–4 mice/group) (K). Dot plots show mean (SD). Kruskal-Wallis test (F: $K = 10.4$, $p = 0.0016$). One-way ANOVA followed by Dunnett's multiple comparisons test (I: $F_{(2, 6)} = 7.033$, $p = 0.0267$; J: $F_{(2, 9)} = 31.78$, $p = 0.0006$; K: $F_{(2, 8)} = 23.61$, $p = 0.0004$). *$p < 0.05$, **$p < 0.01$, ***$p < 0.001$. n.s., no significant difference. Scale bars: (H), 50 μm. For fundus images, all images were obtained using a fundus camera at a fixed magnification and focal length, ensuring comparable coverage and locations across different groups, no scale was indicated by the imaging system.

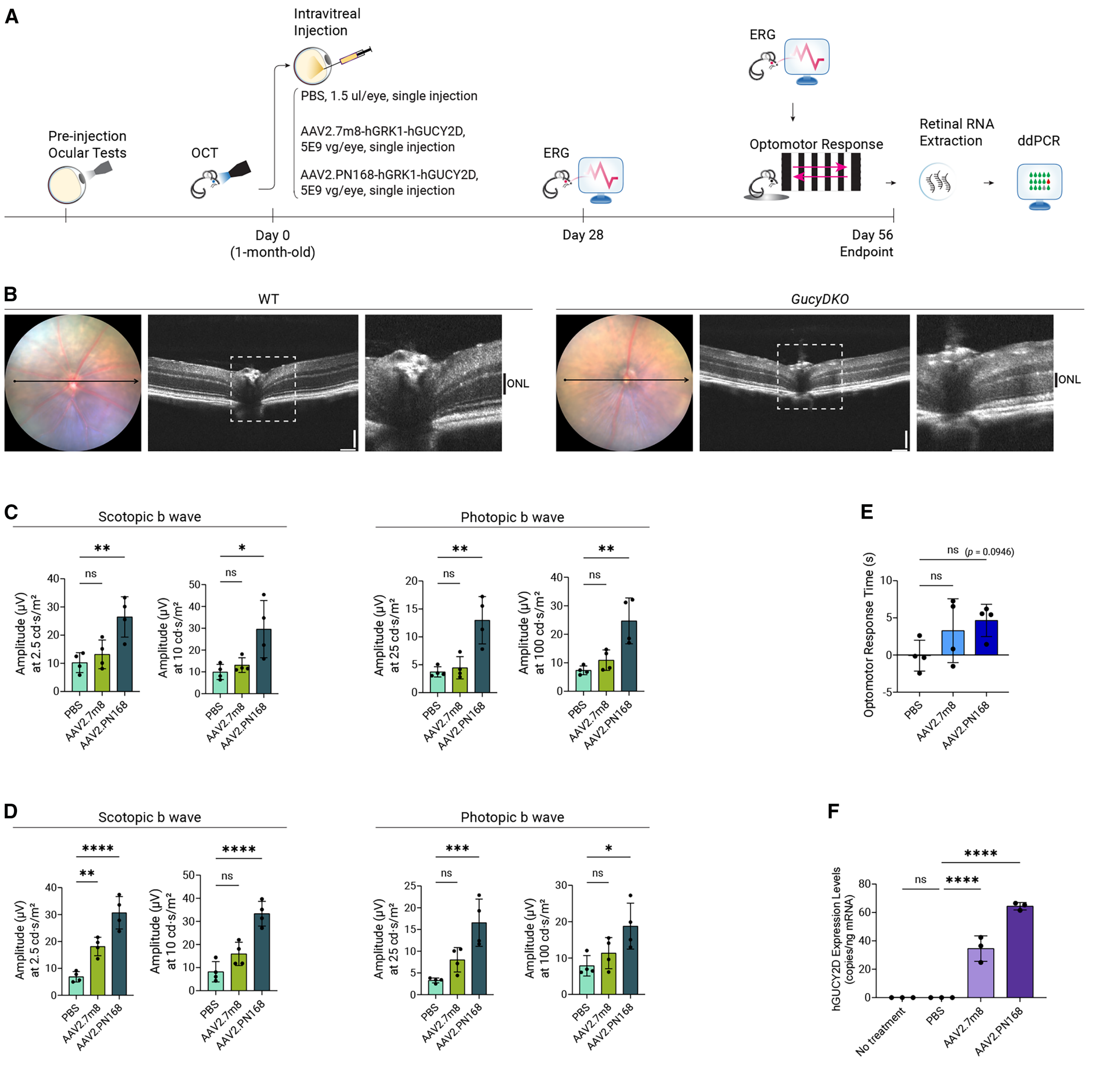

Figure 5. AAV2.PN168 effectively mediates *hGUCY2D*-associated therapy in an LCA1 mouse model

(A) Experimental setup. (B) Retinal OCT from WT and *GucyDKO* mice at 1 month old, the arrow indicates the path of the OCT scan, and the area within the dashed square is enlarged to highlight the thickness of the ONL. (C and D) ERG b-wave amplitudes (scotopic/photopic) quantified in GucyDKO mice treated with PBS, AAV2.7m8, or AAV2.PN168 at 28 (C) and 56 (D) days post-injection. (D) Quantification of ERG-recorded amplitude of scotopic and photopic b wave from *GucyDKO* mice treated with different compounds, 56 days after injection. (E) Optomotor response time of *GucyDKO* mice treated with different compounds, measured at 56 days after treatment. (F) *hGUCY2D* mRNA expression in retina from *GucyDKO* mice treated with different compounds at 56 days post-treatment. $n = 4$ eyes, 1 eye (R)/mouse, 4 mice/group (C–E); $n = 3$ eyes, 1 eye (R)/mouse, 3 mice/group (F). Dot plots show mean (SD). One-way ANOVA followed by Dunnett's multiple comparisons test (C: scotopic (2.5): $F_{(2, 9)} = 9.982$, $p = 0.0052$, scotopic (10): $F_{(2, 9)} = 6.852$, $p = 0.0155$, photopic (25): $F_{(2, 9)} = 13.91$, $p = 0.0018$, photopic (100): $F_{(2, 9)} = 12.65$, $p = 0.0024$; D: scotopic (2.5): $F_{(2, 9)} = 32.65$, $p < 0.0001$, scotopic (10): $F_{(2, 9)} = 27.12$, $p = 0.0002$, photopic (25): $F_{(2, 9)} = 14.32$, $p = 0.0016$, photopic (100): $F_{(2, 9)} = 5.624$, $p = 0.0260$; E: $F_{(2, 9)} = 2.591$, $p = 0.1292$; F: $F_{(3, 8)} = 132.4$, $p < 0.0001$). *$p < 0.05$, **$p < 0.01$, ***$p < 0.001$, ****$p < 0.0001$. n.s., no significant difference. Scale bars: (B), 100 μm. For fundus images, all images were obtained using a fundus camera at a fixed magnification and focal length, ensuring comparable coverage and locations across different groups, no scale was indicated by the imaging system.

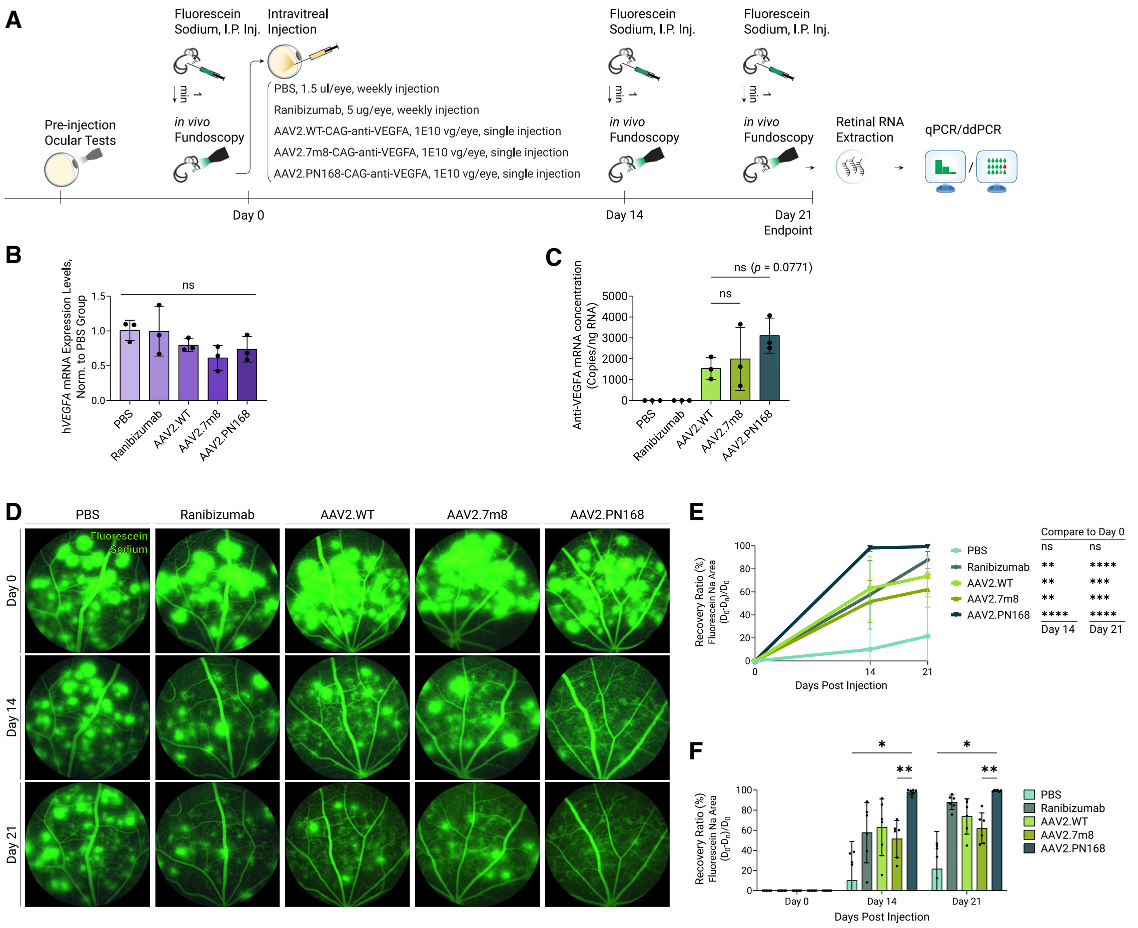

Figure 6. AAV2.PN168 effectively mediates anti-VEGFA therapy in a wAMD mouse model

(A) Experimental setup. (B) qPCR for mRNA levels of *hVEGFA* in retinal tissue of *hVEGFA-TG* mice, normalized to PBS group. (C) ddPCR for mRNA concentrations of anti-VEGFA antibody. (D) Representative micrographs obtained via *in vivo* fundoscopy upon fluorescein sodium injection. (E and F) Quantification of recovery ratio of subretinal exudation at different time points following the initial vehicle/ranibizumab/AAVs injection a long time (E) and cross groups (F). *n* = 3 eyes, 1 eye (R)/mouse, 3 mice/group (B and C); *n* = 6 eyes, 1 eye (R)/mouse, 6 mice/group (E and F). Dot plots show mean (SD). One-way ANOVA followed by Dunnett's multiple comparisons test (B: $F_{(4,\ 10)} = 1.964$, $p = 0.1763$). One-way ANOVA followed by Bonferroni's multiple comparisons test (C: $F_{(4,\ 10)} = 8.243$, $p = 0.0033$). Repeated measures ANOVA with Geisser-Greenhouse correction followed by Dunnett's multiple comparisons test (E: $F_{\text{interaction: time × treatment } (8,\ 50)} = 7.707$, $p < 0.0001$). Two-way ANOVA with Geisser-Greenhouse correction followed by Turkey's multiple comparisons test (F: $F_{\text{interaction: time × treatment } (8,\ 50)} = 7.707$, $p < 0.0001$). *$p < 0.05$, **$p < 0.01$, ***$p < 0.001$, ****$p < 0.0001$. n.s., no significant difference. For fundus images, all images were obtained using a fundus camera at a fixed magnification and focal length, ensuring comparable coverage and locations across different groups, no scale was indicated by the imaging system.

Furthermore, in mouse models of diverse retinal degenerations—including LCA1 and wAMD—we observed that AAV2.PN168 mediates significantly greater therapeutic effect when delivering *hGUCY2D* and anti-VEGFA antibody sequences, respectively, compared to delivery via alternative AAV2 derivatives. This improvement is attributable to enhanced expression of the packaged sequences, resulting in better mitigation of the corresponding retinal degenerative pathologies.

This advance is particularly significant in clinical practice for treating LCA1. As mentioned before, to date, most clinical trials for LCA1 utilize subretinal route for therapeutic agent administration.[7,16] This approach by nature is a compromise, driven by the anatomical position of the ONL within the retina and the delivery limitations of existing methods. Here, we demonstrate the potency of AAV2.PN168-mediated expression of *hGUCY2D* and the notable visual recovery after intravitreal injection, supporting the use of AAV2.PN168 as a safer option in future clinical trials for LCA1 and other ocular conditions where efficient pan-retinal delivery is needed, offering therapeutic effect while preventing further deterioration of the compromised retina.

Beyond providing new therapeutic options, AAV2.PN168 enhances the efficacy of established procedures using AAV via intravitreal injection. Recent studies with AAV2.7m8 have demonstrated the practicability of intravitreally injected AAV in treating wAMD. However,

clinical trials have shown that some patients with wAMD who received AAV2.7m8-mediated anti-VEGFA treatment required dose-related supplemental injections of VEGFA-targeting protein, such as aflibercept, from post-injection week 4 onwards,[43] potentially due to insufficient transduction efficacy of AAV2.7m8.

Compared to AAV2.7m8, our results indicate that AAV2.PN168 exhibits a higher transduction rate in retinal ONL, which consists primarily of photoreceptors, and shows improved therapeutic effects after intravitreal injection. This suggests its substantial clinical relevance. Intriguingly, relative to other AAV2 derivatives, an elevated proportion of ONL cells in the critical area of visual function, the macula, were transduced in subjects injected with AAV2.PN168, further supporting its therapeutic efficacy.

In addition to the advances in therapeutic application, in contrast to AAV2.7m8, which was developed through manual directed evolution, AAV2.PN168 was engineered utilizing AI models. While AI has gained importance in AAV engineering in recent years,[23–30] most studies in this field have focused on fundamental aspects such as AAV viability,[29,30] with only a few addressing organ-/tissue targeting.[26–28] Our findings provide substantial evidence supporting the use of AI in developing more effective retina-targeting AAV capsids. Furthermore, our research includes not only mouse models but also monkeys, yielding results with significant translational relevance. Additionally, the relatively short development period (approximately one year) of AAV2.PN168 showcases the acceleration facilitated by AI in creating novel treatments for ocular diseases. Overall, our study emphasizes the profound impact of AI on medical science as a whole.

Despite these advances, several limitations remain. As previously mentioned, AAV2.PN168 was engineered using an AI-guided approach through mutagenesis within the AAV2 VP3 heparin-binding domain, specifically at Cap positions $R_{587/588}$. In a heparin competition assay, AAV2.PN168 exhibits decreased resistance to free heparin compared to AAV2.WT (Figure S10), suggesting that altered capsid-receptor interactions may contribute to its enhanced transduction profile. Whether this phenotype reflects direct modulation of primary receptor engagement[33,44] or is mediated by conformational changes in corresponding capsid regions[32,45] remains to be further investigated. These conformational changes have been demonstrated in previous studies to facilitate the co-receptor-mediated endocytosis of AAV capsids following their initial cell attachment.[32,44] The investigation into the mechanisms underlying the enhanced transduction profile of AAV2.PN168 not only expands our understanding of AAV physiology but also facilitates further AI-guided AAV engineering. With generally limited computing power, more efficient generation of novel AAV capsids with high performance can only be attained by more precisely directed mutagenesis. Another practical limitation is that, in this study, we mainly utilized CAG promoter, a ubiquitous promoter, to characterize the transduction properties of AAV2.PN168 in the retina. Though previous studies have demonstrated stable expression of packaged genes mediated by the CAG (or short CAG) promoter in retinal tissue,[38,46,47] it is important to note that promoters such as CAG and CMV, which benefit from their CpG-rich nature for driving robust expression, are also vulnerable to epigenetic silencing due to DNA methylation.[48–50] Therefore, future studies should evaluate more promoters, especially endogenous ones, which are more adaptive and resilient to DNA methylation and subsequent silencing.[51,52] Finally, we acknowledge that the NHP sample size in this study is limited. The AAV2.PN168 NHP data presented in Figures 3A–3C, 3E, and S5G were each obtained from a single eye of a distinct monkey (three different monkeys in total), with AAV2.7m8 administered in the contralateral eye of the same animal as an external benchmark for the 5E10 vg/eye dose comparison (Figure 3E). While this precludes analysis across biological replicates, it is noteworthy that AAV2.PN168 consistently demonstrated robust pan-retinal transduction across all three independent NHP subjects: it emerged as the top-performing variant in the small-library validation (Figures 3A–3C), outperformed AAV2.7m8 at an equivalent dose (Figure 3E), and achieved extensive retinal coverage at a 10-fold higher dose (Figure S5G). This cross-individual consistency, together with the concordant findings in mouse models, supports the robustness of the AAV2.PN168 phenotype. Nevertheless, given the inherent variability of NHP models and the regulatory requirements for ocular gene therapy, expanded replication across additional animals and extended longitudinal follow-up will be essential prior to translating AAV2.PN168 to human applications.

In addition to the limitations noted previously, further studies are required to enhance specific therapeutic effects before clinical translation. For instance, in this study, we used WT *hGUCY2D* sequence to treat LCA1 pathology. However, earlier work has shown that codon optimization can increase protein production from the packaged sequence,[53] enabling lower AAV doses[54] and improving the safety profile. Although AAV2.PN168-hGRK1-hGUCY2D treatment resulted in evident retinal function recovery in LCA1 mice, their vision remained largely below that of WT controls. Our results align with the current preclinical and clinical trials on treating LCA1 using AAV (no ONL thickness recovery, data not shown),[16,36] demonstrating AAV2.PN168's modified safety profile while also highlighting opportunities for enhanced protein expression to achieve better functional restoration—codon optimization being one promising approach.

For wAMD, while VEGFA antibodies and anti-VEGFA fusion proteins remain the standard treatments, several new strategies have emerged in recent years. For instance, the bispecific antibody faricimab,[55] which targets angiopoietin-2 in addition to VEGFA, has been approved in both the USA and the EU. Additionally, endogenous VEGFA ligands like soluble VEGFA receptor 1 (sFLT1) have been demonstrated to be well tolerated and effective in wAMD patients.[56,57] The outcomes from these new strategies suggest that the next step in bringing AAV2.PN168 to clinical application involves further validation with more therapeutically effective sequences, leading to improved treatments for wAMD.

Collectively, in this study, we present AAV2.PN168, a novel AAV2 derivative engineered using AI-guided approach. We demonstrated that AAV2.PN168 mediates high transduction efficacy of retinal tissue and shows evident therapeutic effects while been utilized for treating various retinal degeneration. With highlighting the transformative potential of AI in medical science, our results support that this AAV2.PN168 carries significant clinical relevance, and may offer benefits for treating an even broader range of retinal pathologies, such as X-linked retinoschisis[21] and other types of Leber's congenital amaurosis,[58] which are treatable using AAV-mediated gene therapy.

MATERIALS AND METHODS

AI models for predicting AAV viability and targeting

As illustrated in Figure S9, both the NGS-based scores (viability and targeting scores) and NGS-detected sequences were used to train the models. In the acquired DNA sequences, each nucleotide was encoded via one-hot encoding to generate a sequence-specific matrix (see Equation 1 in the following text):

$$M_{nt}^{n\times 4}. \quad \text{(Equation 1)}$$

Where "n" denotes the length of the DNA sequence. In parallel, the DNA sequences were translated into sequences of corresponding amino acids. Subsequently, amino acid features, namely extended-connectivity fingerprints (ECFPs)[59] were extracted using RDKit software (public domain) to construct matrix (see Equation 2 in the following text):

$$M_{aa}^{m\times l}. \quad \text{(Equation 2)}$$

Where "m" denotes the length of the amino acid sequence, and "l" denotes the bit length of the ECFPs features.

To establish our AI models, Convolutional Neural Networks (CNNs) and Long Short-Term Memory networks (LSTMs) were integrated. The CNNs were first applied to extract codon-level features from the DNA sequences. These features were then merged with ECFPs through tensor concatenation. The merged features were subsequently processed by the LSTMs to capture sequence-wide characteristics, which were then used to predict viability and targeting scores of AAVs (cap insertions) via a multilayer perceptron (MLP).

Sequence generator

To generate new sequences suitable for insertion between the viral protein 3 (VP3) residues $R_{587/588}$ of AAV2.WT, we developed a sequence generator that operates as follows: (1) select the top N mutants from the NGS library to form the initial parental population. (2) Generate an offspring population through base mutation and fragment recombination. (3) Use AI models to predict the viability and targeting scores of the offspring. (4) Merge the parental population with the offspring population and apply Non-Dominated Sorting Genetic Algorithms II (NSGA-II)[60] using Distributed Evolutionary Algorithms in Python (DEAP)[61] to sort and select the top N sequences for a new parental population. (5) If the maximum generation round is not reached, repeat steps 2 through 4; otherwise, proceed to step 6. (6) Using all historical mutants, apply greedy filtering to exclude individuals with excessively small edit distances, ensuring both diversity in the selected mutant population and the retention of high-scored mutants.

Plasmid construction

To construct the plasmids for the initial AAV2-derived capsid library, an insertion cassette containing 7 to 12 random amino acids, based on the NNK principle, was flanked by NheI-HF (New England Biolabs, USA) and NotI-HF (New England Biolabs, USA) restriction sites in a 5′-3′ orientation. This cassette was assembled with the pAAV2/2 backbone (Addgene, USA) through commercial synthesis provided by Twist Bioscience (USA) and subcloning using the Gibson Assembly Master Mix (New England Biolabs, USA). The resulting constructs were validated by amplicon sequencing. To construct pAAV2-CAG-barcoded eGFP, the plasmid pAAV CAGG eGFP (Addgene, USA) was digested using BsrGI-HF (New England Biolabs, USA) and XhoI (New England Biolabs, USA) to linearize it. Designed barcodes, consisting of 15 bp sequences with flanking homology arms, were synthesized as double-stranded DNA fragments (Integrated DNA Technologies, USA). These fragments were inserted into the linearized plasmid using the Gibson Assembly Master Mix (New England Biolabs, USA). Each pAAV2-CAG-barcoded eGFP plasmid underwent complete plasmid sequencing via Sanger sequencing to ensure successful assembly. To construct the pAAV2-CAG-LUC plasmid, the pAAV CAGG eGFP plasmid (Addgene, USA) was digested using EcoRI-HF (New England Biolabs, USA) and BglII (New England Biolabs, USA) enzymes to remove the eGFP fragment. Double-stranded DNA fragments containing luciferase cDNA with flanking homology arms (Integrated DNA Technologies, USA) were then integrated into the linearized plasmids using the Gibson Assembly Master Mix (New England Biolabs, USA). For the construction of the pAAV2-CAG-anti-VEGFA plasmid, the pAAV CAGG eGFP plasmid (Addgene, USA) was digested using EcoRI-HF (New England Biolabs, USA) and BglII (New England Biolabs, USA) enzymes to remove the eGFP fragment. Subsequently, double-stranded DNA fragments containing anti-VEGFA fragment cDNA with flanking homology arms (Integrated DNA Technologies, USA) were integrated into the linearized plasmids using the Gibson Assembly Master Mix (New England Biolabs, USA).

AAV pproduction and titration

To package the AAVs, HEK293T cells (ATCC, USA) served as the host system. For the AAV libraries, cells were seeded in 100 mm culture dishes at a density of 6E6 cells per dish for 24 h, followed by transfection using PEI MAX (Polysciences, USA). For individual AAV production, cells were transfected with 4.86 μg of pAdDeltaF6 (Addgene, USA) and 3.65 μg of pAAV2.variant per dish, calculated at a molar ratio of 1:1. For the production of AAV2.WT and AAV2-derived variants containing barcoded eGFP, luciferase or anti-VEGFA fragment, cells were transfected with 4.86 μg of pAdDeltaF6 (Addgene, USA), 3.65 μg of pAAV2/2 or pAAV2.variant, and 3.65 μg of the ITR-containing transgene plasmid per dish, calculated at a

molar ratio of 1:1:1. Following transfection, the cells were incubated for 72 h before harvest. At the time of harvest, the supernatant of the culture medium was carefully collected, while the cells were resuspended in a lysis buffer composed of 50 mM Tris and 150 mM NaCl at pH 8.5. These resuspended cells underwent three cycles of freeze-thawing using a dry ice/ethanol bath to ensure complete lysis. The lysate was incubated at 37°C for 30 min and treated with 30 U/mL Benzonase nuclease (Merck Millipore, Germany). Subsequently, the lysate was centrifuged twice—first at 2,000 rpm for 2 min to remove cellular debris, and then at 10,000 rpm for 10 min to obtain clarified supernatant containing the AAV particles. The collected supernatant was subjected to iodixanol density gradient centrifugation for precise purification of the AAVs. Finally, buffer exchange into phosphate-buffered saline (PBS) was performed using 100 kDa MWCO Amicon Ultra Centrifugal Filters (Merck Millipore, Germany).

To titrate the produced AAVs, quantitative PCR (qPCR) was utilized. Specifically, the assay employed TaqMan Fast Advanced Master Mix (Applied Biosystems, USA), alongside forward and reverse primers designed to amplify the AAV2 ITR sequence (Table S1). The reactions were conducted using a Bio-Rad T100 Thermal Cycler (Bio-Rad, USA) under the following conditions: an initial denaturation step at 94°C for 5 min, followed by 45 cycles of denaturation at 94°C for 15 s and extension at 60°C for 30 s. The AAV titters were calculated based on standard curves generated using ITR DNA as a reference.

Electron microscopy

To evaluate the empty capsid ratio, electron microscopy was utilized. Specifically, 20 μL of the resuspended AAV sample (5E12 vg/mL) was applied dropwise onto 200-mesh grids and incubated at room temperature for 10 min. The grids were then negatively stained using 2% phosphotungstic acid for 3 min, after which excess liquid was carefully removed using filter paper. Subsequently, the prepared samples were scanned using a JEM1400 transmission electron microscope (JEOL, Japan), and the resulting images were analyzed semi-automatically using the point tool in ImageJ software.

Animals

For experiments involving WT mice, naive *C57BL/6J* and *BALB/cJ* mice were purchased from Charles River Laboratories (Charles River Laboratories, USA). The *GucyDKO* strain was established in-house and is currently commercially available at Cyagen under the strain name: *C57BL/6JCya-Gucy2e^{em1}Gucy2f^{em1}/Cya*. To create this strain, mouse Gucy2e gene (exons 4–11) and Gucy2f gene (exon 3) were knocked out. The *hVEGFA-TG* strain was established in-house and is currently commercially available at Cyagen under the strain name: *C57BL/6JCya-Tg(bRho-VEGFA)/Cya*. To create this strain, the bovine rhodopsin-hVEGF (GenBank: NM_001171626) construct was injected into fertilized eggs of *C57BL/6J* mice. Transgenesis was validated using qPCR to detect the mRNA of *hVEGFA* in the retinal tissue of *hVEGFA-TG* mice (Table S1). In this study, mice of mixed sexes aged 6–8 weeks were included. They were housed in the animal facility at Cyagen Biomodels under specific pathogen-free conditions (21 ± 1°C, maintained on a 12 h light/12 h dark cycle) with *ad libitum* access to food and water. All protocols and procedures involving mice complied with the regulations of the facility and were approved by the Institutional Animal Care and Use Committee (IACUC) of Cyagen Biomodels (Guangzhou) Co., Ltd. (permit number: GACU25-SY074).

Four cynomolgus monkeys (*Macaca fascicularis*) purchased from Landao-Bio (Landao-Bio, China) were used in this study and were tested for the AAV2 inhibition rate of their serum. The monkeys, aged 5–10 years and weighing between 4.75 and 8.3 kg, were housed in rooms under a 12 h light/12 h dark cycle at temperatures ranging from 22°C to 26°C. The monkeys were fed twice daily with monkey maintains feed (Keaoxieli, China). Monkeys were provided with standard care, and their health status was monitored twice daily by a certified veterinarian. The monkeys were maintained at the Guangdong Laboratory Animals Monitoring Institute, an Association for Assessment and Accreditation of Laboratory Animal Care (AAALAC)-accredited facility. All protocols and procedures involving monkeys followed regulations of Guangdong Laboratory Animals Monitoring center and approved by its IACUC (permit number: IACUC2025112).

Transduction inhibition rate assay

To evaluate the transduction inhibition rate of monkey serum on AAV2, HEK293T cells (ATCC, USA) were utilized as the host system. The cells were seeded at a density of 2E5 per well in a 24-well microplate and incubated for 24 h. Monkey serum (or fetal bovine serum [FBS], Gibco, USA) was diluted with Dulbecco's Modified Eagle's Medium (DMEM) (Corning, USA) at a 1:20 ratio and then mixed with AAV2.WT-CMV-eGFP for 24 h at 37°C. This mixture was then applied to the cells at a genomic multiplicity of infection (MOI) of 1,500. After 48 h of incubation, cells were digested with 0.25% trypsin at 37°C, washed with PBS, resuspended in 500 μL PBS and filtered using a 75 μL filter prior to performing FACS analysis. For proper gating, HEK293T cells incubated only with DMEM were utilized (Figure S8). The proportion of GFP$^+$ cells in each sample was assessed using an Agilent NovoCyte Flow Cytometer (Agilent Technologies, USA) with the FITC filter set, following the manufacturer's instructions. For both data collection and analysis, NovoExpress Software 1.5.0 was employed (Agilent Technologies, USA). The transduction inhibition rate was calculated by subtracting the proportion of GFP$^+$ cells in the samples treated with the monkey serum/DMEM mixture from those treated with the FBS/DMEM mixture, and dividing the difference by the proportion in the FBS-treated group.

Intravitreal injection and retinal tissue extraction in mice and monkeys

For intravitreal injections in mice, pre-selected individuals were chosen based on the condition of their eyes and overall health status. The mice were anesthetized using chloral hydrate (I.P. injection, 350 mg/kg), along with analgesia (0.4% oxybuprocaine hydrochloride, eye

drops, single dose). Upon loss of pedal reflex, the mice were placed on a heating pad to maintain their body temperature and positioned under a surgical stereoscope. To expose the eyeball, the eyelids were carefully pulled back. Subsequently, the 33-gauge needle (Hamilton Company, USA) of a 2.5 μL microliter syringe (Hamilton Company, USA) was inserted into the vitreous chamber through the lateral side of the corneal edge, delivering approximately 1.5 μL of AAV-containing solution (3E9-1E11 vg/eye), PBS or ranibizumab (Meibio, China) at a controlled pace. After the infusion was completed, the needle was gently withdrawn, and 0.25% erythromycin ointment was applied to the eye. Once the anesthesia had worn off, the mice were returned to clean cages and housed individually for a week to ensure full recovery. Retinal tissue extraction was performed 21 days post-AAV injection. During this process, the mice were anesthetized, and the eyes were extracted for subsequent experiments.

For intravitreal injections in monkeys, eligible monkeys, with healthy eye status and insignificant levels (<5% transduction inhibition rate) of serum AAV2 neutralizing antibodies, were anesthetized using ketamine (15 mg/kg, intramuscular [I.M.] injection), coupled with analgesia (0.5% proparacaine, eye drops, single dose). To conduct the intravitreal injection, a BD Ultra-Fine U-100 syringe with a 31-gauge needle was employed (Becton Dickinson, USA). The needle was inserted through the sclera approximately 3 mm lateral to the corneal edge of the eye, followed by the infusion of 80–100 μL of AAV-containing solution at a controlled pace. Following the infusion, the monkey received a single dose of anti-inflammatory drug (5 mg/kg, ketoprofen, I.M. injection) and daily antibiotics (3 mg/mL tobramycin combined with 1 mg/mL dexamethasone, eye drops, once per day) for 3 consecutive days post-AAV injection. As a long-term immunosuppression strategy, from 3 days before the intravitreal injection of AAV to the endpoint (21 days post-AAV injection), the monkeys received 1 mg/mL tacrolimus (eye drops, twice per day). After 21 days, the monkeys were anesthetized and euthanized by a certified veterinarian using an intravenous injection of 100 mg/kg pentobarbital sodium. The eyes were then extracted for subsequent experiments.

RNA isolation and cDNA synthesis

Tissue samples were extracted and placed into Trizol Reagent (Invitrogen, USA) before homogenization with 1.4 mm ceramic beads in a BeadBug Tissue Homogenizer (Benchmark Scientific, USA). The homogenization was performed at 4°C in 4 cycles, each lasting 30 s, with 30 s intervals. The homogenized tissue was then subjected to RNA extraction using phenol/chloroform, followed by RNA precipitation with isopropanol and centrifugation at 12,000 *g* at 4°C, and a subsequent wash with 75% ethanol. And the RNA pellet was resuspended in RNase-free water and stored at −80°C until further use.

To synthesize cDNA from RNA samples, residual DNA contamination was removed using a DNA-free DNA Removal Kit (Invitrogen, USA). Following decontamination, cDNA synthesis was performed using approximately 1 μg of RNA as input with the RevertAid First Strand cDNA Synthesis Kit (Thermo Scientific, USA). For each sample, a negative control lacking the reverse transcriptase enzyme was included to confirm that PCR amplicons originated from RNA and not from DNA contamination.

Amplicon sequencing using DNA and RNA

For the plasmid and AAV library, DNA amplicon sequencing was conducted. For mRNA extracted from tissue samples, the RNA was reverse transcribed into cDNA. Using the obtained DNA samples, target genes were amplified through PCR with Q5 High-Fidelity DNA Polymerase (New England Biolabs, USA) and primers (Table S1). The PCR products were then purified using the QIAquick PCR Purification Kit (QIAGEN, USA) to eliminate contaminants and ensure high-quality outputs.

Sequencing was performed on the Illumina NovaSeq 6000 System (Illumina, USA) at a depth of approximately 50×, minimizing potential bias. Final readouts ensured a minimum coverage of over 95% unique variants, with each variant detected in more than 10 reads.

scRNA-seq

For both mouse and monkey retinas, scRNA-seq samples were prepared using 10× Chromium Single Cell 3′ kits v.3 (10× Genomics, USA). Retinal tissues were homogenized with 1.4 mm ceramic beads using a BeadBug Tissue Homogenizer (Benchmark Scientific, USA). The resulting single-cell suspension was captured using the 10× Chromium system, which partitions cells into gel beads-in-emulsion (GEMS). Within each GEM, mRNAs from individual cells were reverse transcribed and tagged with unique molecular identifiers (UMIs) and 10× Genomics Barcodes. Purified cDNA was subsequently amplified via PCR and further purified using SPRIselect bead-based reagent (Beckman Coulter, USA).

The amplified cDNA underwent fragmentation with the Bioruptor Plus sonication device (Diagenode, USA) to achieve a target length of ∼300 bp. The final library construction involved steps such as end repair, A-tailing, adapter ligation, and sample index PCR, following the 10× Single Cell 3′ workflow guidelines. Additionally, targeted sequencing analysis was performed on the 10×-prepped cDNA using PCR amplification with Q5 High-Fidelity DNA Polymerase (New England Biolabs, USA) to isolate sequences of barcoded GFP. The pooled 10× library underwent deep sequencing using the Illumina NovaSeq 6000 System (Illumina, USA). Sequencing depth was targeted at 300 million reads per sample for standard scRNA-seq analysis.

Analysis of sequencing data

To analyze the data from amplicon sequencing, the raw data were first subjected to an initial quality check using FastQC (http://www.bioinformatics.babraham.ac.uk/projects/fastqc/) and further analyzed with customized Python (public domain) scripts. Sequences were binned based on the presence or absence of inserts; insert-containing sequences were then compared to a baseline reference sequence, and error-free reads were tabulated based on the

frequency of each unique insert detected. Inserts were translated and normalized to compute scores.

To analyze the data obtained from scRNA-seq, the Python toolkit Scanpy (public domain)[62] was utilized following established methods. First, the top 50 principal components of the gene expression matrix were calculated. Using this reduced dimensionality, Euclidean distances between cells were determined. The closest 0.5% neighbors for each cell were kept and embedded into a neighborhood graph using the uniform manifold approximation and projection (UMAP) algorithm. Leiden clustering was then applied to the single-cell neighborhood graph.

To identify cell types in retinal tissues from mice and monkeys, a differential gene expression analysis was performed using Scanpy's "rank_gene_group" feature. A hypergeometric test was used to evaluate the significance of the intersection between marker genes for each cluster and marker genes that are previously published.[63–65] Bonferroni p value correction was used to control the probability of type I errors for multiple hypothesis tests. Finally, the positivity rate of AAV sequences within each cell type was calculated.

ERG

Mouse retinal function was assessed using the Celeris small animal visual electrophysiology system (Model D430-P-10, Diagnosys). Before conducting the tests, animals were dark-adapted overnight, and all subsequent procedures were performed under red light. Mice were anesthetized via intraperitoneal injection of 1.25% (w/v) tribromoethanol (0.2 mL/10 g body weight), followed by pupil dilation with 0.5% tropicamide eye drops. The corneal surface was then topically anesthetized with 0.4% oxybuprocaine hydrochloride. For signal recording, a contact lens electrode was placed on the cornea. Scotopic ERG recordings were obtained at flash intensities of 2.5 $cd \cdot s/m^2$ and 10 $cd \cdot s/m^2$, with five consecutive responses averaged at each intensity. Following the scotopic measurements, mice were light-adapted for 5 min under a background illumination of 30 $cd \cdot s/m^2$. Subsequently, photopic responses were recorded at two increasing stimulus intensities (25 $cd \cdot s/m^2$ and 100 $cd \cdot s/m^2$), averaging ten responses per intensity.

OMR

To assess OMR time, mice were placed on an elevated central platform and allowed to move freely. Surrounding the platform, 4 liquid crystal display (LCD) screens projected a virtual cylinder displaying a vertical sinusoidal grating. The grating rotated either clockwise or counterclockwise at a randomly assigned direction, with an angular velocity of 12°/s. The visual stimulus was presented at a spatial frequency of 0.2 cycles per degree and 100% contrast. Each stimulation trial lasted 60 s, followed by a 20-s inter-trial interval. The direction of rotation was reversed every 6 s to ensure balanced exposure. Each mouse completed at least three test sessions, with each session including five clockwise and five counterclockwise stimuli. For statistical analysis, results from all trials were averaged.

OCT

Mice were anesthetized via intraperitoneal injection of 1.25% (w/v) tribromoethanol (0.2 mL/10 g body weight), followed by pupil dilation with 0.5% tropicamide eye drops. The corneal surface was then anesthetized topically with 0.4% oxybuprocaine hydrochloride. Fundus and OCT examinations were conducted using the Micron IV retinal imaging microscope (Phoenix Research Laboratories). Once anesthetized, each mouse was placed on the imaging platform. To begin, the fundus was brought into clear view with the optic disc centered, and a color fundus photograph was captured. Subsequently, OCT scans were performed along both horizontal and vertical planes passing through the optic disc. Signal optimization was carried out, and cross-sectional images were recorded once the retinal layers were distinctly visualized.

Luciferase-based *in vivo* imaging

AAV-mediated luciferase expression was evaluated using the luciferase-luciferin reaction combined with *in vivo* imaging. Mice were anesthetized with isoflurane and given an I.P. injection of D-luciferin at a dose of 150 mg/kg (Yeasen, China). Luminescence from the eyes of the mice was captured and analyzed utilizing the AniView100 Multi-mode *in vivo* Animal Imaging System (BLT-Imaging, China), coupled with AniView software (BLT-Imaging, China) for image acquisition and analysis.

In vivo fundoscopy

To evaluate AAV-mediated GFP expression in mouse retinas, *in vivo* fundoscopy was performed using the MICRON IV Retinal Imaging Microscope (Phoenix MICRON, USA). Briefly, mice were anesthetized with 1.25% (w/v) tribromoethanol and securely positioned on the imaging stage. Bright-field imaging was initially used to locate the appropriate fundus area, followed by fluorescence scanning to detect the GFP signal. After capturing the images, the mice were returned to a clean cage for recovery.

To evaluate the condition of the monkeys' eyes before and after the AAV injection via *in vivo* fundoscopy, the monkeys were anesthetized with 15 mg/kg ketamine administered (I.M. injection). Subsequently, 5 mg/mL tropicamide combined with 5 mg/mL phenylephrine hydrochloride (eye drops, single dose) was applied to induce mydriasis, followed by a fundoscopic examination using the TRC-NW7SF Mark II digital fundus imaging system (TOPCON, Japan).

Fluorescein angiography

To evaluate subretinal exudation, fluorescein angiography was used in combination with fundoscopy, utilizing the MICRON IV Retinal Imaging Microscope (Phoenix MICRON, USA). Prior to imaging, 1% AK-FLUOR solution (fluorescein injection) (Akorn, USA) was I.P. administered at a dose of 60 mg/kg. Fundoscopy of the mouse was conducted within 1 min following the standard fundoscopy procedures detailed in the methods section.

Sample preparation, immunostaining, and image acquisition

To assess AAV-mediated GFP expression in mouse retinal tissue, the mice were euthanized via cervical dislocation. The eyes were removed and post-fixed in 4% paraformaldehyde (PFA)/PBS for 1 h at room temperature. The fixed eyes were subsequently incubated with 10%, 20%, and 30% sucrose at 4°C for gradient dehydration. After successful dehydration, the eyes were embedded in Tissue-Tek optimal cutting temperature compound (Sakura Finetek, Japan). The embedded eyes were then processed using a CM1860 Cryostat (Leica, Germany) and sectioned into 10 μm-thick sections. For the monkey retinal tissue, euthanasia was conducted using pentobarbital sodium, and the eyes were extracted. To ensure thorough fixation of the retinal tissue, the eyes were processed as previously described with slight modifications.[66] Specifically, an incision was made into the vitreous chamber at the pars plana, followed by immersion in 4% PFA/PBS at 4°C for 3.5 h. The fixed eyes were subsequently incubated with 10%, 20%, and 30% sucrose at 4°C for gradient dehydration before being embedded in Tissue-Tek optimal cutting temperature compound. These samples were then sectioned in a CM1860 Cryostat (Leica, Germany) into 10 μm-thick sections.

Sections from both mice and monkeys were subjected to identical immunostaining processes for GFP. Briefly, the sections were carefully mounted on SuperFrost Ultra Plus GOLD adhesion slides (Epredia, UK). Residual optimal cutting temperature compound was washed away, and the sections were blocked with 5% bovine serum albumin (BSA) (Sigma-Aldrich, USA)/PBS for 1 h at room temperature. They were then incubated with an anti-GFP antibody (Invitrogen, USA) at 4°C overnight. After incubation, the sections were thoroughly washed with PBS to remove excess antibody solution and counterstained with DAPI (Thermo Scientific, USA). Finally, the stained sections were mounted using ProLong Gold antifade mountant (Invitrogen, USA) and covered with coverslips. The stained sections were then subjected to microscopic scanning. All materials are listed in Table S4.

For image acquisition, mouse eye sections were imaged using an Olympus BX52 upright microscope (Olympus, Japan) with cellSens 3.2 (Olympus, Japan). Monkey eye sections were initially scanned using the THUNDER imaging system (Leica, Germany) to acquire overview images using LAS X 3.10.0 (Leica, Germany). For detailed imaging of monkey retinal regions, z stack images were acquired using a Stellaris 5 confocal microscope (Leica, Germany) coupled with LAS X 4.7.0 (Leica, Germany).

Image analysis

To quantify the range of the GFP$^+$ area in the monkey retina, a geometry-based quantitative approach was utilized. In this method, the retina was fan-shaped based on the overview section image (A°), and the total angle (B°) of the GFP$^+$ range in the retina was measured on 3 non-consecutive sections (interval >10 μm) within macula-containing sectional planes. The proportion of the GFP$^+$ range was calculated as the ratio of B° relative to A°.

To quantify GFP$^+$ ONL cells in various regions of the monkey retina, raw images were converted into.ims files and analyzed using Imaris software (Bitplane, UK) through 3D reconstruction. Specifically, cells across all retinal layers were reconstructed in 3D based on their nuclei (DAPI) using the spot module. Subsequently, GFP signals within the regions of interest were reconstructed in 3D utilizing the surface module. Following this process, only the GFP signals from the ONL were selected, and the DAPI spots located within the GFP surface were quantified. The proportion of GFP$^+$ ONL cells was calculated as the ratio of GFP$^+$ ONL cells to all ONL cells, based on the DAPI signal.

To measure the GFP mean fluorescence intensity in mouse fundus images and retinal sections, gray value analysis was performed using ImageJ software. The fluorescence intensity (gray value) of each pixel within a single fluorescence channel (i.e., GFP) was analyzed and quantified. The mean fluorescence intensity for the region of interest was calculated by dividing the total fluorescence intensity of the region by the area of the region of interest.

To evaluate the recovery ratio of subretinal exudation in *hVEGFA-TG* mice following anti-VEGFA treatment, the exudation area—defined by the fluorescein sodium signal coverage—was analyzed using Image-Pro Plus 6.0 software (Media Cybernetics, USA). The recovery ratio was determined by comparing the exudation area measured before treatment to that at the endpoint, expressed as a percentage of the initial measurement.

ddPCR

To quantify the AAV2-mediated expression of mRNA for the anti-VEGFA antibody in *hVEGFA-TG* mice, ddPCR was employed. Retinal tissue was harvested from mice, and mRNA was subsequently extracted. cDNA synthesis was carried out using the extracted mRNA with the RevertAid First Strand cDNA Synthesis Kit (Thermo Scientific, USA). PCR amplification was performed using the SG-2000 PCR Amplifying Apparatus (RainSure Scientific, China). Specifically, 8 μL of obtained cDNA was mixed with 10 μL of digital PCR buffer (RainSure Scientific, China) and 2 μL of primer mix (Table S1). The thermal cycling process followed these steps: preheating at 95°C for 10 min, 40 cycles of denaturation at 94°C for 30s, annealing at 56°C for 60s, and a final extension at 98°C for 10 min, followed by cooling at 20°C for 2 min. After amplification, the PCR droplets were transferred to a DScanner4-1000 Biochip Scanner (RainSure Scientific, China) for scanning. Results were analyzed using GeneCount Analysis System software (RainSure Scientific, China).

Heparin competition assay

For the heparin competition assay, cells were seeded at 1E5 cells per well in 24-well plates on day 0 and transduced at various viral genome copy numbers per cell. Where indicated, heparin sodium salt (Sigma-Aldrich, USA) was added from a 100× stock solution to final concentrations of 100 or 400 μg/mL. At 72 h post-transduction, cells were harvested using TrypLE Express (Thermo Fisher

Scientific, USA), and GFP expression was analyzed by flow cytometry on a BD LSRFortessa (BD Biosciences, USA). Data were analyzed using FlowJo (FlowJo LLC, USA).

Statistics

GraphPad Prism 10 (GraphPad Software, USA) was used to perform all statistical analyses. Two-tailed Student's *t* tests or Mann-Whitney tests were applied for comparisons between two different groups. One-way ANOVA followed by Dunnett's post-hoc tests or Kruskal-Wallis tests were employed for multiple group comparisons. For longitudinal treatment assessment in *hVEGFA-TG* mice, repeated measures ANOVA with Geisser-Greenhouse correction, followed by Dunnett's post-hoc tests, was utilized. All data are presented as mean ± SD unless specified otherwise. A p value of less than 0.05 was considered statistically significant.

DATA AND CODE AVAILABILITY

Raw data from this study are available from the corresponding author upon reasonable request. Additional data are accessible in the Sequence Read Archive under the BioProject number: PRJNA1269792. The viability model is now accessible online via the link: https://rddc.tsinghua-gd.org/tool/aav_viability, allowing for the prediction of the viability of AAV2-derived variants based on their sequences. The AI model code can be called using an Application Programming Interface (API). A case demonstration and the API are accessible via the link: https://github.com/Huatsing-Lau/YG-AAV-Predictor. Additionally, the code and data for the AI-related viability and targeting analyses (Figures 2B–2G and S9) and for the iterative process (Figure S4), can be found at the same GitHub link.

ACKNOWLEDGMENTS

This work has been sponsored by YIMA Gene and Cyagen Biosciences. M.C., Q. Zhang, L.Y., C.G., J.X., C.W., Q. Zheng, L.L., and S.R. performed experiments while working at YIMA Gene. H.L., C.L., P.C., S.Z., X.M., J.W., and L.H. performed experiments while working at Cyagen Biosciences.

AUTHOR CONTRIBUTIONS

M.C. and S.R. conceived this study. M.C., H.L., L.C., and S.R. designed the experiments. L.C., Q. Zhang, Q. Zheng, C.L., and X.M. performed animal experiments. H.L., C.G., P.C., and S.Z. performed bioinformatic data analyses and established AI frameworks. L.Y., L.L., and C.W. conducted AAV production. J.X. carried out NGS analyses. M.C., Y.Z., L.H., and S.R. analyzed the results of other experiments and interpreted the readouts of all experiments. M.C. and S.R. wrote the manuscript with critical input from J.W., L.H., Y.L., and L.C. All authors reviewed and commented on the manuscript.

DECLARATION OF INTERESTS

L.H., H.L., and C.G. are listed as inventors on patents pertaining to the AI frameworks introduced in this study. S.R., L.Y., Q. Zhang, L.L., C.W., and Q. Zheng hold patents for AAV2.PN168 and other AI-designed AAV2 derivatives utilized in this research, along with their therapeutic applications. S.R. and L.H. are founders of YIMA Gene, a company specializing in the development of AI-engineered AAVs and their applications. YIMA Gene is a subsidiary of Cyagen Biosciences.

SUPPLEMENTAL INFORMATION

Supplemental information can be found online at https://doi.org/10.1016/j.omta.2026.201806.

REFERENCES

1. Panneman, D.M., Hitti-Malin, R.J., McKibbin, M., de Bruijn, S.E., Boonen, E.G.M., Vincent, A.L., Vargas, G., Corominas, J., Astuti, G., Gilissen, C., et al. (2025). Expansion of the ABCA4-Associated Retinopathy Spectrum: Severe Variants Can be Associated With Early-Onset Severe Retinal Dystrophy. Investig. Ophthalmol. Vis. Sci. *66*, 19. https://doi.org/10.1167/iovs.66.6.19.
2. Wong, W.L., Su, X., Li, X., Cheung, C.M.G., Klein, R., Cheng, C.-Y., and Wong, T.Y. (2014). Global prevalence of age-related macular degeneration and disease burden projection for 2020 and 2040: a systematic review and meta-analysis. Lancet Global Health *2*, e106–e116. https://doi.org/10.1016/s2214-109x(13)70145-1.
3. Bennett, J., Wellman, J., Marshall, K.A., McCague, S., Ashtari, M., DiStefano-Pappas, J., Elci, O.U., Chung, D.C., Sun, J., Wright, J.F., et al. (2016). Safety and durability of effect of contralateral-eye administration of AAV2 gene therapy in patients with childhood-onset blindness caused by RPE65 mutations: a follow-on phase 1 trial. Lancet *388*, 661–672. https://doi.org/10.1016/s0140-6736(16)30371-3.
4. Somanathan, S., Breous, E., Bell, P., and Wilson, J.M. (2010). AAV vectors avoid inflammatory signals necessary to render transduced hepatocyte targets for destructive T cells. Mol. Ther. *18*, 977–982. https://doi.org/10.1038/mt.2010.40.
5. Yi, Z., Sun, W., Xiao, X., Li, S., Jia, X., Li, X., Yu, B., Wang, P., and Zhang, Q. (2021). Novel variants in GUCY2D causing retinopathy and the genotype-phenotype correlation. Exp. Eye Res. *208*, 108637. https://doi.org/10.1016/j.exer.2021.108637.
6. Kumaran, N., Moore, A.T., Weleber, R.G., and Michaelides, M. (2017). Leber congenital amaurosis/early-onset severe retinal dystrophy: clinical features, molecular genetics and therapeutic interventions. Br. J. Ophthalmol. *101*, 1147–1154. https://doi.org/10.1136/bjophthalmol-2016-309975.
7. Boye, S.L., O'Riordan, C., Morris, J., Lukason, M., Compton, D., Baek, R., Elmore, D.M., Peterson, J.J., Fajardo, D., McCullough, K.T., et al. (2023). Preclinical studies in support of phase I/II clinical trials to treat GUCY2D-associated Leber congenital amaurosis. Mol. Ther. Methods Clin. Dev. *28*, 129–145. https://doi.org/10.1016/j.omtm.2022.12.007.
8. Blick, S.K.A., Keating, G.M., and Wagstaff, A.J. (2007). Ranibizumab. Drugs *67*, 1199–1206. https://doi.org/10.2165/00003495-200767080-00007.
9. Sophie, R., Akhtar, A., Sepah, Y.J., Ibrahim, M., Bittencourt, M., Do, D.V., and Nguyen, Q.D. (2012). Aflibercept: a Potent Vascular Endothelial Growth Factor Antagonist for Neovascular Age-Related Macular Degeneration and Other Retinal Vascular Diseases. Biol. Ther. *2*, 3. https://doi.org/10.1007/s13554-012-0003-4.
10. Ferrara, N., and Adamis, A.P. (2016). Ten years of anti-vascular endothelial growth factor therapy. Nat. Rev. Drug Discov. *15*, 385–403. https://doi.org/10.1038/nrd.2015.17.
11. Chen, R., Zhu, J., Hu, J., and Li, X. (2023). Antiangiogenic therapy for ocular diseases: Current status and challenges. MedComm–Future Medicine *2*, e33. https://doi.org/10.1002/mef2.33.
12. Constable, I.J., Pierce, C.M., Lai, C.-M., Magno, A.L., Degli-Esposti, M.A., French, M.A., McAllister, I.L., Butler, S., Barone, S.B., Schwartz, S.D., et al. (2016). Phase 2a randomized clinical trial: safety and post hoc analysis of subretinal rAAV. sFLT-1 for wet age-related macular degeneration. EBioMedicine *14*, 168–175. https://doi.org/10.1016/j.ebiom.2016.11.016.
13. Castro, B.F.M., Steel, J.C., and Layton, C.J. (2024). AAV-based strategies for treatment of retinal and choroidal vascular diseases: advances in age-related macular degeneration and diabetic retinopathy therapies. BioDrugs *38*, 73–93. https://doi.org/10.1007/s40259-023-00629-y.
14. Campochiaro, P. (2022). Gene Therapy for Neovascular AMD: Subretinal RGX-314: Phase I/IIa Long-Term Follow-Up Results up to 4 Years. In Oral Presentation at: American Academy of Ophthalmology. October 1.
15. Abbey, A. (2023). Subretinal delivery of investigational ABBV-RGX-314 for neovascular AMD: a phase II pharmacodynamic study. Invest. Ophthalmol. Vis. Sci. *30*, 2023.
16. Yang, P., Pardon, L.P., Ho, A.C., Lauer, A.K., Yoon, D., Boye, S.E., Boye, S.L., Roman, A.J., Wu, V., Garafalo, A.V., et al. (2024). Safety and efficacy of ATSN-101 in patients with Leber congenital amaurosis caused by biallelic mutations in GUCY2D: a phase 1/2, multicentre, open-label, unilateral dose escalation study. Lancet *404*, 962–970. https://doi.org/10.1016/S0140-6736(24)01447-8.
17. Dhoot, D.S. (2023). Suprachoroidal Delivery of Investigational ABBV-RGX-314 for Diabetic Retinopathy: The Phase II ALTITUDE® Study. Invest. Ophthalmol. Vis. Sci. *63*, 1152.
18. Irigoyen, C., Amenabar Alonso, A., Sanchez-Molina, J., Rodríguez-Hidalgo, M., Lara-López, A., and Ruiz-Ederra, J. (2022). Subretinal injection techniques for retinal disease: a review. J. Clin. Med. *11*, 4717. https://doi.org/10.3390/jcm11164717.

19. Wang, J.-H., Gessler, D.J., Zhan, W., Gallagher, T.L., and Gao, G. (2024). Adeno-associated virus as a delivery vector for gene therapy of human diseases. Signal Transduct. Targeted Ther. *9*, 78. https://doi.org/10.1038/s41392-024-01780-w.

20. Lin, R., Zhou, Y., Yan, T., Wang, R., Li, H., Wu, Z., Zhang, X., Zhou, X., Zhao, F., Zhang, L., et al. (2022). Directed evolution of adeno-associated virus for efficient gene delivery to microglia. Nat. Methods *19*, 976–985. https://doi.org/10.1038/s41592-022-01547-7.

21. Dalkara, D., Byrne, L.C., Klimczak, R.R., Visel, M., Yin, L., Merigan, W.H., Flannery, J.G., and Schaffer, D.V. (2013). In vivo–directed evolution of a new adeno-associated virus for therapeutic outer retinal gene delivery from the vitreous. Sci. Transl. Med. *5*, 189ra176. https://doi.org/10.1126/scitranslmed.3005708.

22. Luo, S., Jiang, H., Li, Q., Qin, Y., Yang, S., Li, J., Xu, L., Gou, Y., Zhang, Y., Liu, F., et al. (2024). An adeno-associated virus variant enabling efficient ocular-directed gene delivery across species. Nat. Commun. *15*, 3780. https://doi.org/10.1038/s41467-024-48221-4.

23. Bryant, D.H., Bashir, A., Sinai, S., Jain, N.K., Ogden, P.J., Riley, P.F., Church, G.M., Colwell, L.J., and Kelsic, E.D. (2021). Deep diversification of an AAV capsid protein by machine learning. Nat. Biotechnol. *39*, 691–696. https://doi.org/10.1038/s41587-020-00793-4.

24. Ogden, P.J., Kelsic, E.D., Sinai, S., and Church, G.M. (2019). Comprehensive AAV capsid fitness landscape reveals a viral gene and enables machine-guided design. Science *366*, 1139–1143. https://doi.org/10.1126/science.aaw2900.

25. Guo, J., Lin, L.F., Oraskovich, S.V., de Jesús, J.A.R., Listgarten, J., and Schaffer, D.V. (2024). Computationally guided AAV engineering for enhanced gene delivery. Trends Biochem. Sci. *49*, 457–469.

26. Vu Hong, A., Suel, L., Petat, E., Dubois, A., Le Brun, P.-R., Guerchet, N., Veron, P., Poupiot, J., and Richard, I. (2024). An engineered AAV targeting integrin alpha V beta 6 presents improved myotropism across species. Nat. Commun. *15*, 7965. https://doi.org/10.1038/s41467-024-52002-4.

27. Huang, Q., Chen, A.T., Chan, K.Y., Sorensen, H., Barry, A.J., Azari, B., Zheng, Q., Beddow, T., Zhao, B., Tobey, I.G., et al. (2023). Targeting AAV vectors to the central nervous system by engineering capsid-receptor interactions that enable crossing of the blood-brain barrier. PLoS Biol. *21*, e3002112. https://doi.org/10.1371/journal.pbio.3002112.

28. Eid, F.E., Chen, A.T., Chan, K.Y., Huang, Q., Zheng, Q., Tobey, I.G., Pacouret, S., Brauer, P.P., Keyes, C., Powell, M., et al. (2024). Systematic multi-trait AAV capsid engineering for efficient gene delivery. Nat. Commun. *15*, 6602. https://doi.org/10.1038/s41467-024-50555-y.

29. Marques, A.D., Kummer, M., Kondratov, O., Banerjee, A., Moskalenko, O., and Zolotukhin, S. (2021). Applying machine learning to predict viral assembly for adeno-associated virus capsid libraries. Mol. Ther. Methods Clin. Dev. *20*, 276–286. https://doi.org/10.1016/j.omtm.2020.11.017.

30. De Leonardis, M., Fernandez-de-Cossio-Diaz, J., Uguzzoni, G., and Pagnani, A. (2024). Unsupervised modeling of mutational landscapes of adeno-associated viruses viability. BMC Bioinf. *25*, 229. https://doi.org/10.1186/s12859-024-05823-5.

31. Meumann, N., Cabanes-Creus, M., Ertelt, M., Navarro, R.G., Lucifora, J., Yuan, Q., Nien-Huber, K., Abdelrahman, A., Vu, X.K., Zhang, L., et al. (2023). Adeno-associated virus serotype 2 capsid variants for improved liver-directed gene therapy. Hepatology *77*, 802–815. https://doi.org/10.1002/hep.32733.

32. Levy, H.C., Bowman, V.D., Govindasamy, L., McKenna, R., Nash, K., Warrington, K., Chen, W., Muzyczka, N., Yan, X., Baker, T.S., and Agbandje-McKenna, M. (2009). Heparin binding induces conformational changes in Adeno-associated virus serotype 2. J. Struct. Biol. *165*, 146–156. https://doi.org/10.1016/j.jsb.2008.12.002.

33. Kern, A., Schmidt, K., Leder, C., Müller, O.J., Wobus, C.E., Bettinger, K., Von der Lieth, C.W., King, J.A., and Kleinschmidt, J.A. (2003). Identification of a heparin-binding motif on adeno-associated virus type 2 capsids. J. Virol. *77*, 11072–11081. https://doi.org/10.1128/jvi.77.20.11072-11081.2003.

34. Arjomandnejad, M., Dasgupta, I., Flotte, T.R., and Keeler, A.M. (2023). Immunogenicity of Recombinant Adeno-Associated Virus (AAV) Vectors for Gene Transfer. BioDrugs *37*, 311–329. https://doi.org/10.1007/s40259-023-00585-7.

35. Westhaus, A., Eamegdool, S.S., Fernando, M., Fuller-Carter, P., Brunet, A.A., Miller, A.L., Rashwan, R., Knight, M., Daniszewski, M., Lidgerwood, G.E., et al. (2023). AAV capsid bioengineering in primary human retina models. Sci. Rep. *13*, 21946. https://doi.org/10.1038/s41598-023-49112-2.

36. Baehr, W., Karan, S., Maeda, T., Luo, D.-G., Li, S., Bronson, J.D., Watt, C.B., Yau, K.-W., Frederick, J.M., and Palczewski, K. (2007). The Function of Guanylate Cyclase 1 and Guanylate Cyclase 2 in Rod and Cone Photoreceptors. J. Biol. Chem. *282*, 8837–8847. https://doi.org/10.1074/jbc.M610369200.

37. Khani, S.C., Pawlyk, B.S., Bulgakov, O.V., Kasperek, E., Young, J.E., Adamian, M., Sun, X., Smith, A.J., Ali, R.R., and Li, T. (2007). AAV-mediated expression targeting of rod and cone photoreceptors with a human rhodopsin kinase promoter. Investig. Ophthalmol. Vis. Sci. *48*, 3954–3961. https://doi.org/10.1167/iovs.07-0257.

38. McClements, M.E., Steward, H., Atkin, W., Goode, E.A., Gándara, C., Chichagova, V., and MacLaren, R.E. (2022). Tropism of AAV Vectors in Photoreceptor-Like Cells of Human iPSC-Derived Retinal Organoids. Transl. Vis. Sci. Technol. *11*, 3. https://doi.org/10.1167/tvst.11.4.3.

39. Boye, S.E., Alexander, J.J., Boye, S.L., Witherspoon, C.D., Sandefer, K.J., Conlon, T.J., Erger, K., Sun, J., Ryals, R., Chiodo, V.A., et al. (2012). The human rhodopsin kinase promoter in an AAV5 vector confers rod- and cone-specific expression in the primate retina. Hum. Gene Ther. *23*, 1101–1115. https://doi.org/10.1089/hum.2012.125.

40. Yadav, S.P., Hao, H., Yang, H.J., Kautzmann, M.A.I., Brooks, M., Nellissery, J., Klocke, B., Seifert, M., and Swaroop, A. (2014). The transcription-splicing protein NonO/p54nrb and three NonO-interacting proteins bind to distal enhancer region and augment rhodopsin expression. Hum. Mol. Genet. *23*, 2132–2144. https://doi.org/10.1093/hmg/ddt609.

41. Zack, D.J., Bennett, J., Wang, Y., Davenport, C., Klaunberg, B., Gearhart, J., and Nathans, J. (1991). Unusual topography of bovine rhodopsin promoter-IacZ fusion gene expression in transgenic mouse retinas. Neuron *6*, 187–199. https://doi.org/10.1016/0896-6273(91)90355-4.

42. Wimmer, T., Lorenz, B., and Stieger, K. (2015). Functional Characterization of AAV-Expressed Recombinant Anti-VEGF Single-Chain Variable Fragments In Vitro. J. Ocul. Pharmacol. Therapeut. *31*, 269–276. https://doi.org/10.1089/jop.2014.0125.

43. Khanani, A.M., Boyer, D.S., Wykoff, C.C., Regillo, C.D., Busbee, B.G., Pieramici, D., Danzig, C.J., Joondeph, B.C., Major, J.C., Jr., Turpcu, A., and Kiss, S. (2024). Safety and efficacy of ixoberogene soroparvovec in neovascular age-related macular degeneration in the United States (OPTIC): a prospective, two-year, multicentre phase 1 study. eClinicalMedicine *67*, 102394. https://doi.org/10.1016/j.eclinm.2023.102394.

44. Lochrie, M.A., Tatsuno, G.P., Christie, B., McDonnell, J.W., Zhou, S., Surosky, R., Pierce, G.F., and Colosi, P. (2006). Mutations on the external surfaces of adeno-associated virus type 2 capsids that affect transduction and neutralization. J. Virol. *80*, 821–834. https://doi.org/10.1128/jvi.80.2.821-834.2006.

45. O'Donnell, J., Taylor, K.A., and Chapman, M.S. (2009). Adeno-associated virus-2 and its primary cellular receptor—Cryo-EM structure of a heparin complex. Virology *385*, 434–443. https://doi.org/10.1016/j.virol.2008.11.037.

46. Nieuwenhuis, B., Laperrousaz, E., Tribble, J.R., Verhaagen, J., Fawcett, J.W., Martin, K.R., Williams, P.A., and Osborne, A. (2023). Improving adeno-associated viral (AAV) vector-mediated transgene expression in retinal ganglion cells: comparison of five promoters. Gene Ther. *30*, 503–519. https://doi.org/10.1038/s41434-022-00380-z.

47. Hartl, D., Krebs, A.R., Grand, R.S., Baubec, T., Isbel, L., Wirbelauer, C., Burger, L., and Schübeler, D. (2019). CG dinucleotides enhance promoter activity independent of DNA methylation. Genome Res. *29*, 554–563. https://doi.org/10.1101/gr.241653.118.

48. Ma, Y., Budde, M.W., Zhu, J., and Elowitz, M.B. (2023). Tuning Methylation-Dependent Silencing Dynamics by Synthetic Modulation of CpG Density. ACS Synth. Biol. *12*, 2536–2545. https://doi.org/10.1021/acssynbio.3c00078.

49. Cabrera, A., Edelstein, H.I., Glykofrydis, F., Love, K.S., Palacios, S., Tycko, J., Zhang, M., Lensch, S., Shields, C.E., Livingston, M., et al. (2022). The sound of silence: Transgene silencing in mammalian cell engineering. Cell Syst. *13*, 950–973. https://doi.org/10.1016/j.cels.2022.11.005.

50. Kia, A., Yata, T., Hajji, N., and Hajitou, A. (2013). Inhibition of histone deacetylation and DNA methylation improves gene expression mediated by the adeno-associated virus/phage in cancer cells. Viruses *5*, 2561–2572. https://doi.org/10.3390/v5102561.

51. Herbst, F., Ball, C.R., Tuorto, F., Nowrouzi, A., Wang, W., Zavidij, O., Dieter, S.M., Fessler, S., van der Hoeven, F., Kloz, U., et al. (2012). Extensive Methylation of Promoter Sequences Silences Lentiviral Transgene Expression During Stem Cell

Differentiation In Vivo. Mol. Ther. *20*, 1014–1021. https://doi.org/10.1038/mt.2012.46.

52. Marx, N., Dhiman, H., Schmieder, V., Freire, C.M., Nguyen, L.N., Klanert, G., and Borth, N. (2021). Enhanced targeted DNA methylation of the CMV and endogenous promoters with dCas9-DNMT3A3L entails distinct subsequent histone modification changes in CHO cells. Metab. Eng. *66*, 268–282. https://doi.org/10.1016/j.ymben.2021.04.014.

53. Brown, H.C., Zakas, P.M., George, S.N., Parker, E.T., Spencer, H.T., and Doering, C.B. (2018). Target-Cell-Directed Bioengineering Approaches for Gene Therapy of Hemophilia A. Mol. Ther. Methods Clin. Dev. *9*, 57–69. https://doi.org/10.1016/j.omtm.2018.01.004.

54. Xie, Q., Chen, X., Ma, H., Zhu, Y., Ma, Y., Jalinous, L., Cox, G.F., Weaver, F., Yang, J., Kennedy, Z., et al. (2024). Improved gene therapy for spinal muscular atrophy in mice using codon-optimized hSMN1 transgene and hSMN1 gene-derived promotor. EMBO Mol. Med. *16*, 945–965. https://doi.org/10.1038/s44321-024-00037-x.

55. Sahni, J., Patel, S.S., Dugel, P.U., Khanani, A.M., Jhaveri, C.D., Wykoff, C.C., Hershberger, V.S., Pauly-Evers, M., Sadikhov, S., Szczesny, P., et al. (2019). Simultaneous Inhibition of Angiopoietin-2 and Vascular Endothelial Growth Factor-A with Faricimab in Diabetic Macular Edema: BOULEVARD Phase 2 Randomized Trial. Ophthalmology *126*, 1155–1170. https://doi.org/10.1016/j.ophtha.2019.03.023.

56. Heier, J.S., Kherani, S., Desai, S., Dugel, P., Kaushal, S., Cheng, S.H., Delacono, C., Purvis, A., Richards, S., Le-Halpere, A., et al. (2017). Intravitreous injection of AAV2-sFLT01 in patients with advanced neovascular age-related macular degeneration: a phase 1, open-label trial. Lancet *390*, 50–61. https://doi.org/10.1016/S0140-6736(17)30979-0.

57. Constable, I.J., Pierce, C.M., Lai, C.M., Magno, A.L., Degli-Esposti, M.A., French, M.A., McAllister, I.L., Butler, S., Barone, S.B., Schwartz, S.D., et al. (2016). Phase 2a Randomized Clinical Trial: Safety and Post Hoc Analysis of Subretinal rAAV.sFLT-1 for Wet Age-related Macular Degeneration. EBioMedicine *14*, 168–175. https://doi.org/10.1016/j.ebiom.2016.11.016.

58. Maguire, A.M., Simonelli, F., Pierce, E.A., Pugh, E.N., Mingozzi, F., Bennicelli, J., Banfi, S., Marshall, K.A., Testa, F., Surace, E.M., et al. (2008). Safety and Efficacy of Gene Transfer for Leber's Congenital Amaurosis. N. Engl. J. Med. Overseas. Ed. *358*, 2240–2248. https://doi.org/10.1056/NEJMoa0802315.

59. Rogers, D., and Hahn, M. (2010). Extended-connectivity fingerprints. J. Chem. Inf. Model. *50*, 742–754. https://doi.org/10.1021/ci100050t.

60. Deb, K., Pratap, A., Agarwal, S., and Meyarivan, T. (2002). A fast and elitist multiobjective genetic algorithm: NSGA-II. IEEE Trans. Evol. Comput. *6*, 182–197. https://doi.org/10.1109/4235.996017.

61. Fortin, F.-A., Rainville, F.-M.D., Gardner, M.-A.G., Parizeau, M., and Gagné, C. (2012). DEAP: evolutionary algorithms made easy. J. Mach. Learn. Res. *13*, 2171–2175.

62. Wolf, F.A., Angerer, P., and Theis, F.J. (2018). SCANPY: large-scale single-cell gene expression data analysis. Genome Biol. *19*, 15. https://doi.org/10.1186/s13059-017-1382-0.

63. Macosko, E.Z., Basu, A., Satija, R., Nemesh, J., Shekhar, K., Goldman, M., Tirosh, I., Bialas, A.R., Kamitaki, N., Martersteck, E.M., et al. (2015). Highly Parallel Genome-wide Expression Profiling of Individual Cells Using Nanoliter Droplets. Cell *161*, 1202–1214. https://doi.org/10.1016/j.cell.2015.05.002.

64. Peng, Y.R., Shekhar, K., Yan, W., Herrmann, D., Sappington, A., Bryman, G.S., van Zyl, T., Do, M.T.H., Regev, A., and Sanes, J.R. (2019). Molecular Classification and Comparative Taxonomics of Foveal and Peripheral Cells in Primate Retina. Cell *176*, 1222–1237.e22. https://doi.org/10.1016/j.cell.2019.01.004.

65. Zhang, X., Lan, Y., Xu, J., Quan, F., Zhao, E., Deng, C., Luo, T., Xu, L., Liao, G., Yan, M., et al. (2019). CellMarker: a manually curated resource of cell markers in human and mouse. Nucleic Acids Res. *47*, D721–D728. https://doi.org/10.1093/nar/gky900.

66. Mowat, F.M., Gornik, K.R., Dinculescu, A., Boye, S.L., Hauswirth, W.W., Petersen-Jones, S.M., and Bartoe, J.T. (2014). Tyrosine capsid-mutant AAV vectors for gene delivery to the canine retina from a subretinal or intravitreal approach. Gene Ther. *21*, 96–105. https://doi.org/10.1038/gt.2013.64.

OMTA, Volume *34*

Supplemental information

AI-engineered AAV capsid enables intravitreal delivery for the treatment of diverse retinal degenerations

Mochen Cui, Huaqing Liu, Lei Cai, Qian Zhang, Li Yuan, Cui Gao, Chunlian Li, Jianfei Xi, Yongkui Li, Chenguang Wu, Qin Zheng, Lei Liu, Peiyi Chen, Shuxian Zhou, Xing Mou, Joseph Wekselblatt, Yu Zhang, Lance Han, and Sheng Ren

Table S1. Key primer table.

Name	Sequence	Targeted Element
AAV2 Cap Insertion F	GACAACCAATCCCGTGGCTA	AAV Library
AAV2 Cap Insertion R	AGACCATGCCTGGAAGAACG	
AAV2 Barcoded eGFP F	AATGATACGGCGACCACCGAGATCTACACTCTTTCCCTACACGACGCTCT	Barcoded eGFP
AAV2 Barcoded eGFP R	CAAGCAGAAGACGGCATACGAGATATCACGTTGTGACTGGAGTTCAGACGT GTGCTCTTCCGATCTCACATGGTCCTGCTGGAGTT	
AAV2 eGFP F (Titer)	GCTACCCCGACCACATGAAG	eGFP
AAV2 eGFP R (Titer)	CGGGTCTTGTAGTTGCCGT	
AAV2 LUC F	TGGGCGCGTTATTTATCGGA	Luciferase
AAV2 LUC R	CACTACGGTAGGCTGCGAAA	
hGUCY2D-129 F	CCAAACAACATCTGCGGTGG	*hGUCY2D*
hGUCY2D-129 R	TTGCATGTGAAGTAGCCGGT	
hGUCY2D-129 VIC	CCCATCCCAACCACCAGGAGGAAGCCA	
Anti-VEGFA 180 F	GGTGACGGTGTCATGGAACT	Anti-VEGFA
Anti-VEGFA 180 FAM	CCTCAGCAGCGTGGTGACCGTGCC	
Anti-VEGFA 180 R	TTGTCCACCTTGGTGTTGCT	
hVEGFA-123 F	CTTGCCTTGCTGCTCTACCT	*hVEGFA*
hVEGFA-123 R	GCAGTAGCTGCGCTGATAGA	
mVegfa-195 F	CACGACAGAAGGAGAGCAGA	m*Vegfa*
mVegfa-195 R	CACTCCAGGGCTTCATCGTT	
mGapdh F	AGGTCGGTGTGAACGGATTTG	m*Gapdh*
mGapdh R	TGTAGACCATGTAGTTGAGGTCA	

Table S2. Insertion sequences for monkey-targeting.

Capsid ID	Insertion Sequence
PN168	DEEEIRTTNPVATEQYGSVSTNLQRGNAAVRFDGTERAAR
PN169	DEEEIRTTNPVATEQYGSVSTNLQRGNRQYSDAVRAER
PN170	SEEEIRTTNPSATEQYGVAATNLQKGNR
PN171	DEEEIRTTNPVATEQYGSVSTNLQRGNAAILGTVKHAR
PN172	DEEEIRTTNPVATEQYGSVSTNLQRGNAADRETGRPAR
PN173	DEEEIRTTNPVATEQYGSVSTNLQRGNAALNSMAKPAR
PN174	DEEEIRTTNPVATEQYGSVSTNLQRGNTARDGVEGSRAAR
PN175	DEEEIRTTNPVATEMYGEVSRNLQRGER
PN176	DEEEIRTTNPVAYEPYGSVSTNLQRGNQAEREYNRPAR
PN177	DEEEIRTTNPVATEQYGSVSTNLQRGNYADSSSGRPAR
PN178	DEEEIRTTNPVATEQYGSVSTNLQRGNLANYETPRPAR
PN179	DEEEIRTTNPVATEQYGEVSRNLQRGER
PN180	DEEEIRTTNPVATEQYGSVSTNLQRGNAANAMLGRPAR
PN181	DEEEIRTTNPVATEQYGSVSTNLQRGNAAHPEVGRPAR
PN182	DEEEIRTTNPVATEQYGSVSTNLQRGNAAHNEQMRPGR
PN183	DEEEIRTTNPVATEQYGSVSTNLQRGNTATEDRSRPAR
PN184	DEEEIRTTNPVATEQYGSVSTNLQRGNSALNATSRPAR
PN185	DEEEIRTTNPVATEQYGYVSTNVQRTGR
PN186	DEEEIRTTNPVATEQYGSVSTNLQRGNAAPGVNTKPAR
PN187	DEEEIRTTNPVATEQYGSVSTNLQRGNRNAGEDHSLRAAR
PN188	DEQEIATTNPVATEQYGVVSNNLQRTNR
PN189	DEEEIRTTNPVATEQYGSVSTNLQRGNATAPNSVARPAR
PN190	DEEEIRTTNPVATEQYGSVSTNLQRGNQTINSAPRPAR
PN191	DEEEIRTTNPVATEQYGSVSTNLQRQTR
PN192	DEEEIRTTNPVATEQYGSVSTNLQRGNAAVRFNGTERAAR
PN193	DEEEIRTTNPVATEQYGSVSTNLQRGNAIHPEVGRPAR
PN194	DEEEIRTTNPVATEQYGSVSTNLQRGNAASLSPTRAAR
PN195	DEEEIRTTNPVATEQYGYVSTNVQRGGR
PN196	DEEEIRTTNPVATEQYGSVSTNLQRGNTAQDGQGRPAR
PN197	SEEEIRTTNPSATEQYGSVSTNLQRGNR
PN198	DEEEIRTTNPVATEQYGSVSTNLQRGNAATLSSTVPAR
PN199	DEEEIRTTNPVATEQYGSVSTNLQRGNLADTDKFRQAR
PN200	DEEEIRTTNPVATEQYGSVSTNLQRGNTGSTPSRSGPGLSR
PN201	DEEEIRTTNPVATEQYGSVSTNLQRGNTAQDGTSRRER
PN202	DEEEIRTTNPVATEQYGSVSTNLQRGNAAPNSVARPAR
PN203	DEEEIRTTNPVATEQYGSVSTNLQRGNAAHNDTPRPAR
PN204	DEEEIRTTNPVATEQYGSVSTNLQRGNAAANSPTTQRAAR
PN205	DEEEIRTTNPVATEQYGSVSTNLQRGNLAHNEFSRPAR
PN206	DEEKIRTTNPVATEQYGSVSTNLQRGNR
PN207	DEEEIRTTNPVATEQYGSVSSNLQRGNAAHNDTPRPAR

Table S3. Insertion sequences for mouse-targeting.

Capsid ID	Insertion Sequence
PM241	TGRDRLGLSGLS
PM242	TGTGLTIGRGLS
PM243	TGTSTFGASRGLS
PM244	TGTGAFAQRGLS
PM245	TGGSYLPSPSRGLS
PM246	TGFSLPGSSRGLS
PM247	TGGASYGLPQSRGLS
PM248	TGSQFAPARGLS
PM249	TGSRQAYSTGLS
PM250	TGGLLVSSRGLS
PM251	TGTVSGIGRGLS
PM252	TGSSLLGSSSRGLS
PM253	TGGMVSGLSRGLS
PM254	TGSQLLGSRGLS
PM255	TGLSTLGNRGLS
PM256	TGYSSLPSGSRGLS
PM257	TGRPQAYSSGLS
PM258	TGTTFTAQRGLS
PM259	TGQTLGMSRGLS
PM260	TGGAAGLSRGLS
PM261	TGSLLQLSGSRGLS
PM262	TGQGPLSSRGLS
PM263	TGFSPLTPGRGLS
PM264	TGGTLSISRGLS
PM265	TGQLGSLSRGLS
PM266	TGGSFTGVGRGLS
PM267	TGANILGSRGLS
PM268	TGSRTGFSGGLS
PM269	TGSSSLLGTSRGLS
PM270	TGNPLLQTSRGLS
PM271	TGPSSSPFTGRGLS
PM272	TGYLPTGQSTRGLS
PM273	TGGTMLGTRGLS
PM274	TGSGAVPSLHRGLS
PM275	TGSLLGVGSRGLS
PM276	TGRGAGYPSGLS
PM277	TGLTTGLSRGLS
PM278	TGSTIGLGSRGLS
PM279	TGSSSFTPSRGLS
PM280	TGSMLLSSGRGLS

PM281	TGSQFGQPMSRGLS
PM282	TGVSALGGMRGLS
PM283	TGASQAFPRGLS
PM284	TGGGMATGLSRGLS
PM285	TGGLLSATRGLS
PM286	TGVTPTGSRGLS
PM287	TGTAIGGSRGLS
PM288	TGHTIGPIPGRGLS
PM289	TGSIAGGSLRGLS
PM290	TGGALSLSSRGLS
PM054	TGTSTFGASRGLS

Table S4. Key resource table.

Reagent or Resource	Source/Vendor	Identifier
**Plasmid Construction and AAV Packaging**		
NheI-HF	New England Biolabs	Cat# R3131
NotI-HF	New England Biolabs	Cat# R3189
pAAV2/2	Addgene	Cat# Plasmid #104963
pAAV CAGG eGFP	Addgene	Cat# Plasmid #107707
BsrGI-HF	New England Biolabs	Cat# R3575
XhoI	New England Biolabs	Cat# R0146
Double-stranded DNA Fragments Containing Barcodes	Integrated DNA Technologies	N/A
Gibson Assembly Master Mix	New England Biolabs	Cat# E2611
EcoRI-HF	New England Biolabs	Cat# R3101
BglII	New England Biolabs	Cat# R0144
Double-stranded DNA Fragments Containing Luciferase cDNA	Integrated DNA Technologies	N/A
Double-stranded DNA fragments containing anti-VEGFA cDNA	Integrated DNA Technologies	N/A
PEI MAX - Transfection Grade Linear Polyethylenimine Hydrochloride	Polysciences	Cat# 24765
pAdDeltaF6	Addgene	Cat# Plasmid #112867
Benzonase Nuclease	Merck Millipore	Cat# 70746
Amicon Ultra Centrifugal Filter, 100 kDa MWCO	Merck Millipore	Cat# UFC8100
**Nucleic Acid Isolation, Amplification and Assessment**		
Q5 High-Fidelity DNA Polymerase	New England Biolabs	Cat# M0491
QIAquick PCR Purification Kit for PCR Cleanup	Qiagen	Cat# 28104
TRIzol Reagent	Invitrogen	Cat# 15596018CN
RNeasy Mini Kit	Qiagen	Cat# 74104
DNA-free DNA Removal Kit	Invitrogen	Cat# AM1906
RevertAid First Strand cDNA Synthesis Kit	Thermo Scientific	Cat# K1621
10x Chromium Single Cell 3’ kits (multiple components)	10x Genomics	Cat# 1000075<br>Cat# 1000092<br>Cat# 1000073<br>Cat# 1000074<br>Cat# 120262
SPRIselect Bead-Based Reagent	Beckman Coulter Diagnostics	Cat# B23318
TaqMan Fast Advanced Master Mix	Applied Biosystems	Cat# 4444556
Digital PCR Buffer	RainSure Scientific	Cat# 3005
Transduction Inhibition Rate		
Dulbecco’s Modified Eagle’s Medium	Corning	Cat# 10-013-CVR
Fetal Bovine Serum, Premium	Gibco	Cat# A5670701
Trypsin (2.5%), No Phenol Red	Gibco	Cat# 15090046
**Heparin Binding Assay**		
Heparin sodium salt from porcine intestinal mucosa	Sigma-Aldrich	Cat# H3149
TrypLE™ Express Enzyme (1X)	Gibco	Cat# 12604039
***in vivo* Procedures**		
Small Hub Removable Needle, 33-gauge	Hamilton Company	Cat# 7803-05
2.5 µl Microliter Syringe	Hamilton Company	Cat# 7632-01
BD Ultra-Fine U-100 Syringe	Becton Dickinson	Cat# BD 328291
AK-FLUOR (Fluorescein Injection)	Akorn	Cat# 17478-253-10
D-Luciferin, Sodium Salt	Yeasen	Cat# 40901ES03
Ranibizumab (Lucentis)	Novartis Pharma Schweiz AG	Cas ID 347396-82-1
**Immunostaining**		
Tissue-Tek O.C.T. Compound	Sakura Finetek	Cat# 4583

SuperFrost Ultra Plus GOLD Adhesion Slides	Epredia	Cat# K5800AMNZ72
Bovine Serum Albumin, Lyophilized Powder	Sigma-Aldrich	Cat# A9418
GFP Polyclonal Antibody, Alexa Fluor 488	Invitrogen	Cat# A-21311
DAPI	Thermo Scientific	Cat# 62248
ProLong Gold Antifade Mountant	Invitrogen	Cat# P36930
**Experimental Models: Cells/Animals**		
Cell: HEK293T	ATCC	Cat# CRL-3216
Mouse: *C57BL/6J*	Charles River	RRID: IMSR_JAX:000664
Mouse: *BALB/cJ*	Charles River	RRID: IMSR_JAX:000651
Mouse: *C57BL/6JCya-Gucy2e^{em1}Gucy2f^{em1}/Cya*	Cyagen	Cat# C001928
Mouse: *C57BL/6JCya-Tg(bRho-VEGFA)/Cya*	Cyagen	Cat# C001395
Monkey: Cynomolgus Monkey (*Macaca fascicularis*)	Landao-Bio	N/A
**Software and Algorithms**		
Python 3.0	Public Domain	https://www.python.org/
Scanpy	Public Domain	https://github.com/scverse/scanpy
RDKit	Public Domain	https://www.rdkit.org/docs/index.html
NovoExpress 1.5.0	Agilent Technologies	https://www.agilent.com.cn/en/product/research-flow-cytometry/flow-cytometry-software/novocyte-novoexpress-software-1320805
cellSens 3.2	Olympus	N/A
LAS X 3.10.0	Leica	https://www.leica-microsystems.com/products/microscope-software/p/leica-las-x-ls/
LAS X 4.7.0	Leica	https://www.leica-microsystems.com/products/microscope-software/p/leica-las-x-ls/
AniView 1.00.00621	BLT-Imaging	N/A
Imaris 10	Bitplane	https://imaris.oxinst.com
ImageJ 1.54f	Public Domain	http://imagej.org
GeneCount Analysis System software v1.63.0222	RainSure Scientific	N/A
Image-Pro Plus 6.0	Media Cybernetics	https://mediacy.com/image-pro/
Prism 10	GraphPad Software	https://www.graphpad.com/scientific-software/prism/

Figure S1

A

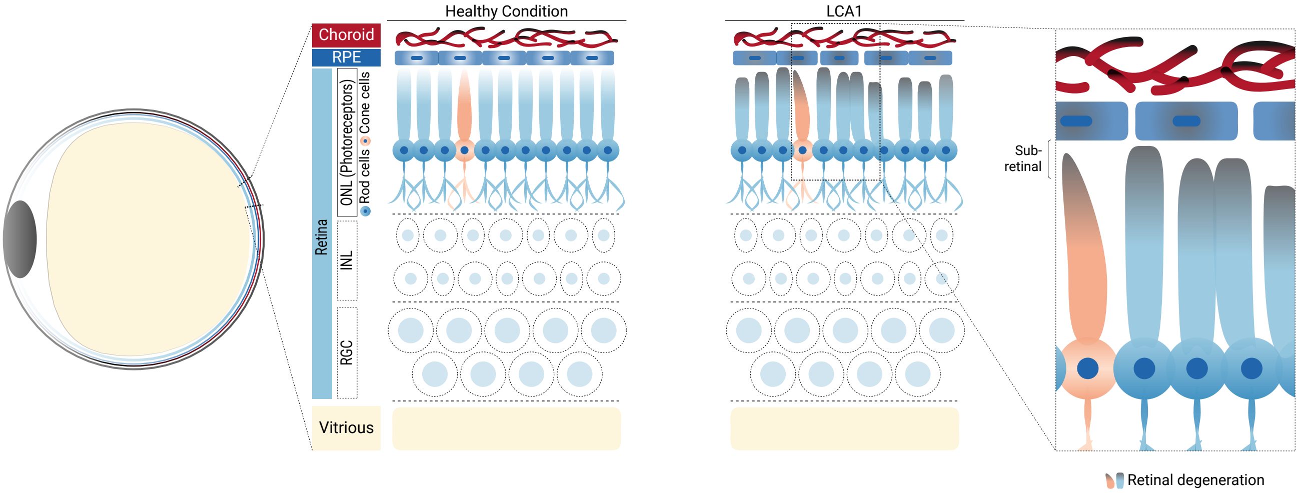
Healthy Condition
LCA1
Choroid
RPE
Retina
ONL (Photoreceptors)
Rod cells
Cone cells
INL
RGC
Vitrious
Sub-retinal
Retinal degeneration

B

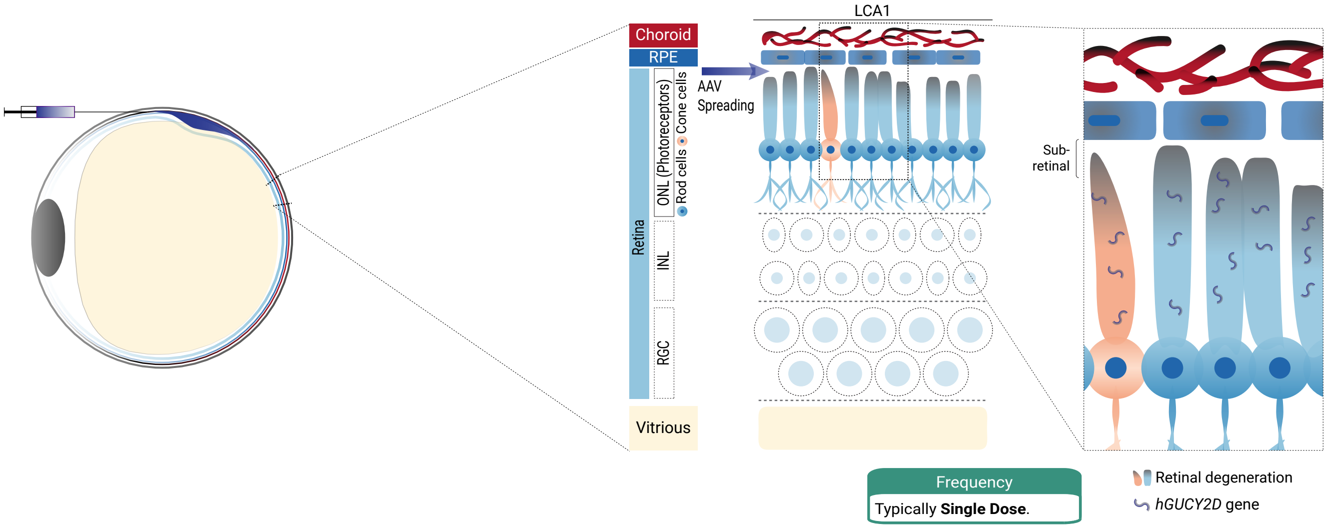
LCA1
Choroid
RPE
AAV Spreading
Retina
ONL (Photoreceptors)
Rod cells
Cone cells
INL
RGC
Vitrious
Sub-retinal
Frequency
Typically Single Dose.
Retinal degeneration
hGUCY2D gene

Figure S1. Schematic representation of LCA1 pathology and AAV-mediated hGUCY2D therapy for LCA1. A, Schematic representation of LCA1. **B**, Schematic representation of AAV-mediated hGUCY2D therapy for LCA1. RGC, retinal ganglion cells; INL, inner nuclear layer; ONL, outer nuclear layer; RPE, retinal pigment epithelium; LCA1, Leber's congenital amaurosis type 1.

Figure S2

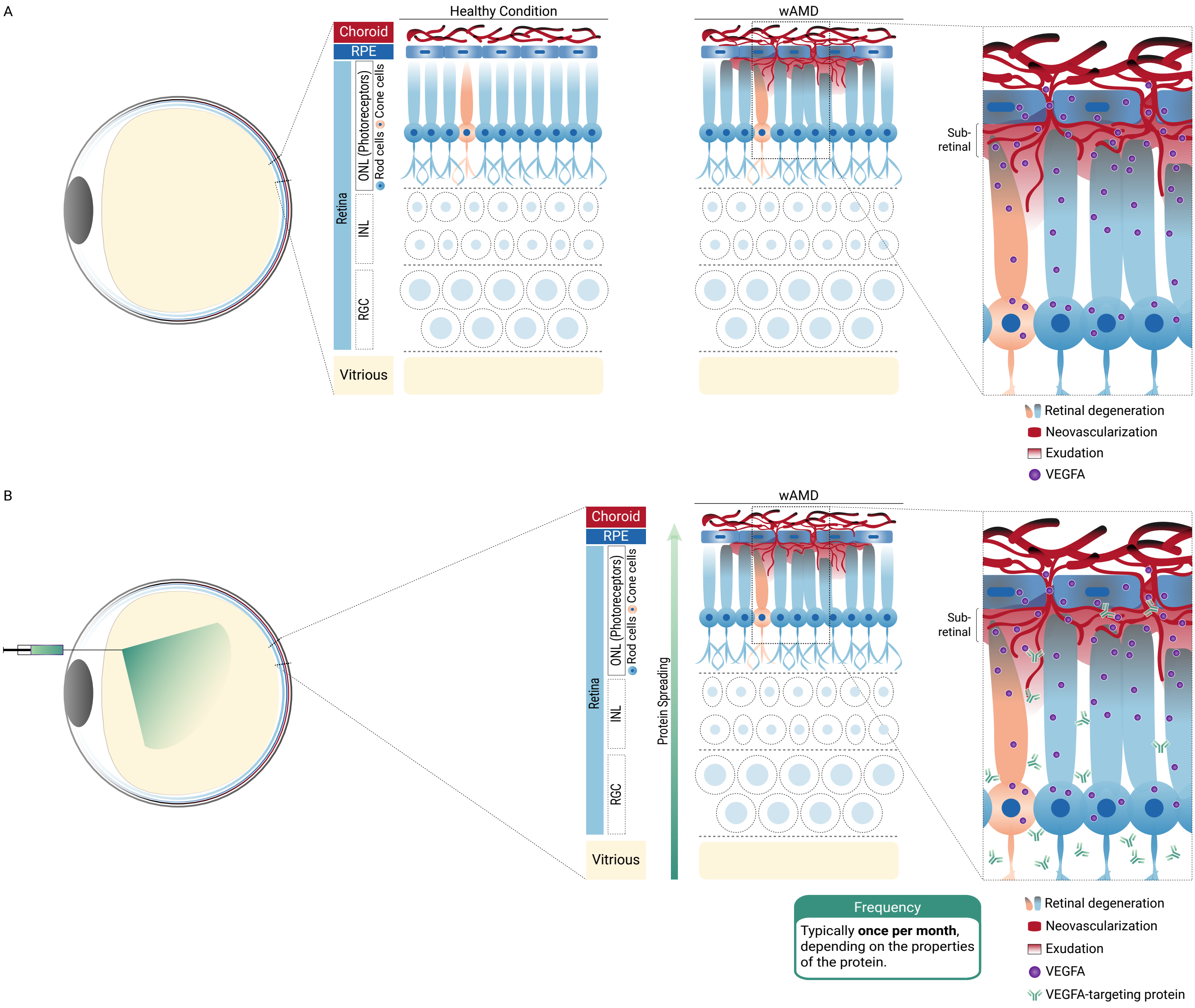
A
Healthy Condition
wAMD
Choroid
RPE
Retina
ONL (Photoreceptors)
Rod cells
Cone cells
INL
RGC
Vitrious
Sub-retinal
Retinal degeneration
Neovascularization
Exudation
VEGFA
B
Protein Spreading
Frequency
Typically **once per month**, depending on the properties of the protein.
VEGFA-targeting protein

Figure S2. Schematic representation of wAMD pathology and anti-VEGFA protein drug therapy for wAMD. A, Schematic representation of wAMD. **B**, Schematic representation of anti-VEGFA therapy for wAMD with protein drugs. RGC, retinal ganglion cells; INL, inner nuclear layer; ONL, outer nuclear layer; RPE, retinal pigment epithelium; wAMD, wet age-related macular degeneration.

Figure S3

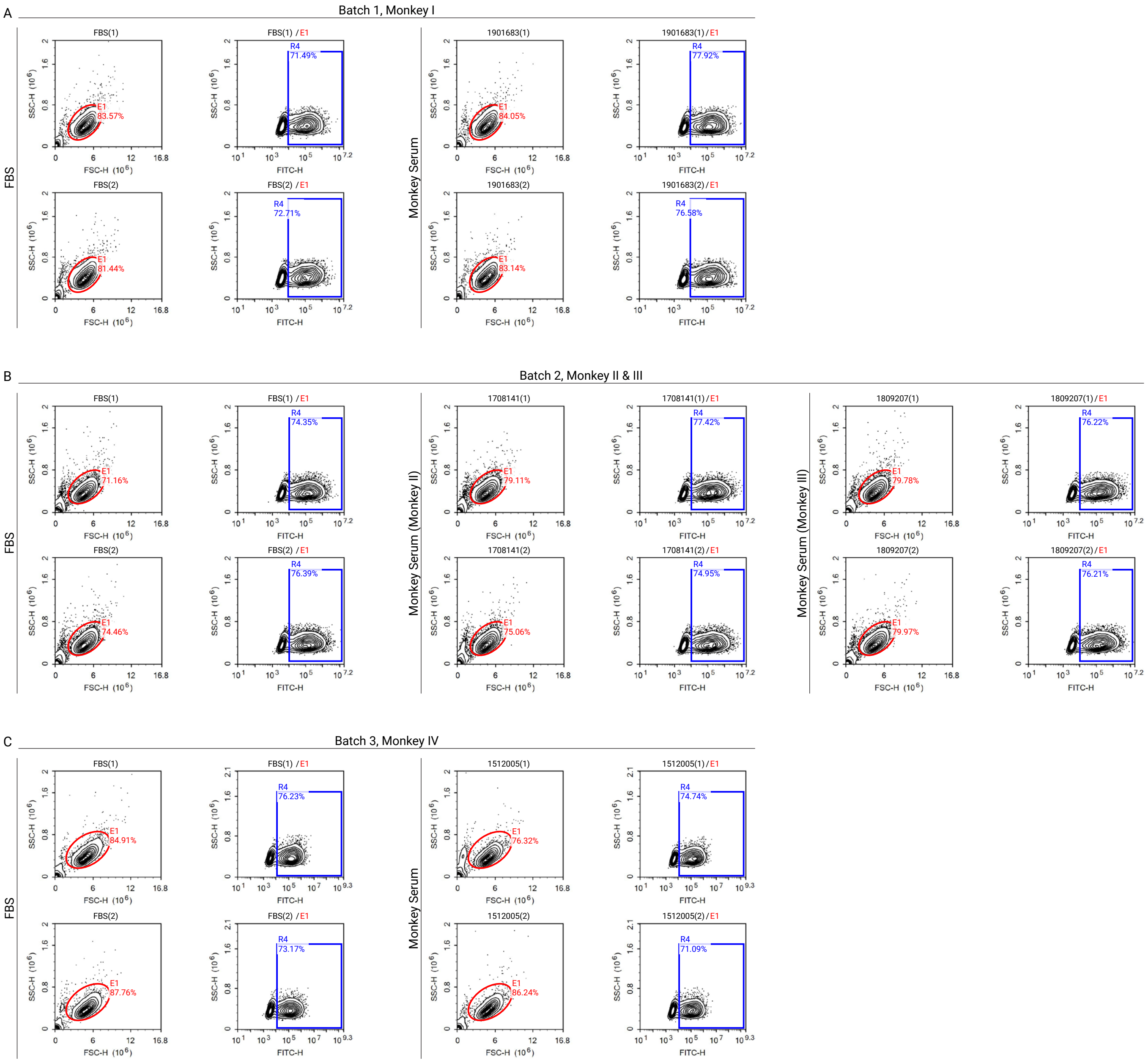
A
Batch 1, Monkey I
FBS
FBS(1)
E1
83.57%
FBS(1) / E1
R4
71.49%
FBS(2)
E1
81.44%
FBS(2) / E1
R4
72.71%
Monkey Serum
1901683(1)
E1
84.05%
1901683(1) / E1
R4
77.92%
1901683(2)
E1
83.14%
1901683(2) / E1
R4
76.58%
SSC-H (10^6)
FSC-H (10^6)
FITC-H
B
Batch 2, Monkey II & III
FBS
FBS(1)
E1
71.16%
FBS(1) / E1
R4
74.35%
FBS(2)
E1
74.46%
FBS(2) / E1
R4
76.39%
Monkey Serum (Monkey II)
1708141(1)
E1
79.11%
1708141(1) / E1
R4
77.42%
1708141(2)
E1
75.06%
1708141(2) / E1
R4
74.95%
Monkey Serum (Monkey III)
1809207(1)
E1
79.78%
1809207(1) / E1
R4
76.22%
1809207(2)
E1
79.97%
1809207(2) / E1
R4
76.21%
C
Batch 3, Monkey IV
FBS
FBS(1)
E1
84.91%
FBS(1) / E1
R4
76.23%
FBS(2)
E1
87.76%
FBS(2) / E1
R4
73.17%
Monkey Serum
1512005(1)
E1
76.32%
1512005(1) / E1
R4
74.74%
1512005(2)
E1
86.24%
1512005(2) / E1
R4
71.09%

Figure S3. FACS analysis of transduction inhibition rate on AAV2.WT. A The population of GFP^+ cells (R4) after incubation with eGFP-encoding AAV2 pretreated with FBS and monkey serum from monkey I. **B**, The population of GFP^+ cells (R4) was examined after incubation with eGFP-encoding AAV2 pretreated with FBS and monkey serum from monkeys II and III. Notably, the FBS group was utilized for both monkeys since their samples were detected within the same batch. **C**, The population of GFP^+ cells (R4) after incubation with eGFP-encoding AAV2 pretreated with FBS and monkey serum from monkey IV. Two serum samples from each monkey were included, along with corresponding controls (FBS). The order is indicated in parentheses next to the panel title.

Figure S4

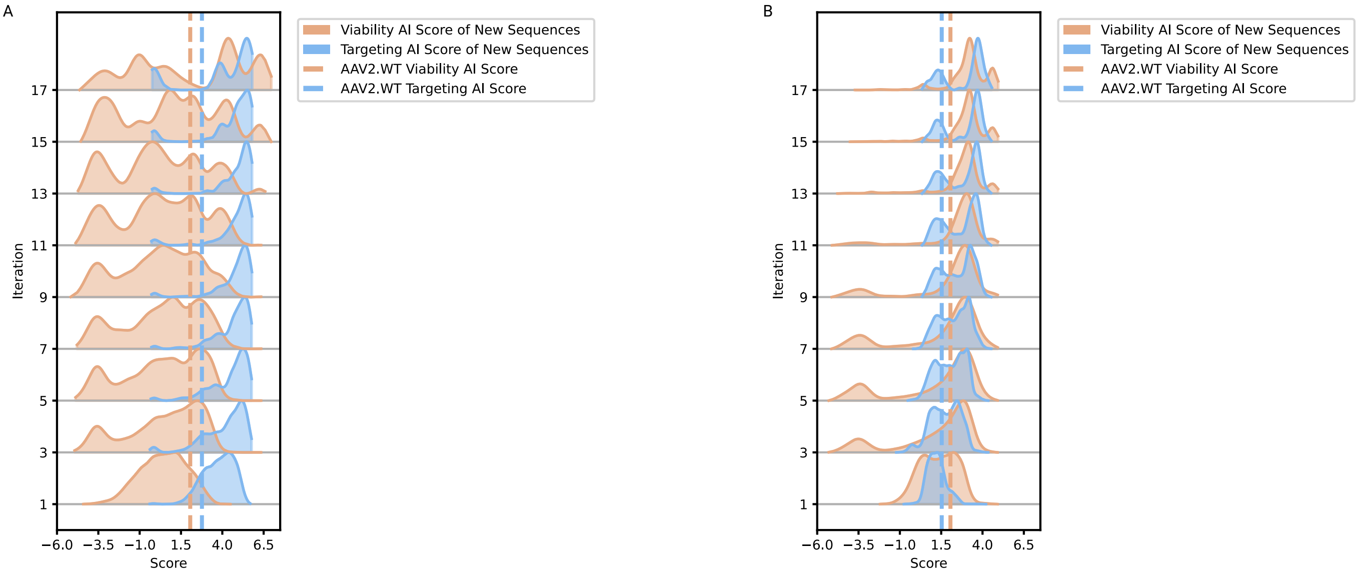

Mouse Viability and Targeting Models

Monkey Viability and Targeting Models

Figure S4. Iterative process for new sequence generation. A, Iteration of the mouse framework. **B**, Iteration of the monkey framework.

Figure S5

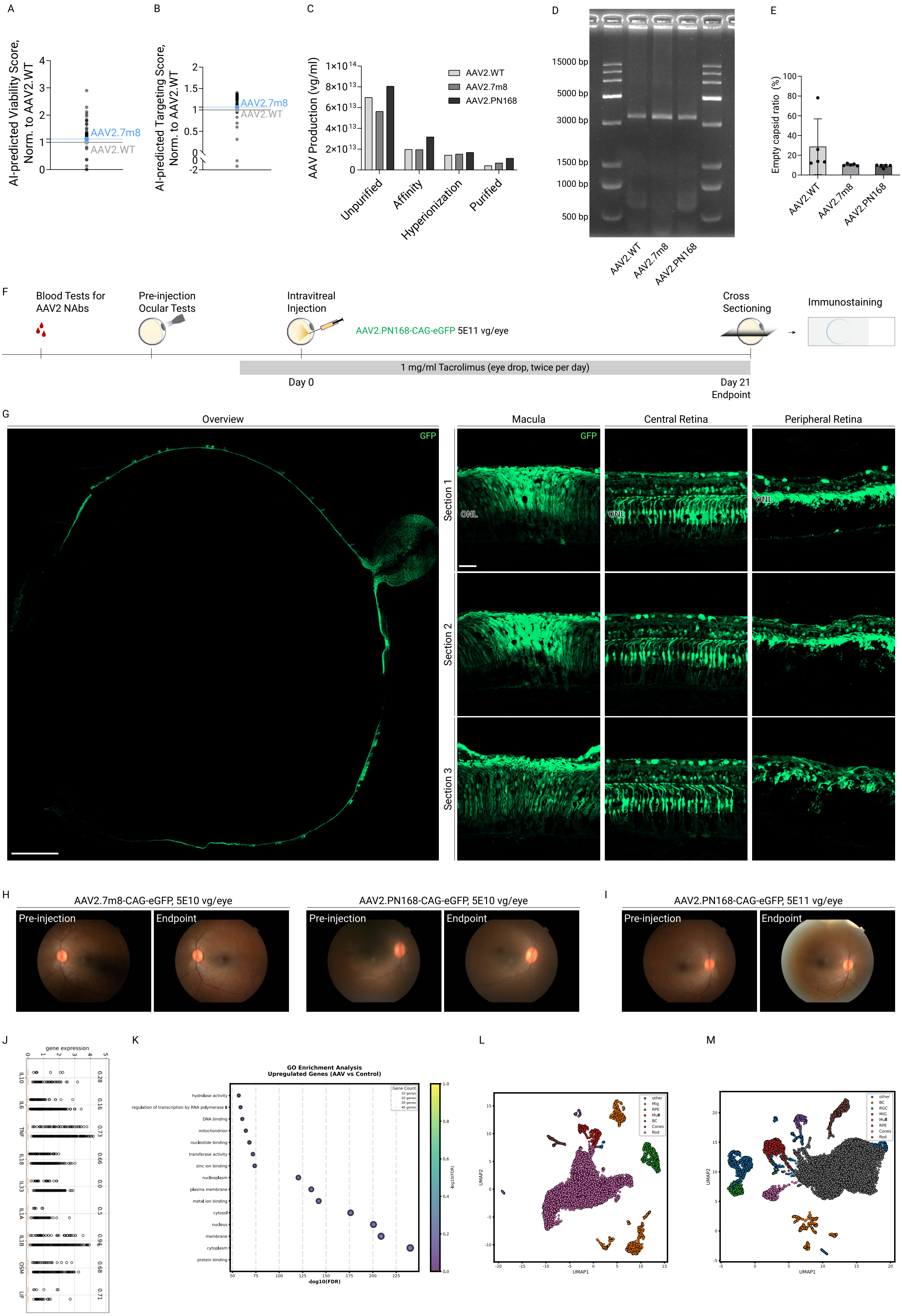
A
AI-predicted Viability Score, Norm. to AAV2.WT
AAV2.7m8
AAV2.WT
B
AI-predicted Targeting Score, Norm. to AAV2.WT
AAV2.7m8
AAV2.WT
C
AAV Production (vg/ml)
AAV2.WT
AAV2.7m8
AAV2.PN168
Unpurified
Affinity
Hyperionization
Purified
D
15000 bp
5000 bp
3000 bp
1500 bp
1000 bp
500 bp
AAV2.WT
AAV2.7m8
AAV2.PN168
E
Empty capsid ratio (%)
AAV2.WT
AAV2.7m8
AAV2.PN168
F
Blood Tests for AAV2 NAbs
Pre-injection Ocular Tests
Intravitreal Injection
AAV2.PN168-CAG-eGFP 5E11 vg/eye
Cross Sectioning
Immunostaining
1 mg/ml Tacrolimus (eye drop, twice per day)
Day 0
Day 21 Endpoint
G
Overview
Macula
Central Retina
Peripheral Retina
GFP
Section 1
Section 2
Section 3
ONL
H
AAV2.7m8-CAG-eGFP, 5E10 vg/eye
AAV2.PN168-CAG-eGFP, 5E10 vg/eye
I
AAV2.PN168-CAG-eGFP, 5E11 vg/eye
Pre-injection
Endpoint
J
gene expression
IL10
IL6
TNF
IL18
IL33
IL1A
IL1B
OSM
LIF
K
GO Enrichment Analysis
Upregulated Genes (AAV vs Control)
hydrolase activity
regulation of transcription by RNA polymerase II
DNA binding
mitochondrion
nucleotide binding
transferase activity
zinc ion binding
nucleoplasm
plasma membrane
metal ion binding
cytosol
nucleus
membrane
cytoplasm
protein binding
-log10(FDR)
Gene Count
L
UMAP1
UMAP2
other
Mig
RPE
Mull
BC
Cones
Rod
M
UMAP1
UMAP2
other
BC
RGC
MiG
Mull
RPE
Cones
Rod

Figure S5. Performance of AAV2.PN168 in AI-prediction, production, and intravitreal injection at various doses. A, AI-predicted viability score of the 43 AI-generated AAV2-derived variants, normalized to AAV2.WT, semi-transparent grey dots indicate AI-generated capsids, solid grey dot indicates AAV2.WT, solid blue dot indicates AAV2.7m8. **B**, AAV production at different stages. **C**, Gel electrophoresis of lysed AAVs, showing bands for AAV-packaged barcoded eGFP. **D**, Empty capsid ratio of purified AAVs. **F**, Experimental design. **G**, Representative confocal micrographs of total retinal cross sections and different retinal regions from monkey eyes receiving different high-dose AAV2.PN168. ONL is indicated in the graphs. **H**&**I**, Fundoscopy of the monkey eyes, before and after the injection of corresponding AAVs and dosages. **J**, Comparison of gene expression across immune-associated factors between AAV-transduced and non-transduced retinal cells, showing no significant differences. **K**, Gene Ontology enrichment analysis for both AAV-transduced and non-transduced retinal cells, showing no upregulation of immune-associated pathways. **L**, UMAP plot showing monkey retinal cells. **M**. UMAP plot depicting mouse retinal cells. n = 5 regions from EM (**E**), n = 1 eye (**F**-**H**), 7,742 retinal cells were analyzed (**J**-**L)**, 18,577 retinal cells were analyzed (**M**). Dot plots show mean (SD). Scale bars: **i** = 2000 μm (overview), 50 μm (individual retinal regions). For fundus images, all images were obtained using a fundus camera at a fixed magnification and focal length, ensuring comparable coverage and locations across different groups, no scale was indicated by the imaging system. ONL, outer nuclear layer.

Figure S6

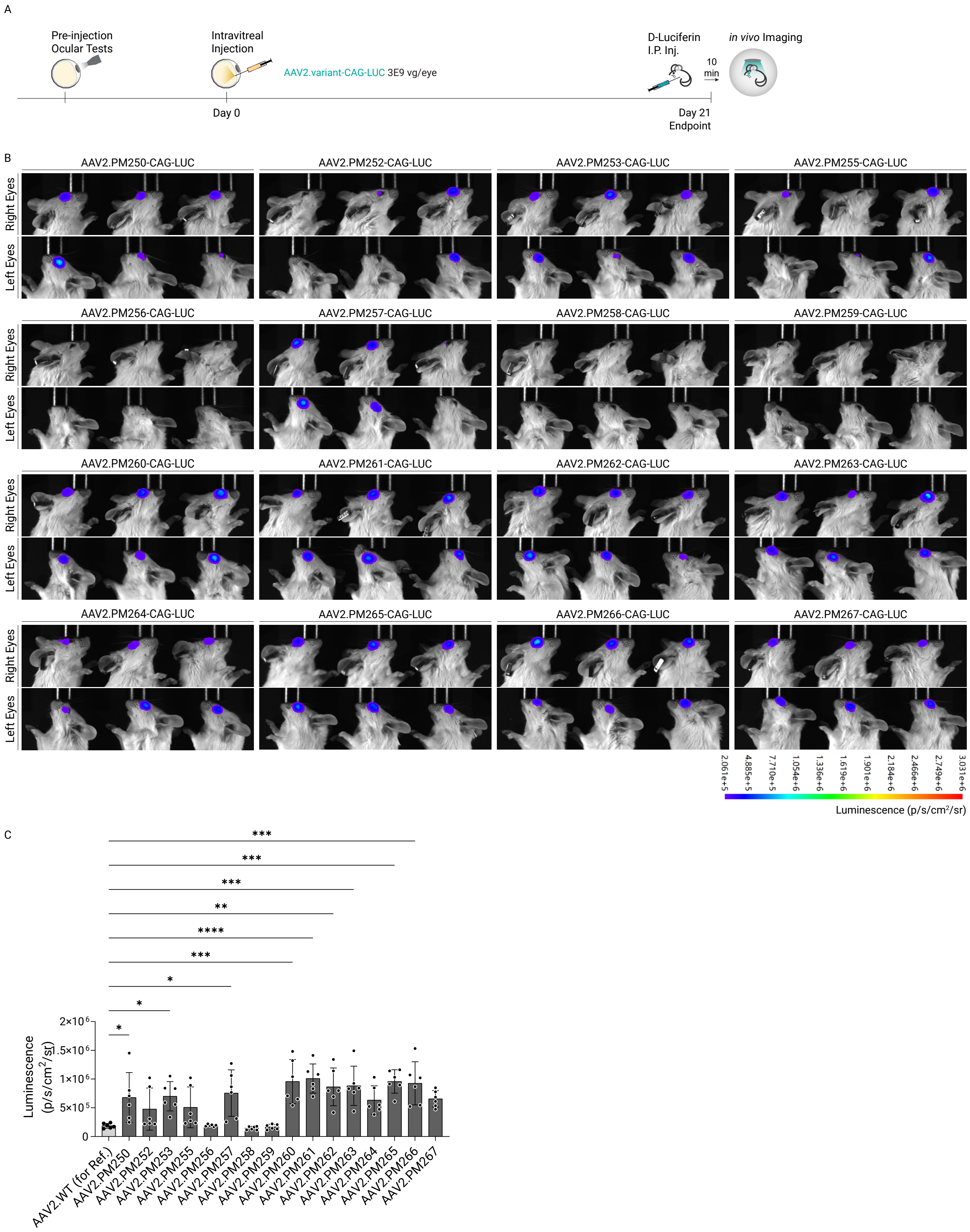
A
Pre-injection Ocular Tests
Intravitreal Injection
AAV2.variant-CAG-LUC 3E9 vg/eye
Day 0
D-Luciferin I.P. Inj.
10 min
in vivo Imaging
Day 21 Endpoint
B
AAV2.PM250-CAG-LUC
AAV2.PM252-CAG-LUC
AAV2.PM253-CAG-LUC
AAV2.PM255-CAG-LUC
AAV2.PM256-CAG-LUC
AAV2.PM257-CAG-LUC
AAV2.PM258-CAG-LUC
AAV2.PM259-CAG-LUC
AAV2.PM260-CAG-LUC
AAV2.PM261-CAG-LUC
AAV2.PM262-CAG-LUC
AAV2.PM263-CAG-LUC
AAV2.PM264-CAG-LUC
AAV2.PM265-CAG-LUC
AAV2.PM266-CAG-LUC
AAV2.PM267-CAG-LUC
Right Eyes
Left Eyes
2.061e+5
4.885e+5
7.710e+5
1.054e+6
1.336e+6
1.619e+6
1.901e+6
2.184e+6
2.466e+6
2.749e+6
3.031e+6
Luminescence (p/s/cm²/sr)
C
***
***
***
**
****
***
*
*
*
Luminescence (p/s/cm²/sr)
2×10⁶
1.5×10⁶
1×10⁶
5×10⁵
0
AAV2.WT (for Ref.)
AAV2.PM250
AAV2.PM252
AAV2.PM253
AAV2.PM255
AAV2.PM256
AAV2.PM257
AAV2.PM258
AAV2.PM259
AAV2.PM260
AAV2.PM261
AAV2.PM262
AAV2.PM263
AAV2.PM264
AAV2.PM265
AAV2.PM266
AAV2.PM267

Figure S6. Luciferase-based *in vivo* imaging of mice treated with AAV2.variant-CAG-LUC. A, Experimental design. **B**, *in vivo* imaging following D-luciferin injection into mice administered with different luciferase-expressing AAV2-derived variants. **C**, Quantification of luminescence from the ocular region upon D-luciferin administration. n = 6 eyes (**A**-**C**). Dot plots show mean (SD). One-way ANOVA followed by Dunnett's multiple comparisons test (**C**: $F_{(16, 85)} = 6.739$, $p < 0.0001$). *p<0.05, **p<0.01, ***p<0.001, ****p<0.0001. n.s. - no significant difference.

Figure S7

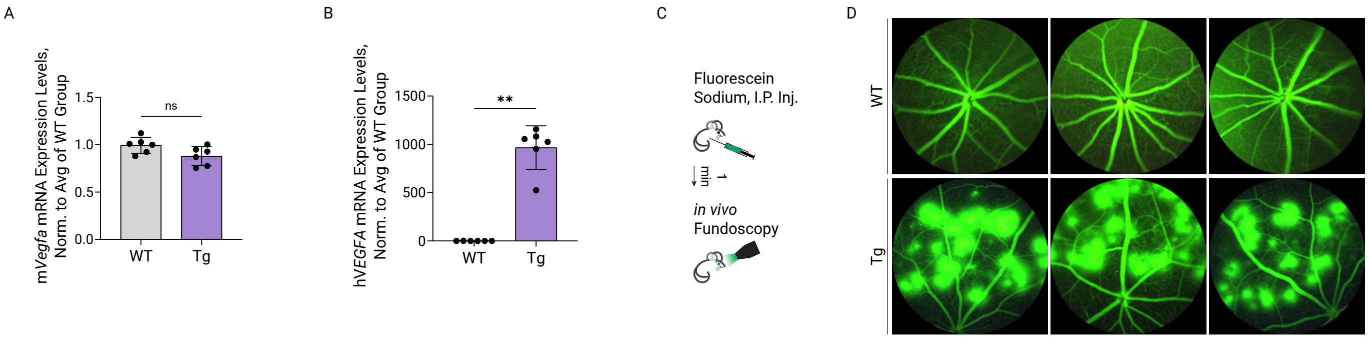
A
mVegfa mRNA Expression Levels, Norm. to Avg of WT Group
ns
1.5
1.0
0.5
0.0
WT
Tg
B
hVEGFA mRNA Expression Levels, Norm. to Avg of WT Group
**
1500
1000
500
0
WT
Tg
C
Fluorescein Sodium, I.P. Inj.
1 min
in vivo Fundoscopy
D
WT
Tg

Figure S7. Validation of *hVEGF-TG* mice. A, m*Vegfa* mRNA expression, detected using retinal tissue extracted from WT and *hVEGFA-TG* mice. **B**, h*VEGFA* mRNA expression, detected using retinal tissue extracted from WT and *hVEGFA-TG* mice. **C**, Experimental design. **D**, Representative micrographs obtained via *in vivo* fundoscopy upon fluorescein sodium injection. n = 6 eyes (**A**&**B**), n = 3 eyes (**c**). Dot plots show mean (SD). Two-tailed unpaired Student's *t*-test (**A**: $t_{(6)} = 6.025$, $p = 0.0038$). Two-sided Mann-Whitney test (**B**: $U = 0$, $p = 0.0022$). **$p<0.01$. n.s. - no significant difference.

Figure S8

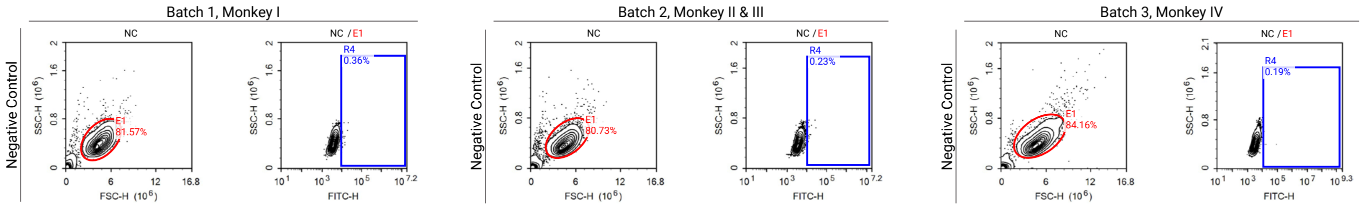

Batch 1, Monkey I
Batch 2, Monkey II & III
Batch 3, Monkey IV
Negative Control
NC
NC / E1
E1
81.57%
R4
0.36%
80.73%
0.23%
84.16%
0.19%
SSC-H (10^6)
FSC-H (10^6)
FITC-H

Figure S8. Gating strategy for FACS analysis. The gating strategy for quantifying GFP^+ AAV2.WT-CMV-eGFP/FBS transduced HEK293T cells. The negative control HEK293T cells were incubated with only DMEM. For proper gating, FSC/SSC were used to exclude cell debris and cell aggregates and select target cell population E1 for analysis. In the FITC channel, GFP^+ cells (R4) were identified by setting the boundary near the population of HEK293T cells not infected by AAV2.WT-CMV-eGFP, establishing a clear threshold to separate GFP^+ cells from GFP^- cells.

Figure S9

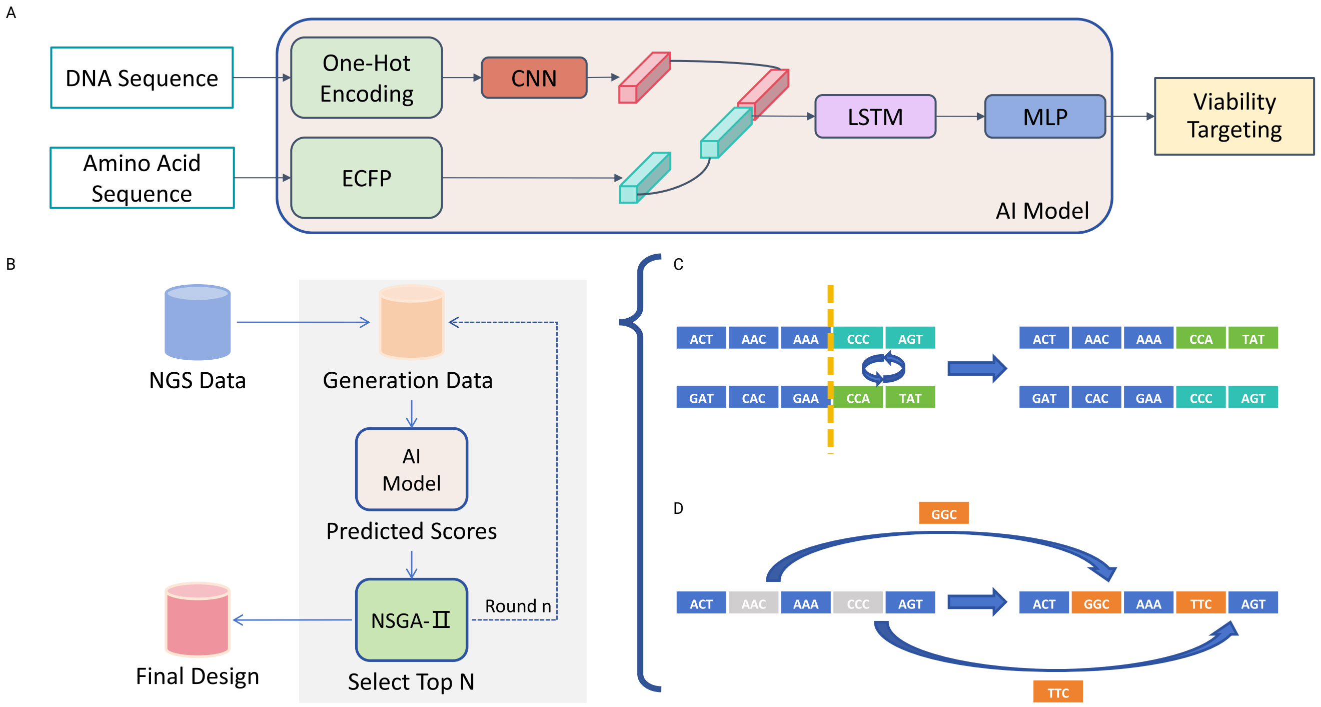
A
DNA Sequence
Amino Acid Sequence
One-Hot Encoding
CNN
ECFP
LSTM
MLP
AI Model
Viability Targeting
B
NGS Data
Generation Data
AI Model
Predicted Scores
NSGA-Ⅱ
Round n
Select Top N
Final Design
C
ACT AAC AAA CCC AGT
GAT CAC GAA CCA TAT
ACT AAC AAA CCA TAT
GAT CAC GAA CCC AGT
D
GGC
ACT AAC AAA CCC AGT
ACT GGC AAA TTC AGT
TTC

Figure S9. Visualized AI methodology. A, AI models for predicting AAV viability and targeting. **B**, Sequence generator. **C**, Fragment recombination. **D**, Base mutation.

Figure S10

A

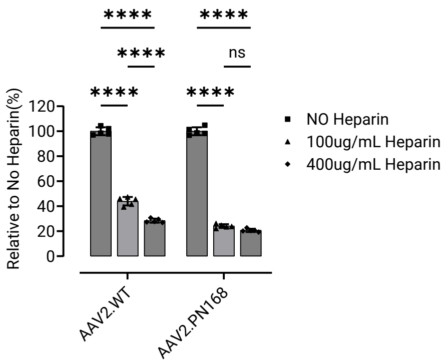

B

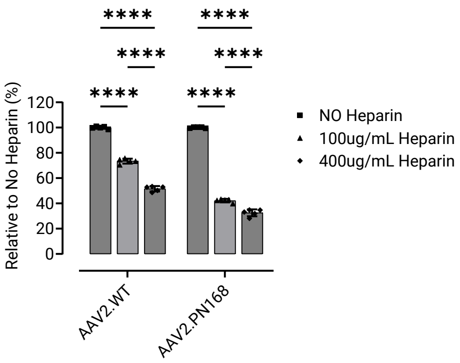

Figure S10. Heparin competition assay. A, AAV-mediated mean fluorescent intensity upon administration of heparin sodium salt. **B**, AAV-transduced GFP positive cells upon administration of heparin sodium salt.
